# Women’s reflections on timing of motherhood: a meta-synthesis of qualitative evidence

**DOI:** 10.1186/s12978-022-01548-x

**Published:** 2023-02-08

**Authors:** Camilla Gry Temmesen, Tove Faber Frandsen, Henriette Svarre-Nielsen, Kathrine Birch Petersen, Jane Clemensen, Heidi Lene Myglegaard Andersen

**Affiliations:** 1 Department of Nutrition, Rehabilitation and Midwifery, University College Absalon, Sdr. Stationsvej 30, 4200 Slagelse, Denmark; 2 Clinical Institute, University of Southern Denmark, Odense, Denmark; 3 Department of Design and Communication, University of Southern Denmark, Kolding, Denmark; 4 Department of Clinical Medicine, University of Copenhagen, Copenhagen, Denmark; 5 Department of Obstetrics and Gynecology, Copenhagen University Hospital Hvidovre, Hvidovre, Denmark; 6 Specialist in Fertility, Obstetrics and Gynaecology, Copenhagen, Denmark; 7 Department of Children, Hans Christian Andersen Children’s Hospital, University of Southern Denmark, Odense, Denmark; 8 Department of Nursing, University College Absalon, Roskilde, Denmark

**Keywords:** Motherhood, Timing, Women, Reproduction, Reproductive age, Advanced maternal age, Postponing, Meta-synthesis, Systematic review, Qualitative evidence synthesis

## Abstract

**Introduction:**

Fertility declines with increasing age, especially in women. In recent decades women’s age at the birth of their first child has risen markedly in many countries, and an increasing number of women do not establish a family until their late-twenties to mid-thirties. Although there can be various reasons that couples experience fertility problems, advanced maternal age is the most frequent cause for difficulties with achieving pregnancy.

**Objective:**

In this meta-synthesis, we investigated reflections on timing of motherhood in women who have not yet had children.

**Methods:**

A systematic literature search of six electronic databases and manual searches of reference lists identified eight qualitative studies published between 2011 and 2018 that focused on women’s reflections on timing of motherhood. The studies were assessed with the Critical Assessment Skills Programme (CASP) quality appraisal tool. The results were synthesized using Noblit and Hare’s meta-ethnographic approach as described by Malterud.

**Findings:**

An overall theme of ‘*Timing of motherhood*’ and four overlapping subthemes were identified: *Making a life-changing decision*, *The right time*, *Fear of regret*, and *Plan B*. The dilemmas associated with timing of motherhood leave women of reproductive age balancing their priorities and values against a biological deadline for having children naturally or through assisted reproductive technology.

**Conclusions:**

Women of reproductive age are aware that they must make a life-changing decision as to *if* or *when* to have children, but they consider having children at ‘the right time’ to be important. Simultaneously, while some women are reluctant to have children for various reasons, they express fear that waiting too long could result in their regretting not having children later in life. Although women of reproductive age express concern about their ability to achieve pregnancy, they have limited focus on the medical risks associated with postponing motherhood. There is a need to establish preventive health initiatives to support women of reproductive age in their considerations regarding timing of motherhood.

Trial registration number: PROSPERO: CRD42020175151.

**Supplementary Information:**

The online version contains supplementary material available at 10.1186/s12978-022-01548-x.

## Plain language summary

In many countries, women’s age at the birth of their first child has risen markedly, and an increasing number of women do not establish a family until their late-twenties to mid-thirties. This causes risk of couples experiencing fertility problems, as fertility declines with increasing age. Although fertility problems can be caused by various reasons, advanced maternal age is the most frequent cause for women having difficulties achieving pregnancy. In this study, we investigated reflections on timing of motherhood in women who have not yet had children. Through a systematic literature search we identified eight qualitative studies published between 2011 and 2018 that focused on women’s reflections on timing of motherhood. The included studies were synthesized using a meta-ethnographic approach. We identified an overall theme ‘*Timing of motherhood*’ and four overlapping subthemes: *Making a life-changing decision*, *The right time*, *Fear of regret*, and *Plan B*. The dilemmas associated with timing of motherhood leave women of reproductive age balancing their priorities and values against a biological deadline for having children naturally or through assisted reproductive technology. Women of reproductive age are aware that they must make a life-changing decision as to *if* or *when* to have children, but they consider having children at ‘the right time’ to be important. Simultaneously, while some women are reluctant to have children for various reasons, they express fear that waiting too long could result in their regretting not having children later in life. Although women of reproductive age express concern about their ability to achieve pregnancy, they have limited focus on the medical risks associated with postponing motherhood, which emphasizes the need for establishing preventive health initiatives to support women of reproductive age in their considerations regarding timing of motherhood.

**Supplementary Information:**

The online version contains supplementary material available at 10.1186/s12978-022-01548-x.

## Introduction

Due to the considerable decline in fertility that occurs with increasing age, age constitutes a substantial risk factor for infertility [1–3]. With rising age, women and men have a higher risk of having diseases that can reduce fertility directly or whose treatments impact fertility, such as diabetes, hypertension, and cancer. Lifestyle factors that affect fertility, such as being overweight, smoking, exposure to sexually transmitted diseases, and the use of hormone disrupting drugs, also become increasingly impactful with age [2, 3].

Female fertility is especially age sensitive as the amount and quality of egg follicles declines with age, which can result in chromosomal abnormalities [4] and miscarriages [3, 5]. A woman’s ability to conceive decreases moderately starting from the mid-20 s, followed by the onset of a drastic decline in the mid-30 s. A 20-year-old healthy woman has a 34% probability of becoming pregnant per cycle. By 30 years of age, this probability is halved to 17% and then falls rapidly to 8% by 37 years of age. At 45 years, the chance of a woman achieving a live birth is as low as 0.5% per cycle [1, 2]. Conception becomes unlikely by approximately 10 years before menopause (average age for menopause is around 51 years), at which time the menstrual cycle becomes irregular and the biological window for childbearing is rapidly concluding [6].

Women’s age at the birth of their first child has risen markedly in recent decades, particularly but not exclusively in Western countries. An increasing number of women are not establishing a family until they are in their late twenties or thirties (Table 1), at which time a woman’s reproductive capacity has already declined significantly relative to its peak.

**Table 1:** Women’s age at birth of 1^st^ child

Country	Mean age
Bulgaria	26.4^a^
USA	27.1^b^
Romania	27.1^a^
Slovakia	27.2^a^
Finland	29.5^a^
Sweden	29.7^a^
Denmark	29.8^c^
Norway	29.8 ^b^
Luxembourg	31.0^a^
Spain	31.2^a^
Italy	31.4^a^
Korea	32.3^b^

^a^Eurostat (2020)[7]

^b^OECD Social Policy Division, Directorate of Employment, & Labour and Social Affairs (2020)[7]

^c^ Statistics Denmark (2021)[8]

Within the 27 countries in the European Union (2020), the average age of having one’s first child is 29.5 years, with the lowest average ages being found in eastern Europe (26.4 years in Bulgaria) and the highest average age in central Europe (Luxembourg 31.0 years) and southern Europe (Italy 31.4 years), respectively [7]. In northern Europe, the average first-childbirth age has never been higher, reaching 29.8 years in Denmark [8]. This pattern of women postponing motherhood into their late twenties is now common among Nordic countries [7]. Outside of Europe, first-childbirth age was found to be relatively low in the USA at 26.8 years and relatively high in Korea at 31.6 years [9].

When women delay childbearing, they risk having fewer children than what they intended, and they are at risk of needing unanticipated infertility treatments [3]. Although there can be various reasons that couples experience fertility problems, advanced maternal age is unconditionally the most frequent cause for women experiencing difficulties in achieving pregnancy [10].

Advancements in reproductive medicine during the last decade, such as easier access to elective oocyte preservation, may influence women’s timing of motherhood [11, 12]. Meanwhile, choosing single motherhood has become more socially acceptable [13]. At the same time, new initiatives and an increased focus on fertility awareness and preconception care have emerged in the form of fertility campaigns and a growing media interest in the topic [14, 15]. Despite the developments that have occurred within the reproductive field, there has been little impact on health-preventive initiatives targeting women of reproductive age who have not yet had children.

Women’s thought processes around delayed motherhood have been shown to be multifaceted and influenced by biological, psychological, social, and developmental factors [16, 17]. In a 2010 meta-synthesis focusing on the reasoning for and experiences with delaying childbearing in women 30 years old and older, Cooke et al. [18] found that women delay childbearing for various reasons and that women may be informed or uninformed regarding their family planning timing decisions. To our knowledge, no prior meta-synthesis has focused on how women across the typical reproductive age span (18–45 years) who had not yet had children reflect on the timing of motherhood. Thus, there is a need for more knowledge regarding how women view the timing of motherhood across the reproductive age spectrum to gain a deeper understanding of family planning phenomena as a foundation for establishing new preventive health initiatives in the field of reproductive medicine. The aim of this study was to investigate reflections on timing of motherhood in women who have not yet had children, to accomplish more knowledge of women’s perspectives of timing of motherhood before implementing new preventive health initiatives within the reproductive field.

## Methods

A meta-synthesis is a qualitative systematic literature review that summarizes qualitative studies within a specific topic of interest with the purposes of deepening understanding and cultivating new knowledge [19, 20]. A meta-synthesis thus aggregates the findings in a manner that extends beyond the summing of findings of the individual studies [20]. Existing studies are used as resources, thus minimizing research waste and ensuring that research is sustainable [19]. We used Malterud's [19] meta-synthesis methodology of synthesizing qualitative studies, which was inspired by Noblit and Hare’s seven-step inductive and interpretative approach to meta-ethnography (Box 1)[19, 21]. A core principle within meta-ethnography is to translate the results from qualitative primary studies into each other, by using *metaphors*. The goal is to gain a deeper and broader understanding by finding differences or similarities between studies and by combining findings from qualitative studies and then integrating the findings into a new whole. In practice, the meta-ethnographic approach is an iterative process, meaning that steps can overlap and be repeated [21].

Box 1: the seven steps of meta-ethnography (Noblit and Hare, 1988)

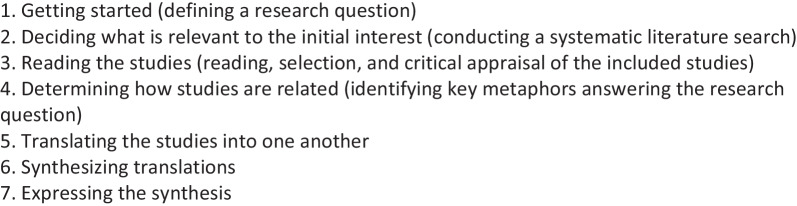

The study protocol was registered in PROSPERO (no. CRD42020175151). To enhance transparency in reporting of the meta-synthesis, this study follows the ENTREQ statement [22] (Additional file 1: Appendix A ENTREQ Checklist).

We used the PICo mnemonic (***Population, Phenomenon of Interest*** and ***Context***) from Joanna Brigg’s Institute guide to Systematic Reviews of Qualitative Evidence [20] to target the search to answer the following study question: *What are women’s reflections on timing of motherhood?* (Box 2).

Box 2: PICo

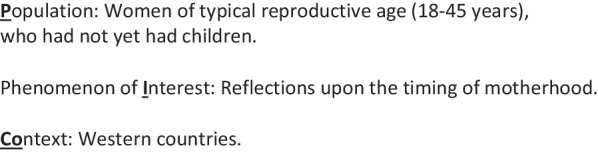

**Systematic literature search**

A systematic literature search was conducted between January 15th and January 28th 2021, with no imposed start date or specific period, in six electronic bibliographic databases*: Medline, PsycInfo, Embase (Ovid), Cinahl (Ebsco), Scopus*, and *ProQuest Dissertations & Theses Global*. The search was repeated January 13th, 2022 and November 11th 2022. The searches were adapted to fit each database using free text and subject headings [e.g. MeSH terms (MedLine), Cinahl Headings (Cinahl) and Emtree (Embase)] combined with the Boolean phrases “AND” and “OR”. We targeted the search specifically towards qualitative studies by employing experience-based multifaceted qualitative filters adjusted to each database [23–25]. We applied a language limitation, restricting the results to publications in English and the Scandinavian languages Danish, Swedish, and Norwegian, as these were within the linguistic competences of the author group. We searched for grey literature (e.g. dissertation theses in *ProQuest Dissertations & Theses Global*) [26, 27] and conducted additional manual searching through back chaining of the reference lists of the studies selected for critical appraisal. The complete systematic literature search is detailed in Additional file 2: Appendix B Systematic Literature Search. Inclusion criteria were empirical qualitative primary studies focusing upon women’s reflections on timing of motherhood. To ensure cultural homogeneity, we included studies from Western countries, including countries in Europe, the USA, Canada, Australia, and New Zealand, focusing on healthy heterosexual women 18–45 years old who had not yet had children. We defined the term healthy as the absence of pre-existing diseases, such as cancer, diabetes, hypertension, or psychological disorders, which potentially can affect the reproductive health of a woman.

**Selection and critical appraisal of the included studies**

**Fig. 1:**
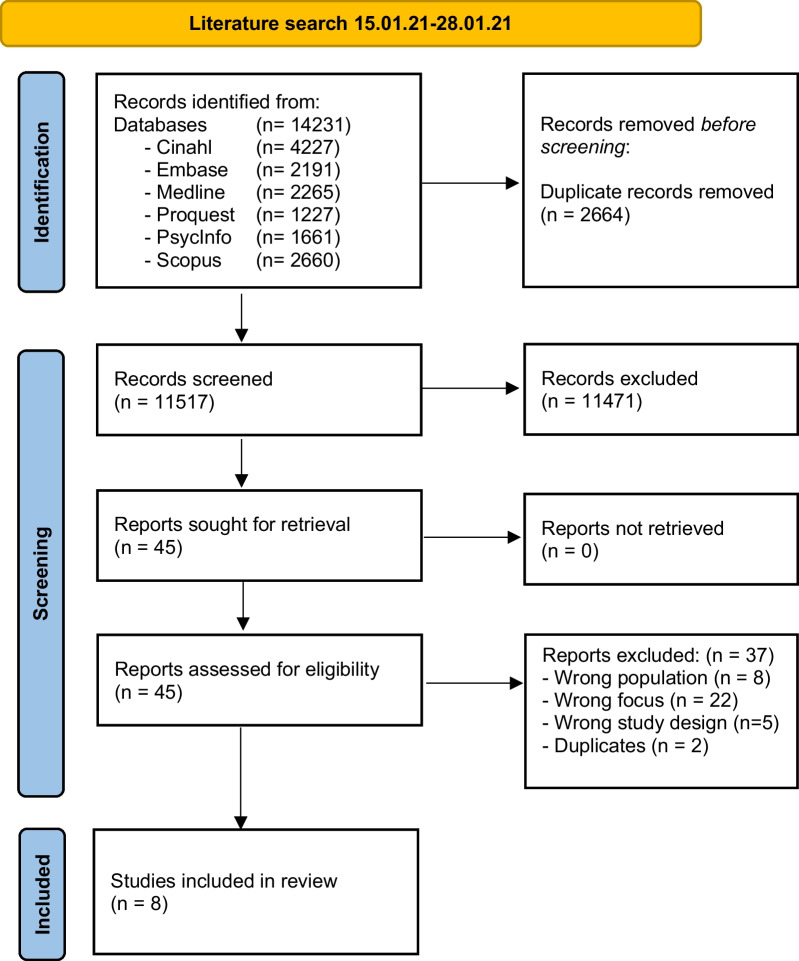
Prisma flowchart

We followed the four PRISMA (*Preferred Items for Systematic Reviews and Meta-analyses*)[28] steps of systematic search (*Identification, Screening, Eligibility, and Inclusion*) presented in a flowchart (Fig. 1) and is further detailed in Additional file 3: Appendix C PRISMA Flowchart.

**Identification**

The systematic literature search was conducted by the first author (CGT) with the assistance of an experienced information specialist (TFF).

**Screening**

Title- and abstract-level screening were conducted independently by the first (CGT) and last (HMA) authors using Covidence software [29]. Both reviewers were blinded, meaning that one could not see the other reviewer’s recommendation before giving their own [30]. Any disagreements regarding inclusion of articles were discussed until a consensus was reached. If consensus was not possible a third reviewer (JC) was consulted. Studies excluded in the full-text screening were excluded due to having a non-target focus, a wrong study design, or not fulfilling the inclusion criteria (Additional file 4: Appendix D Excluded studies and Interrater reliability).

**Eligibility**

Included studies were evaluated in accordance with the Critical Assessment Skills Programme (CASP)[31] by two authors (CGT and HMA). The CASP checklist for qualitative studies contains of ten systematic assessment questions addressing the following areas: (I) aim; (II) methodology; (III) research design; (IV) recruitment strategy (V); data collection; (VI) relationship between researcher and participants; (VII) ethical issues; (VIII) data analysis; (IX) findings; and (X) contribution to research area [32]. None of the studies were excluded due to the quality appraisal as all studies met the CASP quality standards and contributed with valuable data to the aim. The included studies are presented in Table 2.

**Inclusion**

Finally, after a critical appraisal assessment, the studies which met the inclusion criteria were considered eligible for inclusion in the meta-synthesis.

**TABLE 2:** Studies included in the meta-synthesis: Study characteristics and contribution to findings

Author year Country	Aim	Method Data collection Analytical approach	Setting	Participants Age (n)	Relationship status	Ethnic origin	Education/Vocational training	Contribution to findings^a^ 1 2 3 4
Sylvest et al. 2018 Denmark	To explore how attending a fertility assessment influenced women’s family planning decisions	Individual interviews Qualitative content analysis	Women interviewed one year after an individual fertility counselling at a Fertility Assessment and Counselling Clinic	20 women 35–40 years	Single 5 Cohabiting 15	Not reported	Long^b^ 16 Medium^b^ 3 Short^b^ 1	X X– –
Birch Petersen et al. 2016 Denmark	To explore attitudes towards family formation and potential differences between single and cohabiting women	Individual interviews Qualitative content analysis	Women with no known fertility problems who attended a Fertility Assessment and Counselling Clinic for assessment and counselling on future fertility	20 women ≥ 34 years	Single 10 Cohabiting 10	Not reported	Long^b^ 16 Medium^b^ 3 Short^b^ 1	X X X X
Ngyuen 2016 USA	To examine how relationship status impact motherhood motivation	Individual interviews Qualitative data analysis	College-educated^c^ aspiring professional women without children but with a desire for children in the future	12 women 30–34 years	Single 7 Married 3 Divorced 1 Engaged 1	Hispanic 2 Caucasian 6 Asian american 3 Africa american 1	BA 2 MA 8 PhD 1 JD 1	X X X X
Lavender et al. 2015 UK	To gain an understanding of women’s views surrounding decisions on the timing of childbearing	Individual interviews Thematic analysis	Young, educated women, women at an age when they are most likely to begin childbearing, and women in the category of advanced maternal age	18 women 18–24 years (6) 25–34 years (6) ≥ 35 years (6)	Single 9 Partner 4 Married 5	White British 17 White European 1	A level 2 Degree 12 PhD 1 GCSE 1 Masters 1 College 1	X X X X
Eriksson et al. 2013 Sweden	To gain a more comprehensive understanding of how young, highly educated women and men ^c^ without children, who had started professional careers, reflect on fertility and postponed parenthood	Individual interviews Content analysis	College-educated^c^ participants between 24 and 38 years of age who had started a professional career and had not yet had children	22 women 25–38 years	Single 6 Cohabiting 10 Married 6	Not reported	College 22	X X – X
Eriksson et al. 2012 Sweden	To gain a deeper understanding of how highly educated women and men^d^ without children reflect on having children in the future	Individual interviews Content analysis	College-educated ^c^ participants from three university towns, who had started their professional careers at a workplace where the majority of the staff were also college educated	22 women 25–38 years	Single 6 Cohabiting 10 Married 6	Not reported	College 22	X X – X
Cooke et al. 2012 UK	To gain an understanding of factors influencing women’s decisions to delay childbearing	In depth semi-structured interviews Hermeneutic thematic analysis	Non-pregnant women with no children, women pregnant with their first child, and women with no children attending a fertility clinic ^e^	6 women ≥ 35 years	Single 2 Partner 1 Married 3	Not reported	School/College 1 Degree 4 Masters or above 1	X X X X
Söderberg et al. 2011 Sweden	To describe fertility experiences of young women who had not been pregnant or become mothers	Individual interviews Phenomeno-logical analysis	Women who visited an outpatient clinic for contraceptive counseling	10 women 23–27 years	Not reported	Not reported	Not reported	X X X –

^a^Overall theme: Timing of motherhood. Subthemes: (1) Making a life-changing decision, (2) The right time, (3) Fear of regret, and (4) Plan B

^b^Long: Four years or more of vocational training, Medium: 2–3 years of vocational training, Short: One year or less of vocational training.

^c^At least 4 years of university education/bachelor's degree.

^d^Men’s views were clearly separated and left out of the analysis of this meta-synthesis.

^e^Only non-pregnant women were included in the analysis of this meta-synthesis.

**Analytical approach**

We followed the meta-ethnographic steps described by Malterud [19] and as outlined by Noblit and Hare [21] (Box 1). The published results of the primary studies were considered *first order analyses*. The synthesis and interpretation of the included studies conducted by the authors of the present study were considered *second order analyses*, which were conducted by reading results sections of the primary studies closely and identifying key metaphors from each original study, which in different ways answered the research question.

The analytical processes were reviewed by all authors to minimize the influence of the synthesizer.

We initiated the synthesis by identifying an *index paper*—a study that excels in being content-rich and presenting high methodological quality [19]—and used the observations as a starting point for the synthesis. Subsequent studies were added to the pre-existing categories and new categories were created when necessary. We developed a matrix listing the key metaphors and concepts from each study, which enabled us to get an overview of how the findings were related. As the empirical data in the primary articles were comparable, we made a *reciprocal translation* by coding each study line-by-line; new themes emerged in a new interpretation. Some concepts were inspired directly and verbally from the primary studies, which according to Noblit and Hare (1988) is considered acceptable because sometimes the original study uses a metaphor that already expresses a topic optimally [19, 21]. Not every primary study fed into all themes, and sometimes findings fed into more than one theme (Table 2). We did not use specific computer software for the analysis.

## Results

A total of n = 20.361 studies were initially identified through the systematic literature search, and after n = 6.989 duplicates were removed using EndNote™ X9 and Covidence software, n = 13.372 studies remained for screening. Title- and abstract-level screening retained n = 49 studies for full-text reading. A total of eight studies (n = 8) met the inclusion criteria and were considered eligible for inclusion in the meta-synthesis.

### Sample characteristics

The eight included studies were published from 2011 to 2018 and originated from Sweden (n = 3) [33–35], Denmark (n = 2)[36, 37], the UK (n = 2)[38, 39], and the USA (n = 1)[17]. All eight studies were qualitative primary studies based on individual in-depth interviews and a variety of qualitative analytical approaches (e.g. phenomenological or hermeneutic) that were focused on women’s reflection on the timing of motherhood. Sample sizes varied from 10 to 22 participants, with a total of 108 women included in this meta-synthesis (the two studies by Eriksson et al. [33, 35] were based on the same sample size). Age range among participants in the included studies varied from 18 years to ≥ 35 years (with the highest age at 50 years in one study [39]. Socio-economic characteristics such as race, ethnicity, income, level of education and relationship status were reported differently in the included studies, making a comparison difficult. However, among the included studies there was a preponderance of participants who were in a relationship (53%), defined as either cohabiting (n = 35), married (n = 17), engaged (n = 1) or having a partner (n = 5) compared to participants who did not have a partner (37%), who either were defined as being single (n = 39) or divorced (n = 1). In the study by Söderberg et al. (2011) relationship status was not reported [34].

Some of the studies had limitations that were not addressed in the critical quality appraisal. In particular, the two studies by Eriksson et al.[33, 35] which included both women and men. However, both studies were included in the meta-synthesis because of the strong focus on the study question and because men's and women's views were clearly divided, which made it possible to extract the women’s perspectives only for the analysis. In the study by Sylvest et al.[36], the participants had visited a fertility assessment and counseling clinic a year earlier to get a fertility status, thus leading us to presume that they had a special interest in the topic and knowledge regarding their own fertility statuses before entering the study. However, this was not considered a weakness due to the inclusion criteria of this meta-synthesis. In the study by Cooke et al.[39], women without children, women pregnant with their first child, and women attending a fertility clinic were included. The study was included in the meta-synthesis because the participants were clearly divided into three specific subgroups, leaving the perspectives from women with no children eligible for inclusion in this meta-synthesis.

## Findings

**Fig. 2:**
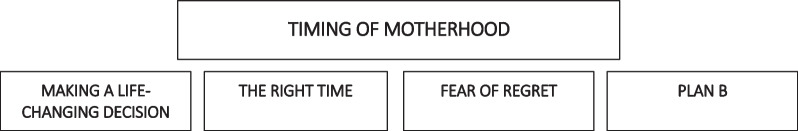
Themes derived from the meta-synthesis

The synthesis of the eight included studies led us to identify an overall theme of *‘Timing of motherhood’* and four overlapping subthemes: (1) *Making a life-changing decision*; (2) *The right time*; (3) *Fear of regret*; and (4) *Plan B* (Fig. 2)*.*

### Making a life-changing decision

Having children is considered an extremely important decision that will change and shape one’s life (34, 35, 38): ‘*It’s a permanent and life-changing decision, so it bears thinking about more than just once’ (Woman, age 35)*[35]. Increasing female age creates a pressure to decide on when to have children [17, 33, 36, 37]: *‘Biologically I’m at an age where I need to be thinking about it and motivations are that I do want a child. I do want someone to be there when I’m older so someone can take care of me…’ (Woman, age 34) *[17]. Women reflect upon on the risks they are taking if they wait too long to pursue motherhood [33]: *‘It is mostly my own age that I think about because the older you get, the harder it becomes to get pregnant, and that is the risk one takes when one waits’ (Woman, age 34)* [33], and others want ‘to buy more time’ before making the decision on when and who to have children with [37, 39]: *‘I’m not getting any younger and I don’t know if I can have children. It is probably pretty essential to figure out, whether it is a possibility at all. And how much time one has got’* (*Woman, age 35*) [37].

Women want to create ‘a perfect life’[34] in which they are able to provide for a child and to be ‘the best mother possible’ [38] before deciding when to have children*: ‘I will have children at some point but I would make a lousy mum at the moment. When I have children, I want to give them everything they need, including my time. I don’t feel settled enough yet…it just wouldn’t be fair on them’ (Woman, age 30)* [38].

Meeting the right partner to have children with is considered very important and a major consideration in the decision to have children [34–39]: *‘I am leaving it later than I would have chosen to do... I would have done it in my early 30’s if I’d been with the right person’ (Woman, age 38) *[39]. However, for some women, despite being in a stable relationship, timing of motherhood is yet a challenging decision [17]. Some women describe not having a longing for motherhood and feeling an ambivalence as to whether to have children at all [36]: ‘*We just have to make some kind of decision, but we can’t. So, it’s such an evil limbo, where you almost hope that time expires, because then there will be some closure’* (*Woman, age 39*) [36].

### ‘The right time’

Importance is placed on having children at ‘the right time’, particularly with respect to completing one's education and having stable finances before establishing a family [17, 33, 34, 37]: *‘The correct order is: education, job, and children and in addition to have a stable financial situation. To get the feeling that you have established that ‘‘package’’, which you safely and soundly can fit a child into (…)’ (Woman, age 34)* [37]. Becoming a mother at ‘the right time’ is related to a certain set of circumstances, which the women feel is a prerequisite to start a family. For example, women value having met the right partner to have children with [34–39] and having a secure job [38, 39]: *‘I really thought that I would have at least 2 children by now. That was the plan, but I just didn’t meet someone that I wanted children with. Now I am in a stable relationship, and we have talked about starting a family once I am settled into my job’ (Woman, age 23)* [38]. When talking about *when* to have children some women express an age-specific deadline [17, 38]: *‘I am very focused in my mind that I want children before I’m 30. The only thing that would put me off is if I didn’t have a stable partner…. but I would keep going and going…. even until 40 [laughs]’ (Woman, age 22)* [38]. However, ‘the right time’ for having children is not highly age-specific for most women [35, 39]: *‘So when is the right time, that’s the eternal question, and everyone says “one day you’ll just feel it’s the right time”, but I can’t say that I’ve felt that yet’(Woman, age 36)* [35]. It is questioned if there will ever be a ‘right time’ for having children [17]: *‘I think there’s never going to be a perfect time. People make it work (…)’(Woman, age 30)* [17].

Naturally, awareness of the female age-related reproductive limitations for women with advanced maternal age constitute a biological deadline for having children in women’s consciousness [17, 38, 39]: *‘Starting before 40 for sure. I think it will be harder after 40, just statistic-wise and medically a lot of women struggle more having children after 40 or as they’re getting closer to getting 40. I know there are a lot more medical routines and just screenings and testing that takes place once you’re 35 I think’ (Woman, age 34)* [17]*.* However, the medical risks associated with having children at advanced maternal age do not receive as much attention as declining fertility [38].

### Fear of regret

There is fear of regretting one’s choices in life when reflecting upon when to pursue motherhood [17, 34, 37–39]: *‘(…) If I cannot have children, then I might regret that I waited so long. But as it is now, I would not like to have children or a partner, but it is just the fact that it is unfair also that it will end sometime’ (Woman, age not reported)* [34]*.* When women envision their future older selves looking back at their lives, the fear of regretting not having children is profound [38, 39]: ‘*It’s more this thing if I don’t have a baby ever, I’ll be sat there in my chair when I’m 70 you know thinking “ooohhh what could have been*’ (*Woman, age 42*) [38]. Another perspective of postponing motherhood is a fear of being too old to have the opportunity to experience grandchildren [39]: *‘I think one of the things that... made me feel a bit sad... was... when they’re 20... I’ll be 60... it just really put it into perspective for me... I may not see their kids’ (Woman, age 37)* [39]*.* The fear of regret also included women questioning decisions made in their lives when there may have been opportunities to have children [17, 39]: *‘You can’t look back but there’s always that regret’* (*Woman, age 42*) [39].

### Plan B

When women realize that having biological children could be challenging due to advanced age, medical fertility challenges, or the lack of a partner, they express an openness towards a ‘Plan B’ as an alternative way of pursuing motherhood, such as using assisted reproductive technologies, adoption, or surrogacy [17, 33, 38]: *‘Obviously I would like to conceive naturally but if this doesn’t happen in time then I know that I can get help….IVF. Women of all ages have babies now so, you know, I don’t see why I should be any different’ (Women, age 34)* [38]. For some women, solo motherhood is a consideration if they haven’t met the right partner to have children with [35, 37]: *‘For many years, I have been searching for a man with whom I could have children with. I haven’t succeeded in finding the one with whom I could start a family, so now I’m considering whether to do things in a different order’ (Women, age 38)*[37]. Women who are not having children emphasize the importance of having children of family and friends in their lives [38, 39]: *‘Having contact with children in their extended family or amongst friends [may help] to compensate for not having their own children’* (fieldnote) [39].

## Discussion

This meta-synthesis has revealed multiple perspectives on the timing of motherhood from a sample of women representing the typical reproductive age spectrum. The findings bring a deeper understanding of women’s reflections on the topic from the merged findings of eight qualitative studies. We identified an overall theme *‘Timing of Motherhood’* and four overlapping subthemes: *Making a life-changing decision*, *The right time*, *Fear of regret* and *Plan B.* Women of reproductive age who have not had children are aware that they must make a life-changing decision as to whether and when to have children. At the same time, women state the importance of having children at ‘the right time’. Conversely, while women express reluctance about having children for various reasons, they also express fears that they will have to live with the risk that having children in the future can be complicated or that it will become too late to do so. In that case, they are aware that they later in life will have to live with the possibility of regretting not having children.

In a prior meta-synthesis focused on factors affecting decisions to delay childbearing among women of advanced maternal age that explored women’s perceptions of associated risks, Cooke et al. [18] observed that women appear to face an issue of ‘informed and uninformed decision making’ regarding the risks of delaying childbearing. They found that women fell into three categories: those who believe they are informed but may not be; those who are not informed and find out they are at-risk once pregnant; and those who are well-informed but choose to delay pregnancy anyway [18]. Similarities can be drawn to the current meta-synthesis in that some of the included studies highlight dilemmas related to the timing of motherhood. For example, women express being indecisive about when to have children or whether to have children at all despite being aware that they might regret not having children when it is too late. These dilemmas leave women balancing the issue of having children at 'the right time' according to their priorities and values against the knowledge of a biological deadline for having children naturally or through assisted reproductive technology. Although some women express concern about whether it could become complicated to achieve pregnancy, interestingly, women do not express much concern about the medical risks of delaying motherhood. Hence, there is a need for further research into the relationship between women's knowledge of medical risks and timing of motherhood as well as a need for greater attention to be given to supporting women of reproductive age in planning the timing of motherhood to reduce the risk of infertility due to advanced age.

As the majority of the included studies were conducted in Europe (n = 7), the transferability of the present findings to countries outside of Europe should be considered with caution. We are aware that there is a preponderance of studies from northern European countries, with five of the included studies being from Sweden or Denmark, which are countries that are considered to have financial good conditions for families. For example, Danish parents are granted 52 weeks of paid parental leave and receive a quarterly child benefit [40], and Swedish parents are granted 68 weeks of paid parental leave [41] and receive a monthly child benefit [42]. Conversely, in the USA, there is no federally mandated paid maternity leave, only some women have the right to 12 weeks of unpaid maternity leave [43], and parents just recently have been granted a monthly child benefit [44]. Thus, direct comparisons of the importance of women’s perceptions of the financial conditions underlying family planning can be difficult. In Cooke et al.'s meta-synthesis from 2012 [18], women cite financial stability as an important factor before starting a family, a finding similar to several of the studies included in the current meta-synthesis [17, 33, 34, 37]. Therefore, it is important that future qualitative research examining women’s considerations of timing motherhood address the qualitative perspectives of economic factors surrounding having children, such as the importance of job security, parental leave opportunities, and child benefits.

### Strength and limitations

We followed a transparent and systematic procedure for a systematic review, using the PRISMA guidelines [28]. The included studies were selected and critically assessed according to CASP [32]. Study screening and selection were performed by two researchers leading to a more rigorous assessment of the findings. Another strength of this meta-synthesis is that we conducted a comprehensive search strategy of six databases, which limits the risk of missing published studies in the target field. We aimed for high sensitivity, which may have resulted in relatively low precision [45]; while this approach yields a high number of studies to be collated for screening, it ensures the search is broad enough to include essentially all relevant published studies. The adjusted qualitative filters applied to the search were not validated, but rather based on experience, which could potentially constitute a limitation to the search. Although we applied an extensive systematic literature search, additional articles not included in this meta-synthesis could provide supplementary perspectives. However, considering the rich analysis based on the included studies that was conducted, we find that this meta-synthesis has provided adequate saturation to the research question.

We excluded studies with women who defined themselves as homosexuals because they might have specific reproductive considerations that differ from those of women in a heterosexual relationship, such as the need for a sperm donor, considerations regarding shared motherhood, and limited access to fertility treatments for LGBTQ + individuals in some countries. Notwithstanding, we acknowledge that there can be similarities between heterosexual and homosexual women regarding the timing of motherhood, including the desires to have children at ‘the right time’, to be in a stable relationship, and to have financial security, as well as ambivalence towards motherhood and the fear of potentially later regretting not having children. Nevertheless, transferability between the findings of this study and homosexual women should be viewed with caution.

Further qualitative research into this topic is needed to deepen the understanding of the worldwide tendency to postpone motherhood and the complex nature of women’s reflections on the timing of motherhood, and to identify the needs of women of reproductive age regardless of nationality, religion, ethnicity, cohabitation status, and financial situation, including parental leave and child benefit opportunities. Future research focusing upon men’s reflections about timing of fatherhood would be beneficial to this topic as it may provide valuable insights to the complexity of timing of parenthood.

## Conclusion and implications for practice

Women of reproductive age who have not had children are aware that they are facing a life-changing decision as to when and whether to have children while, at the same time, giving importance to having children at ‘the right time’. Simultaneously, while being reluctant about having children for various reasons, women express fear that they may pursue having children too late and as a consequence will have to live with the possibility of regretting not having children later in life. Although women of reproductive age express concern about their ability to achieve pregnancy, there seem to be limited focus on the medical risks associated with postponing motherhood. These findings emphasize the importance of informing women who are considering delaying parenthood about the potential fertility risks related to advancing age, as well as the importance of establishing preventive health initiatives to support women of childbearing age in their family planning considerations. However, including women in the development of new preventive health initiatives to ensure that their needs are met, is important.

## Supplementary Information

**Additional file 1:** **Appendix A:** ENTREQ checklist.

**Additional file 2:** **Appendix B:** Systematic literature search.

**Additional file 3:** **Appendix C:** PRISMA Flowchart.

**Additional file 4:** **Appendix D:** Excluded studies and Interrater reliability.

## Data Availability

Appendix A: ENTREQ checklist. Appendix B: Systematic literature search. Appendix C: PRISMA Flowchart. Appendix D: Excluded studies and Interrater reliability.

## Abbreviations

CASP: Critical Assessment Skills Programme
ENTREQ: Enhancing transparency in reporting the synthesis of qualitative research
EPOC: Effective practice and organization of care
PICo: Population, Phenomenon of Interest and Context
PRISMA: Preferred items for systematic reviews and meta-analyses
