# Supplementary material for: Women’s reflections on timing of motherhood: a meta-synthesis of qualitative evidence
Source: Reprod Health. 2023 Feb 8;20:30. doi: 10.1186/s12978-022-01548-x (PMC9909900; doi:10.1186/s12978-022-01548-x)
Supplement: Supplementary file 1 — Additional file 1: Appendix A: ENTREQ checklist. [file 12978_2022_1548_MOESM1_ESM.docx]

**Additional file 1: Appendix A. ENTREQ CHECKLIST (Enhancing transparency in reporting the synthesis of qualitative research)***

| **No. Item** | **Guide Questions/Description** | **Reported on Page** |
| --- | --- | --- |
| **1. Aim** | To conduct a meta-synthesis of qualitative evidence of the reflective views of women who have not yet had children to accomplish more knowledge of women’s perspectives of timing of motherhood before implementing new preventive health initiatives within the reproductive field. | 3 |
| **2. Synthesis methodology** | Malterud's meta-synthesis methodology of synthesizing qualitative studies, inspired by Noblit and Hare’s seven-step inductive and interpretative approach to meta-ethnography, was used as a framework for the synthesis. | 3, 5  + Box 1 |
| **3. Approach to searching** | The literature search was pre-planned and systematic. Comprehensive searches were undertaken to seek all available studies.  The PICo mnemonic (***P****opulation, Phenomenon of* ***I****nterest* and ***Co****ntext*) from Joanna Brigg’s Institute guide to Systematic Reviews of Qualitative Evidence were used to target the search to answer the following study question: *What are women’s reflections on timing of motherhood?* | 4-5  + Box 2  + Figure 1 |
| **4. Inclusion criteria** | Inclusion criteria were empirical qualitative primary studies focusing upon women’s reflections on timing of motherhood. To ensure cultural homogeneity, studies from Western countries, including countries in Europe, the USA, Canada, Australia, and New Zealand were included, focusing on healthy heterosexual women 18–45 years old who had not yet had children.  A language limitation was added, restricting the results to publications in English and the Scandinavian languages Danish, Swedish, and Norwegian.  No imposed start date or specific period were applied to the search.  Studies excluded in the full-text screening were excluded due to having a non-target focus, a wrong study design (e.g. systematic reviews), or not fulfilling the inclusion criteria. | 4-5 |
| **5. Data sources** | Six electronic bibliographic databases: *Medline (PubMed), PsycInfo,* *Embase (Ovid), Cinahl (Ebsco), Scopus*, and *ProQuest Dissertations & Theses Global* were considered the most relevant databases for the topic of interest.  Grey literature (e.g. dissertation theses) was identified and additional manual searching through back chaining of the reference lists of the studies selected for critical appraisal were applied. | 4 |
| **6. Electronic Search strategy** | Appendix B describes the literature search. | Appendix B |
| **7. Study screening methods** | Title- and abstract-level screening were conducted independently by the first- and last author. Both reviewers were blinded, meaning that one could not see the other reviewer’s recommendation before giving their own. The decision to include or exclude a study was required to be agreed on by both reviewers, why any disagreements regarding inclusion of articles were discussed until a consensus was reached. If consensus was not possible a third reviewer was consulted. | 5 |
| **8. Study characteristics** | Table 2 presents study characteristics (author, year, country, aim, method, data collection, analytical approach, number of participants, setting, and contributions to findings. | 7-9  +Table 2 |
| **9. Study selection results** | PRISMA (*Preferred Items for Systematic Reviews and Meta-analyses*) guidance (*Identification, Screening, Eligibility and Inclusion*) was used to construct a flow diagram displaying the database searching process. 20.361 studies were initially identified and after 6.989 duplicates were removed, a total of 13.372 studies remained for screening. Title- and abstract-level screening retained 49 studies for full-text reading. A total of eight studies were included. Studies excluded in the full-text screening were excluded due to having a non-target focus, a wrong study design, or not fulfilling the inclusion criteria (Appendix B). | 7  +Figure 1  +Appendix B |
| **10. Rationale for appraisal** | Critical Appraisal Skills Program (CASP) guidelines were used to assess the qualitative evidence syntheses findings. The CASP checklist for qualitative studies contains of ten systematic assessment questions addressing the following areas: (I) aim; (II) methodology; (III) research design; (IV) recruitment strategy (V); data collection; (VI) relationship between researcher and participants; (VII) ethical issues; (VIII) data analysis; (IX) findings; and (X) contribution to research area. The eight included studies were evaluated by the first-and last author. | 5 |
| **11. Appraisal items** | CASP guidelines were used to qualitative appraise all included studies. | 5 |
| **12. Apprais l process** | Appraisal was conducted independently by two independent reviewers (first- and last author). If consensus was not possible a third reviewer was consulted. | 5 |
| **13. Appraisal results** | Eighth studies were considered eligible for inclusion in the meta-synthesis | 5, 8-9 |
| **14. Data extraction** | Extracted data includes: Specific characteristics of included studies (author, year, country, aim, method, data collection, analytical approach, number of participants, setting and contributions to findings). | 7  +Table 2 |
| **15. Software** | EndNote was used for reference management. Covidence software was used for title- and abstract screening. No specific software was used for analysis. | 7 |
| **16. Number of reviewers** | The analytical processes were reviewed by all authors to minimize the influence of the synthesizer. | 5 |
| **17. Coding** | Coding were carried out line-by-line and a thematic analysis was employed.  The meta-ethnographic steps described by Malterud and outlined by Noblit and Hare, were followed. The published results of the primary studies were considered *first order analyses*. The synthesis and interpretation of the included studies conducted by the authors of the present study were considered *second order analyses*, which were conducted by reading results sections of the primary studies closely and identifying key metaphors from each original study, which in different ways answered the research question. | 5  +Box 1 |
| **18. Study comparison** | The synthesis was initiated by identifying an index paper and using the observations as a starting point. Subsequent studies were added to the pre-existing categories and new categories were created when necessary. A matrix listing the key metaphors and concepts from each study was developed to get an overview of how the findings were related. As the empirical data in the primary articles were comparable, a reciprocal translation was made by coding each study line-by-line and new themes emerged in a new interpretation. | 5 |
| **19. Derivation of themes** | The process of deriving themes were inductive. | 3, 5 |
| **20. Quotations** | Quotations from the included studies which were used to construct themes is shown in under each specific theme in the Findings section. | 10-11 |
| **21. Synthesis output** | The synthesis of the eight included studies identified an overall theme of *‘Timing motherhood’* with four overlapping subthemes: 1) *A life-changing decision*; 2) *The right time*; 3) *Fear of regret*; and 4) *Plan B.*  *A life-changing decision* included an awareness of how increasing female age creates a pressure on when to have children, reflections upon on the risks they are taking if they wait too long to pursue motherhood, creating optimal conditions for motherhood or in some cases, not longing for motherhood, despite being in a stable relationship. ‘*The right time’* included a certain set of circumstances, which the women feel is a prerequisite to start a family, for example having a partner, job and financial stability. The right time is not highly age-specific and there is a sparse focus on the medical risks associated with having children at advanced maternal age. *Fear of regret* included a profound fear of regretting not having children later in life. *Plan B* included alternative ways of pursuing motherhood, such as using assisted reproductive technologies, adoption, or surrogacy. | 10-11  +Figure 2 |

* Tong A, Flemming K, McInnes E, Oliver SA, Craig J. Enhancing transparency in reporting the synthesis of qualitative research: ENTREQ. BMC Medical Research Methodology 2012, 12:181.
