# Supplementary material for: Women’s reflections on timing of motherhood: a meta-synthesis of qualitative evidence
Source: Reprod Health. 2023 Feb 8;20:30. doi: 10.1186/s12978-022-01548-x (PMC9909900; doi:10.1186/s12978-022-01548-x)
Supplement: Supplementary file 2 — Additional file 2: Appendix B: Systematic literature search. [file 12978_2022_1548_MOESM2_ESM.docx]

**Additional file 2: Appendix B. SYSTEMATIC LITERATURE SEARCH**

**ORIGINAL SEARCH 15.01.21-28.01.21**

**EMBASE (Ovid) 27.01.21**

Embase Classic+Embase <1947 to 2021 January 26>

1 Consider*.mp. 3004477

2 Thought*.mp. 391979

3 Aware*.mp. or exp awareness/ 369872

4 exp cognition/ or Cogniti*.mp. 2776083

5 exp knowledge/ or Knowledg*.mp. 1064951

6 exp attitude/ or Attitud*.mp. 917726

7 Reflect*.mp. 793114

8 Perception*.mp. or exp perception/ 629630

9 Perceiv*.mp. 296140

10 Intent*.mp. 180742

11 Opinion*.mp. 176928

12 exp "expression of concern"/ or Concern*.mp. 908941

13 Feel*.mp. 154772

14 emotion/ or Emotion*.mp. 351896

15 View*.mp. 676453

16 Preference*.mp. 222248

17 exp personal value/ or Value*.mp. 3054711

18 decision making/ or Valuation*.mp. 252116

19 Acceptab*.mp. 265704

20 Belief*.mp. 115777

21 Believe*.mp. 314540

22 Decide*.mp. 99332

23 Decision*.mp. 772494

24 Experience*.mp. 1672468

25 1 or 2 or 3 or 4 or 5 or 6 or 7 or 8 or 9 or 10 or 11 or 12

or 13 or 14 or 15 or 16 or 17 or 18 or 19 or 20 or 21 or 22

or 23 or 24 12462940

26 qualitative*.mp. or exp qualitative research/ 405123

27 exp interview/ or exp semi structured interview/ or interview*.

mp. 546523

28 finding*.mp. 3286690

29 health care organization.mp. or exp health care organization/ 1629779

30 exp ethnology/ or ethnol*.mp. 80709

31 exp ethnographic research/ or exp ethnography/ or ethnog*.mp. 13902

32 exp ethnonursing research/ or ethnonurs*.mp. 127

33 emic.mp. 757

34 etic.mp. 414

35 field note*.mp. 2903

36 field record*.mp. 760

37 fieldnote*.mp. 187

38 exp field study/ or field stud*.mp. 22613

39 exp participant observation/ or participant observ*.mp. 10266

40 participant observation*.mp. or participant observation/ 10028

41 exp hermeneutics/ or hermeneutic*.mp. 4471

42 exp phenomenology/ or phenomenol*.mp. 35427

43 lived experience*.mp. 8352

44 grounded theory.mp. or exp grounded theory/ 15756

45 exp constant comparative method/ or constant compar*.mp. 6594

46 exp theoretical sample/ or theoretical sampl*.mp. 999

47 exp content analysis/ or content analy*.mp. 40413

48 exp thematic analysis/ or thematic analy*.mp. 31292

49 exp narrative/ or narrative*.mp. 54506

50 unstructured categor*.mp. 3

51 structured categor*.mp. 26

52 unstructured interview*.mp. 1120

53 exp semi structured interview/ or semi-structured interview*.mp. 60406

54 maximum variation*.mp. 1349

55 exp snowball sample/ or snowball.mp. 3828

56 exp audio recording/ or audio.mp. 27297

57 exp tape recorder/ or tape*.mp. 81104

58 video*.mp. 216975

59 exp purposive sample/ or purposive sampl*.mp. 12135

60 action research.mp. or exp action research/ 5758

61 focus group*.mp. 62741

62 26 or 27 or 28 or 29 or 30 or 31 or 32 or 33 or 34 or 35

or 36 or 37 or 38 or 39 or 40 or 41 or 42 or 43 or 44 or 45

or 46 or 47 or 48 or 49 or 50 or 51 or 52 or 53 or 54 or 55

or 56 or 57 or 58 or 59 or 60 or 61 5772984

63 ((Timing or time or Plan* or Delay* or (late adj3 onset) or

Late-onset or Postpon* or advance*) adj3 (Motherhood or

Mothering or Parenthood or Parenting or Childbearing or

Child-bearing or (family adj2 formation*) or

(maternal adj2 age))).mp. [mp=title, abstract, heading word,

drug trade name, original title, device manufacturer, drug

manufacturer, device trade name, keyword, floating

subheading word, candidate term word] 7697

64 ((Fertility or (biological adj2 clock*) or (Age* adj2 fertil*)

or (fertil* adj2 declin*) or (reproduc* adj2 age*))

adj3 (Consider* or Thought* or Aware* or Cogniti* or

Knowledg* or Attitud* or Reflect* or Perception* or Perceiv*

or Intent* or Opinion* or Concern* or Expression of

concern or Feel* or Emotion* or View* or Preference* or

Value* or Personal value or Decision making or Acceptab*

or Belief* or Believe* or Decide* or Decision* or

Experience*)).mp. [mp=title, abstract, heading word, drug

trade name, original title, device manufacturer, drug

manufacturer, device trade name, keyword, floating subheading

word, candidate term word] 5838

65 25 and 62 and 63 1461

66 62 and 64 2158

67 65 or 66 3515

68 limit 67 to (danish or english or norwegian or swedish) 3420

69 limit 68 to (article or article in press or books or chapter

or editorial or "review") 2252

**MEDLINE (Ovid) 27.01.21**

Ovid MEDLINE(R) ALL <1946 to January 26, 2021>

1 Consider*.mp. 2132775

2 Thought*.mp. 278313

3 Aware*.mp. 249767

4 exp Cognition/ or Cogniti*.mp. 529931

5 exp Knowledge/ or Knowledg*.mp. 815492

6 Attitud*.mp. 437421

7 Reflect*.mp. 622349

8 exp Perception/ or Perception*.mp. 633091

9 Perceiv*.mp. [mp=title, abstract, original title,

name of substance word, subject heading word, floating

sub-heading word, keyword heading word, organism

supplementary concept word, protocol supplementary

concept word, rare disease supplementary concept word,

unique identifier, synonyms] 237936

10 Intent*.mp. 128392

11 Opinion*.mp. 125660

12 Concern*.mp. 641347

13 Feel*.mp. 98516

14 exp Emotions/ or Emotion*.mp. 393229

15 View*.mp. 487635

16 Preference*.mp. 175758

17 Value*.mp. 2258044

18 Valuation*.mp. 7349

19 Acceptab*.mp. [mp=title, abstract, original title,

name of substance word, subject heading word,

floating sub-heading word, keyword heading word,

organism supplementary concept word, protocol

supplementary concept word, rare disease

supplementary concept word, unique identifier, synonyms] 188928

20 Belief*.mp. 87074

21 Believe*.mp. 223962

22 Decide*.mp. or exp Decision Making/ 259734

23 Decision*.mp. or Decision Making/ 476574

24 Experience*.mp. 1127231

25 1 or 2 or 3 or 4 or 5 or 6 or 7 or 8 or 9 or 10 or 11 or 12

or 13 or 14 or 15 or 16 or 17 or 18 or 19 or 20 or 21 or 22

or 23 or 24 8697313

26 exp Qualitative Research/ or qualitative*.mp. 310507

27 interview*.mp. or exp Interview/ 408174

28 finding*.mp. 2462766

29 health care organization.mp. 1306

30 exp Qualitative Research/ or qualitative.mp. 264411

31 exp Ethnology/ or ethnol*.mp. 167620

32 ethnog*.mp. [mp=title, abstract, original title, name

of substance word, subject heading word, floating

sub-heading word, keyword heading word, organism

supplementary concept word, protocol supplementary

concept word, rare disease supplementary concept word,

unique identifier, synonyms] 11733

33 ethnonurs*.mp. [mp=title, abstract, original title, name of substance word, subject heading word, floating sub-heading

word, keyword heading word, organism supplementary

concept word, protocol supplementary concept word,

rare disease supplementary concept word, unique

identifier, synonyms] 122

34 emic.mp. 607

35 etic.mp. 234

36 field note*.mp. 2390

37 field record*.mp. 628

38 fieldnote*.mp. 149

39 field stud*.mp. 15972

40 participant observ*.mp. 4761

41 participant observation*.mp. 4580

42 hermeneutic*.mp. or exp Hermeneutics/ 3939

43 phenomenol*.mp. 27959

44 lived experience*.mp. 6936

45 grounded theory.mp. or exp Grounded Theory/ 12451

46 constant compar*.mp. 5241

47 theoretical sampl*.mp. 732

48 content analy*.mp. 32949

49 thematic analy*.mp. [mp=title, abstract, original title,

name of substance word, subject heading word, floating

sub-heading word, keyword heading word, organism

supplementary concept word, protocol supplementary

concept word, rare disease supplementary concept word,

unique identifier, synonyms] 23222

50 narrative*.mp. 53291

51 unstructured categor*.mp. 3

52 structured categor*.mp. 18

53 unstructured interview*.mp. 811

54 semi-structured interview*.mp. 33649

55 maximum variation*.mp. 1074

56 snowball.mp. 2969

57 audio.mp. 47386

58 tape*.mp. 56335

59 video*.mp. 180031

60 purposive sampl*.mp. [mp=title, abstract, original title,

name of substance word, subject heading word, floating

sub-heading word, keyword heading word, organism

supplementary concept word, protocol supplementary

concept word, rare disease supplementary concept word,

unique identifier, synonyms] 8492

61 action research.mp. 4489

62 focus group*.mp. or exp Focus Groups/ 55520

63 26 or 27 or 28 or 29 or 30 or 31 or 32 or 33 or 34 or 35

or 36 or 37 or 38 or 39 or 40 or 41 or 42 or 43 or 44 or 45

or 46 or 47 or 48 or 49 or 50 or 51 or 52 or 53 or 54 or 55

or 56 or 57 or 58 or 59 or 60 or 61 or 62 3382501

64 ((Timing or time or Plan* or Delay* or (late adj3 onset)

or Late-onset or Postpon* or advance*)

adj3 (Motherhood or Mothering or Parenthood or Parenting

or Childbearing or Child-bearing or (family adj2 formation*)

or (maternal adj2 age))).mp. [mp=title, abstract, original title,

name of substance word, subject heading word, floating

sub-heading word, keyword heading word, organism

supplementary concept word, protocol supplementary

concept word, rare disease supplementary concept word,

unique identifier, synonyms] 5972

65 ((Fertility or (biological adj2 clock*) or (Age* adj2 fertil*)

or (fertil* adj2 declin*) or (reproduc* adj2 age*))

adj3 (Consider* or Thought* or Aware* or Cogniti* or

Knowledg* or Attitud* or Reflect* or Perception* or Perceiv*

or Intent* or Opinion* or Concern* or Expression of concern

or Feel* or Emotion* or View* or Preference* or Value* or

Personal value or Decision making or Acceptab* or Belief* or

Believe* or Decide* or Decision* or Experience*)).mp.

[mp=title, abstract, original title, name of substance word,

subject heading word, floating sub-heading word, keyword

heading word, organism supplementary concept word,

protocol supplementary concept word, rare disease

supplementary concept word, unique identifier, synonyms] 4728

66 25 and 63 and 64 927

67 63 and 65 1466

68 66 or 67 2335

69 limit 68 to (danish or english or norwegian or swedish) 2265

70 limit 69 to (adaptive clinical trial or case reports or classical

article or clinical study or clinical trial, all or clinical trial, phase i

or clinical trial, phase ii or clinical trial, phase iii or clinical trial,

phase iv or clinical trial or comment or comparative study or

controlled clinical trial or "corrected and republished article"

or duplicate publication or editorial or evaluation study or

government publication or guideline or interactive tutorial or

interview or introductory journal article or journal article or

lecture or meta analysis or multicenter study or observational

study or personal narrative or preprint or randomized controlled

trial or "review" or "systematic review") 2264

**PSYCINFO (Ovid) 27.01.21**

APA PsycInfo <1806 to January Week 3 2021>

1 Consider*.mp. 558844

2 Thought*.mp. 153085

3 exp Awareness/ or Aware*.mp. 213564

4 exp Cognitions/ or Cogniti*.mp. 694899

5 Knowledg*.mp. 325495

6 Attitud*.mp. 482089

7 Reflect*.mp. 251921

8 Perception*.mp. 490860

9 Perceiv*.mp. 270740

10 exp Intention/ or Intent*.mp. 101643

11 exp Attitudes/ or Opinion*.mp. 428593

12 Concern*.mp. 310117

13 Expression of concern.mp. 73

14 exp Emotions/ or Feel*.mp. 482752

15 Emotion*.mp. 430118

16 View*.mp. 315624

17 Preference*.mp. 114316

18 Value*.mp. or exp Values/ 344567

19 Personal value.mp. or exp Personal Values/ 4818

20 Decision making.mp. or exp Decision Making/ 171579

21 Acceptab*.mp. [mp=title, abstract, heading word, table of

contents, key concepts, original title, tests & measures, mesh] 42087

22 Belief*.mp. 144719

23 Believe*.mp. 86311

24 exp Decision Making/ or Decide*.mp. 143463

25 Decision*.mp. 235533

26 Experience*.mp. 696958

27 1 or 2 or 3 or 4 or 5 or 6 or 7 or 8 or 9 or 10 or 11 or

12 or 13 or 14 or 15 or 16 or 17 or 18 or 19 or 20 or

21 or 22 or 23 or 24 or 25 or 26 3346282

28 exp Qualitative Methods/ or qualitative*.mp. 196874

29 exp Focus Group Interview/ or interview*.mp. or

exp Semi-Structured Interview/ 422496

30 finding*.mp. 842341

31 health care organization.mp. 448

32 qualitative.mp. 176771

33 ethnol*.mp. 3526

34 ethnog*.mp. [mp=title, abstract, heading word,

table of contents, key concepts, original title, tests

& measures, mesh] 30342

35 ethnonurs*.mp. [mp=title, abstract, heading word, table

of contents, key concepts, original title, tests & measures, mesh] 65

36 emic.mp. 1210

37 etic.mp. 686

38 field note*.mp. 4204

39 field record*.mp. 165

40 fieldnote*.mp. 377

41 field stud*.mp. 8548

42 participant observ*.mp. [mp=title, abstract,

heading word, table of contents, key concepts, original title,

tests & measures, mesh] 9156

43 exp Observation Methods/ or exp Participant Observation/

or participant observation*.mp. 14001

44 exp Hermeneutics/ or hermeneutic*.mp. 7690

45 phenomenol*.mp. or exp Phenomenology/ 46372

46 lived experience*.mp. 15611

47 grounded theory.mp. or exp Grounded Theory/ 16772

48 constant compar*.mp. 4996

49 theoretical sampl*.mp. [mp=title, abstract, heading word,

table of contents, key concepts, original title, tests

& measures, mesh] 597

50 content analy*.mp. 31224

51 thematic analy*.mp. [mp=title, abstract, heading word,

table of contents, key concepts, original title, tests &

measures, mesh] 16314

52 narrative*.mp. [mp=title, abstract, heading word,

table of contents, key concepts, original title, tests &

measures, mesh] 71808

53 unstructured categor*.mp. 5

54 structured categor*.mp. 24

55 unstructured interview*.mp. 1100

56 exp Semi-Structured Interview/ or semi-structured

interview*.mp. 34641

57 maximum variation*.mp. 338

58 snowball.mp. 2410

59 audio.mp. or exp Audiotapes/ 15122

60 tape*.mp. 15593

61 video*.mp. 70380

62 purposive sampl*.mp. [mp=title, abstract,

heading word, table of contents, key concepts, original title,

tests & measures, mesh] 5338

63 action research.mp. or exp Action Research/ 9648

64 exp Focus Group/ or focus group*.mp. 39383

65 28 or 29 or 30 or 31 or 32 or 33 or 34 or 35 or 36 or

37 or 38 or 39 or 40 or 41 or 42 or 43 or 44 or 45 or

46 or 47 or 48 or 49 or 50 or 51 or 52 or 53 or 54 or

55 or 56 or 57 or 58 or 59 or 60 or 61 or 62 or 63 or 64 1381898

66 ((Timing or time or Plan* or Delay* or (late adj3 onset)

or Late-onset or Postpon* or advance*)

adj3 (Motherhood or Mothering or Parenthood or

Parenting or Childbearing or Child-bearing or

(family adj2 formation*) or (maternal adj2 age))).mp.

[mp=title, abstract, heading word, table of contents,

key concepts, original title, tests & measures, mesh] 2011

67 ((Fertility or (biological adj2 clock*) or (Age* adj2 fertil*)

or (fertil* adj2 declin*) or (reproduc* adj2 age*))

adj3 (Consider* or Thought* or Aware* or Cogniti* or

Knowledg* or Attitud* or Reflect* or Perception* or

Perceiv* or Intent* or Opinion* or Concern* or

Expression of concern or Feel* or Emotion* or View*

or Preference* or Value* or Personal value or

Decision making or Acceptab* or Belief* or Believe*

or Decide* or Decision* or Experience*)).mp.

[mp=title, abstract, heading word, table of contents,

key concepts, original title, tests & measures, mesh] 3863

68 27 and 65 and 66 679

69 65 and 67 1430

70 68 or 69 2081

71 limit 70 to (danish or english or norwegian or swedish) 2011

72 limit 71 to ("0100 journal" or "0110 peer-reviewed

journal" or "0120 non-peer-reviewed journal"

or "0130 peer-reviewed status unknown" or "0200 book"

or "0240 authored book" or "0280 edited book" or

"0300 encyclopedia" or "0400 dissertation abstract"

or "0500 electronic collection") 2011

**CINAHL (Ebsco) 28.01.21**

| Thursday, January 28, 2021 3:55:44 AM |  |
| --- | --- |

|  |  | **Limiters/**  **Expanders** |  | **Results** |
| --- | --- | --- | --- | --- |
| S70 | (S1 AND S27 AND S65) OR (S65 AND S66) | Limiters - Publication Type: Abstract, Book, Book Chapter, Book Review, Case Study, Clinical Trial, Commentary, Corrected Article, Doctoral Dissertation, Editorial, Interview, Journal Article, Masters Thesis, Meta Analysis, Meta Synthesis, Randomized Controlled Trial, Research, Review, Systematic Review, Teaching Materials; Language: Danish, English, Norwegian, Swedish |  | 5,143 |
| S69 | (S1 AND S27 AND S65) OR (S65 AND S66) |  |  | 5,514 |
| S68 | S65 AND S66 |  |  | 855 |
| S67 | S1 AND S27 AND S65 |  |  | 4,793 |
| S66 | TI ( ((Fertility OR (MH "Fertility+") OR (biological N2 clock*) OR (MH "Biological Clocks") OR (Age* N2 fertil*) OR (fertil* N2 declin*) OR (reproduc* N2 age*)) N3 (Consider* OR Thought* OR Aware* OR Cognition OR Cogni* OR Knowledge OR Knowledg* OR Attitud* OR Reflect* OR Perception* OR Perceiv* OR Intent* OR Opinion* OR Concern* OR Feel* OR Emotion* OR View* OR Preference* OR Value* OR Valuation* OR Acceptab* OR Belief* OR Believe* OR Decide* OR Decision* OR Decision Making OR Experience*)) ) OR AB ( ((Fertility OR (biological N2 clock*) OR (Age* N2 fertil*) OR (fertil* N2 declin*) OR (reproduc* N2 age*)) N3 (Consider* OR Thought* OR Aware* OR Cognition OR Cogni* OR Knowledge OR Knowledg* OR Attitud* OR Reflect* OR Perception* OR Perceiv* OR Intent* OR Opinion* OR Concern* OR Feel* OR Emotion* OR View* OR Preference* OR Value* OR Valuation* OR Acceptab* OR Belief* OR Believe* OR Decide* OR Decision* OR Decision Making OR Experience*)) ) OR SU ( ((Fertility OR (biological N2 clock*) OR (Age* N2 fertil*) OR (fertil* N2 declin*) OR (reproduc* N2 age*)) N3 (Consider* OR Thought* OR Aware* OR Cognition OR Cogni* OR Knowledge OR Knowledg* OR Attitud* OR Reflect* OR Perception* OR Perceiv* OR Intent* OR Opinion* OR Concern* OR Feel* OR Emotion* OR View* OR Preference* OR Value* OR Valuation* OR Acceptab* OR Belief* OR Believe* OR Decide* OR Decision* OR Decision Making OR Experience*))) |  |  | 2,234 |
| S65 | S28 OR S29 OR S30 OR S31 OR S32 OR S33 OR S34 OR S35 OR S36 OR S37 OR S38 OR S39 OR S40 OR S41 OR S42 OR S43 OR S44 OR S45 OR S46 OR S47 OR S48 OR S49 OR S50 OR S51 OR S52 OR S53 OR S54 OR S55 OR S56 OR S57 OR S58 OR S59 OR S60 OR S61 OR S62 OR S63 OR S64 |  |  | 1,049,493 |
| S64 | (MH "Focus Groups") OR "focus N1 group*" |  |  | 44,819 |
| S63 | (MH "Action Research") OR "action N1 research" |  |  | 6,563 |
| S62 | (MH "Purposive Sample") OR "purposive N1 sampl*" |  |  | 32,331 |
| S61 | (MH "Videorecording+") OR "video*" |  |  | 67,459 |
| S60 | "tape*" |  |  | 13,310 |
| S59 | (MH "Audiorecording") OR "audio" |  |  | 51,601 |
| S58 | (MH "Snowball Sample") OR "snowball" |  |  | 8,206 |
| S57 | "maximum variation*" |  |  | 456 |
| S56 | (MH "Semi-Structured Interview") OR "semi-structured N1 interview*" |  |  | 65,829 |
| S55 | (MH "Unstructured Interview") OR "unstructured N1 interview*" |  |  | 1,643 |
| S54 | (MH "Structured Categories") OR "structured N1 categor*" |  |  | 27 |
| S53 | (MH "Unstructured Categories") OR "unstructured N1 categor*" |  |  | 10 |
| S52 | (MH "Narratives") OR (MH "Storytelling") OR (MH "Life Histories") OR "narrative*" |  |  | 49,058 |
| S51 | (MH "Thematic Analysis") OR "thematic N1 analy*" |  |  | 66,455 |
| S50 | (MH "Content Analysis") OR "content N1 analy*" |  |  | 36,892 |
| S49 | (MH "Theoretical Sample") OR "theoretical N1 sampl*" |  |  | 1,775 |
| S48 | (MH "Constant Comparative Method") OR "constant N1 compar*" |  |  | 7,367 |
| S47 | (MH "Grounded Theory") OR "grounded N1 theory" |  |  | 16,284 |
| S46 | (MH "Life Experiences") OR "lived N1 experience*" |  |  | 27,387 |
| S45 | (MH "Phenomenological Research") OR (MH "Phenomenology") OR "phenomenol*" |  |  | 25,022 |
| S44 | (MH "Phenomenology") OR "hermeneutic*" |  |  | 7,316 |
| S43 | (MH "Participant Observation") OR "participant N1 observation*" |  |  | 5,478 |
| S42 | (MH "observational methods") OR "participant N1 observ*" |  |  | 14,500 |
| S41 | (MH "Field Studies") OR (MH "Fieldwork") OR "field N1 stud*" |  |  | 6,246 |
| S40 | (MH "Field Notes") OR "fieldnote*" |  |  | 8,145 |
| S39 | "field record*" |  |  | 62 |
| S38 | (MH "Field Notes") OR (MH "Field Studies") OR "field N1 note*" |  |  | 11,146 |
| S37 | "etic" |  |  | 136 |
| S36 | "emic" |  |  | 364 |
| S35 | (MH "Ethnonursing Research") OR "ethnonurs*" |  |  | 307 |
| S34 | (MH "Ethnographic Research") OR "ethnog*" |  |  | 13,238 |
| S33 | (MH "Ethnological Research") OR "ethnol*" OR (MH "Ethnology") |  |  | 45,290 |
| S32 | (MH "Qualitative Studies+") OR "qualitative" |  |  | 207,636 |
| S31 | "health care organization" |  |  | 743 |
| S30 | "finding* |  |  | 553,939 |
| S29 | (MH "Semi-Structured Interview") OR (MH "Unstructured Interview") OR (MH "Structured Interview") OR (MH "Interviews+") OR "interview*" |  |  | 332,946 |
| S28 | (MH "Qualitative Studies+") OR "qualitative*" |  |  | 212,151 |
| S27 | S2 OR S3 OR S4 OR S5 OR S6 OR S7 OR S8 OR S9 OR S10 OR S11 OR S12 OR S13 OR S14 OR S15 OR S16 OR S17 OR S18 OR S19 OR S20 OR S21 OR S22 OR S23 OR S24 OR S25 OR S26 |  |  | 2,429,717 |
| S26 | Consider* |  |  | 423,158 |
| S25 | "Thought*" |  |  | 54,483 |
| S24 | (MH "Cognition+") OR "Aware*" |  |  | 167,443 |
| S23 | "Cogniti*" |  |  | 186,050 |
| S22 | (MH "Knowledge+") OR "Knowledg*" |  |  | 252,899 |
| S21 | (MH "Attitude+") OR "Attitud*" |  |  | 493,869 |
| S20 | (MH "Reflection/EI") OR "Reflect*" |  |  | 141,877 |
| S19 | (MH "Perception+") OR "Perception*" |  |  | 188,165 |
| S18 | "Perceiv*" |  |  | 123,619 |
| S17 | (MH "Intention") OR "Intent*" |  |  | 53,705 |
| S16 | "Opinion*" |  |  | 45,267 |
| S15 | "Concern*" |  |  | 193,732 |
| S14 | "Feel*" |  |  | 58,247 |
| S13 | (MH "Emotions+") |  |  | 140,492 |
| S12 | "Emotion*" |  |  | 112,177 |
| S11 | "View*" |  |  | 133,970 |
| S10 | "Preference*" |  |  | 46,758 |
| S9 | "Value*" |  |  | 501,975 |
| S8 | "Valuation*" |  |  | 2,069 |
| S7 | "Acceptab*" |  |  | 51,265 |
| S6 | "Belief*" |  |  | 55,691 |
| S5 | "Believe*" |  |  | 45,202 |
| S4 | "Decide*" |  |  | 14,498 |
| S3 | (MH "Decision Making+") OR "Decision*" |  |  | 237,554 |
| S2 | "Experience*" |  |  | 455,522 |
| S1 | TI ( (Timing OR time OR (MH "Time+") OR Plan* OR Delay* OR (MH "Delayed Onset") OR (late N3 onset) OR Late-onset OR Postpon* OR advance*) N3 (Motherhood OR (MH "Motherhood") OR Mothering OR Parenthood OR (MH "Parenthood+") OR Parenting OR (MH "Parenting") OR Childbearing OR Child-bearing OR (family N2 formation*) OR (maternal N2 age) OR (MH "Maternal Age+") ) ) OR AB ( (Timing OR time OR (MH "Time+") OR Plan* OR Delay* OR (MH "Delayed Onset") OR (late N3 onset) OR Late-onset OR Postpon* OR advance*) N3 (Motherhood OR (MH "Motherhood") OR Mothering OR Parenthood OR (MH "Parenthood+") OR Parenting OR (MH "Parenting") OR Childbearing OR Child-bearing OR (family N2 formation*) OR (maternal N1 age) OR (MH "Maternal Age+")) |  |  | 13,343 |

**SCOPUS 28.01.21**

#7 ( ( ( TITLE-ABS-KEY ( ( ( timing  OR  time  OR  plan*  OR  delay*  OR  ( late  W/3  onset )  OR  late-onset  OR  postpon*  OR  advance* )  W/3  ( motherhood  OR  mothering  OR  parenthood  OR  parenting  OR  childbearing  OR  child-bearing  OR  ( family  W/2  formation* )  OR  ( maternal  W/2  age ) ) ) ) ) )  AND  ( ( TITLE-ABS-KEY ( ( consider*  OR  thought*  OR  aware*  OR  cogniti*  OR  knowledg*  OR  attitud*  OR  reflect*  OR  perception*  OR  perceiv*  OR  intent*  OR  opinion*  OR  concern*  OR  feel*  OR  emotion*  OR  view*  OR  preference*  OR  value*  OR  valuation*  OR  acceptab*  OR  belief*  OR  believe*  OR  decide*  OR  decision*  OR  experience* ) ) ) )  AND  ( ( TITLE-ABS-KEY ( qualitative*  OR  interview*  OR  finding*  OR  ( health  AND  care  AND  organization )  OR  ethnol*  OR  ethnog*  OR  ethnonurs*  OR  emic  OR  etic  OR  ( field  AND NOT  e* )  OR  ( field  AND  record* )  OR  fieldnote*  OR  ( field  AND  stud* )  OR  ( participant  AND  observ* )  OR  ( participant  AND  observation* )  OR  hermeneutic*  OR  phenomenol*  OR  ( lived  AND  experience* )  OR  ( grounded  AND  theory )  OR  ( constant  AND  compar* )  OR  ( theoretical  AND  sampl* )  OR  ( content  AND  analy* )  OR  ( thematic  AND  analy* )  OR  narrative*  OR  ( unstructured  AND  categor* )  OR  ( structured  AND  categor* )  OR  ( unstructured  AND  interview* )  OR  ( semi-structured  AND  interview* )  OR  ( maximum  AND  variation* )  OR  snowball  OR  audio  OR  tape*  OR  video*  OR  ( purposive  AND  sampl* )  OR  ( action  AND  research )  OR  ( focus  AND  group* ) ) ) ) )  OR  ( ( ( TITLE-ABS-KEY ( ( fertility  OR  ( biological  W/2  clock* )  OR  ( age*  W/2  fertil* )  OR  ( fertil*  W/2  declin* )  OR  ( reproduc*  W/2  age* ) )  W/3  ( consider*  OR  thought*  OR  aware*  OR  cogniti*  OR  knowledg*  OR  attitud*  OR  reflect*  OR  perception*  OR  perceiv*  OR  intent*  OR  opinion*  OR  concern*  OR  feel*  OR  emotion*  OR  view*  OR  preference*  OR  value*  OR  valuation*  OR  acceptab*  OR  belief*  OR  believe*  OR  decide*  OR  decision*  OR  experience* ) ) ) )  AND  ( ( TITLE-ABS-KEY ( qualitative*  OR  interview*  OR  finding*  OR  ( health  AND  care  AND  organization )  OR  ethnol*  OR  ethnog*  OR  ethnonurs*  OR  emic  OR  etic  OR  ( field  AND NOT  e* )  OR  ( field  AND  record* )  OR  fieldnote*  OR  ( field  AND  stud* )  OR  ( participant  AND  observ* )  OR  ( participant  AND  observation* )  OR  hermeneutic*  OR  phenomenol*  OR  ( lived  AND  experience* )  OR  ( grounded  AND  theory )  OR  ( constant  AND  compar* )  OR  ( theoretical  AND  sampl* )  OR  ( content  AND  analy* )  OR  ( thematic  AND  analy* )  OR  narrative*  OR  ( unstructured  AND  categor* )  OR  ( structured  AND  categor* )  OR  ( unstructured  AND  interview* )  OR  ( semi-structured  AND  interview* )  OR  ( maximum  AND  variation* )  OR  snowball  OR  audio  OR  tape*  OR  video*  OR  ( purposive  AND  sampl* )  OR  ( action  AND  research )  OR  ( focus  AND  group* ) ) ) ) )  AND  ( LIMIT-TO ( DOCTYPE ,  "ar" )  OR  LIMIT-TO ( DOCTYPE ,  "re" )  OR  LIMIT-TO ( DOCTYPE ,  "ch" )  OR  LIMIT-TO ( DOCTYPE ,  "bk" )  OR  LIMIT-TO ( DOCTYPE ,  "ed" )  OR  LIMIT-TO ( DOCTYPE ,  "cr" ) )  AND  ( LIMIT-TO ( LANGUAGE ,  "English" )  OR  LIMIT-TO ( LANGUAGE ,  "Swedish" ) )

4,426

#6

( ( ( TITLE-ABS-KEY ( ( ( timing  OR  time  OR  plan*  OR  delay*  OR  ( late  W/3  onset )  OR  late-onset  OR  postpon*  OR  advance* )  W/3  ( motherhood  OR  mothering  OR  parenthood  OR  parenting  OR  childbearing  OR  childbearing  OR  ( family  W/2  formation* )  OR  ( maternal  W/2  age ) ) ) ) ) )  AND  ( ( TITLE-ABS-KEY ( ( consider*  OR  thought*  OR  aware*  OR  cogniti*  OR  knowledg*  OR  attitud*  OR  reflect*  OR  perception*  OR  perceiv*  OR  intent*  OR  opinion*  OR  concern*  OR  feel*  OR  emotion*  OR  view*  OR  preference*  OR  value*  OR  valuation*  OR  acceptab*  OR  belief*  OR  believe*  OR  decide*  OR  decision*  OR  experience* ) ) ) )  AND  ( ( TITLE-ABS-KEY ( qualitative*  OR  interview*  OR  finding*  OR  ( health  AND  care  AND  organization )  OR  ethnol*  OR  ethnog*  OR  ethnonurs*  OR  emic  OR  etic  OR  ( field  AND NOT  e* )  OR  ( field  AND  record* )  OR  fieldnote*  OR  ( field  AND  stud* )  OR  ( participant  AND  observ* )  OR  ( participant  AND  observation* )  OR  hermeneutic*  OR  phenomenol*  OR  ( lived  AND  experience* )  OR  ( grounded  AND  theory )  OR  ( constant  AND  compar* )  OR  ( theoretical  AND  sampl* )  OR  ( content  AND  analy* )  OR  ( thematic  AND  analy* )  OR  narrative*  OR  ( unstructured  AND  categor* )  OR  ( structured  AND  categor* )  OR  ( unstructured  AND  interview* )  OR  ( semi-structured  AND  interview* )  OR  ( maximum  AND  variation* )  OR  snowball  OR  audio  OR  tape*  OR  video*  OR  ( purposive  AND  sampl* )  OR  ( action  AND  research )  OR  ( focus  AND  group* ) ) ) ) )  OR  ( ( ( TITLE-ABS-KEY ( ( fertility  OR  ( biological  W/2  clock* )  OR  ( age*  W/2  fertil* )  OR  ( fertil*  W/2  declin* )  OR  ( reproduc*  W/2  age* ) )  W/3  ( consider*  OR  thought*  OR  aware*  OR  cogniti*  OR  knowledg*  OR  attitud*  OR  reflect*  OR  perception*  OR  perceiv*  OR  intent*  OR  opinion*  OR  concern*  OR  feel*  OR  emotion*  OR  view*  OR  preference*  OR  value*  OR  valuation*  OR  acceptab*  OR  belief*  OR  believe*  OR  decide*  OR  decision*  OR  experience* ) ) ) )  AND  ( ( TITLE-ABS-KEY ( qualitative*  OR  interview*  OR  finding*  OR  ( health  AND  care  AND  organization )  OR  ethnol*  OR  ethnog*  OR  ethnonurs*  OR  emic  OR  etic  OR  ( field  AND NOT  e* )  OR  ( field  AND  record* )  OR  fieldnote*  OR  ( field  AND  stud* )  OR  ( participant  AND  observ* )  OR  ( participant  AND  observation* )  OR  hermeneutic*  OR  phenomenol*  OR  ( lived  AND  experience* )  OR  ( grounded  AND  theory )  OR  ( constant  AND  compar* )  OR  ( theoretical  AND  sampl* )  OR  ( content  AND  analy* )  OR  ( thematic  AND  analy* )  OR  narrative*  OR  ( unstructured  AND  categor* )  OR  ( structured  AND  categor* )  OR  ( unstructured  AND  interview* )  OR  ( semi-structured  AND  interview* )  OR  ( maximum  AND  variation* )  OR  snowball  OR  audio  OR  tape*  OR  video*  OR  ( purposive  AND  sampl* )  OR  ( action  AND  research )  OR  ( focus  AND  group* ) ) ) ) )

4,807

#5

( TITLE-ABS-KEY ( qualitative*  OR  interview*  OR  finding*  OR  ( health  AND  care  AND  organization )  OR  ethnol*  OR  ethnog*  OR  ethnonurs*  OR  emic  OR  etic  OR  ( field  AND NOT  e* )  OR  ( field  AND  record* )  OR  fieldnote*  OR  ( field  AND  stud* )  OR  ( participant  AND  observ* )  OR  ( participant  AND  observation* )  OR  hermeneutic*  OR  phenomenol*  OR  ( lived  AND  experience* )  OR  ( grounded  AND  theory )  OR  ( constant  AND  compar* )  OR  ( theoretical  AND  sampl* )  OR  ( content  AND  analy* )  OR  ( thematic  AND  analy* )  OR  narrative*  OR  ( unstructured  AND  categor* )  OR  ( structured  AND  categor* )  OR  ( unstructured  AND  interview* )  OR  ( semi-structured  AND  interview* )  OR  ( maximum  AND  variation* )  OR  snowball  OR  audio  OR  tape*  OR  video*  OR  ( purposive  AND  sampl* )  OR  ( action  AND  research )  OR  ( focus  AND  group* ) ) )

10,940,624

#4

( TITLE-ABS-KEY ( ( fertility  OR  ( biological  W/2  clock* )  OR  ( age*  W/2  fertil* )  OR  ( fertil*  W/2  declin* )  OR  ( reproduc*  W/2  age* ) )  W/3  ( consider*  OR  thought*  OR  aware*  OR  cogniti*  OR  knowledg*  OR  attitud*  OR  reflect*  OR  perception*  OR  perceiv*  OR  intent*  OR  opinion*  OR  concern*  OR  feel*  OR  emotion*  OR  view*  OR  preference*  OR  value*  OR  valuation*  OR  acceptab*  OR  belief*  OR  believe*  OR  decide*  OR  decision*  OR  experience* ) ) )

9,597

#3

( TITLE-ABS-KEY ( qualitative*  OR  interview*  OR  finding*  OR  ( health  AND  care  AND  organization )  OR  ethnol*  OR  ethnog*  OR  ethnonurs*  OR  emic  OR  etic  OR  ( field  AND NOT  e* )  OR  ( field  AND  record* )  OR  fieldnote*  OR  ( field  AND  stud* )  OR  ( participant  AND  observ* )  OR  ( participant  AND  observation* )  OR  hermeneutic*  OR  phenomenol*  OR  ( lived  AND  experience* )  OR  ( grounded  AND  theory )  OR  ( constant  AND  compar* )  OR  ( theoretical  AND  sampl* )  OR  ( content  AND  analy* )  OR  ( thematic  AND  analy* )  OR  narrative*  OR  ( unstructured  AND  categor* )  OR  ( structured  AND  categor* )  OR  ( unstructured  AND  interview* )  OR  ( semi-structured  AND  interview* )  OR  ( maximum  AND  variation* )  OR  snowball  OR  audio  OR  tape*  OR  video*  OR  ( purposive  AND  sampl* )  OR  ( action  AND  research )  OR  ( focus  AND  group* ) ) )

10,940,624

#2

( TITLE-ABS-KEY ( ( consider*  OR  thought*  OR  aware*  OR  cogniti*  OR  knowledg*  OR  attitud*  OR  reflect*  OR  perception*  OR  perceiv*  OR  intent*  OR  opinion*  OR  concern*  OR  feel*  OR  emotion OR  view*  OR  preference*  OR  value*  OR  valuation*  OR  acceptab*  OR  belief*  OR  believe*  OR  decide*  OR  decision*  OR  experience* ) ) )

24,366,741

#1

( TITLE-ABS-KEY ( ( ( timing  OR  time  OR  plan*  OR  delay*  OR  ( late  W/3  onset )  OR  late-onset  OR  postpon*  OR  advance* )  W/3  ( motherhood  OR  mothering  OR  parenthood  OR  parenting  OR  childbearing  OR  child-bearing  OR  ( family  W/2  formation* )  OR  ( maternal  W/2  age ) ) ) ) )

8,582

**PROQUEST DISSERTATION AND THESISES 15.01.21**

S1 TI((Timing OR time OR Plan* OR Delay* OR (late NEAR/3 onset) OR Late-onset OR Postpon* OR advance*) NEAR/3 (Motherhood OR Mothering OR Parenthood OR Parenting OR Childbearing OR Child-bearing OR (family NEAR/2 formation*) OR (maternal NEAR/2 age)))

207

S2 AB((Timing OR time OR Plan* OR Delay* OR (late NEAR/3 onset) OR Late-onset OR Postpon* OR advance*) NEAR/3 (Motherhood OR Mothering OR Parenthood OR Parenting OR Childbearing OR Child-bearing OR (family NEAR/2 formation*) OR (maternal NEAR/2 age)))

1135

S3 TI((Timing OR time OR Plan* OR Delay* OR (late NEAR/3 onset) OR Late-onset OR Postpon* OR advance*) NEAR/3 (Motherhood OR Mothering OR Parenthood OR Parenting OR Childbearing OR Child-bearing OR (family NEAR/2 formation*) OR (maternal NEAR/2 age))) OR AB((Timing OR time OR Plan* OR Delay* OR (late NEAR/3 onset) OR Late-onset OR Postpon* OR advance*) NEAR/3 (Motherhood OR Mothering OR Parenthood OR Parenting OR Childbearing OR Child-bearing OR (family NEAR/2 formation*) OR (maternal NEAR/2 age)))

1217

S4 TI((Consider* OR Thought* OR Aware* OR Cogniti* OR Knowledg* OR Attitud* OR Reflect* OR Perception* OR Perceiv* OR Intent* OR Opinion* OR Concern* OR Feel* OR Emotion* OR View* OR Preference* OR Value* OR Valuation* OR Acceptab* OR Belief* OR Believe* OR Decide* OR Decision* OR Experience*))

396128

S9 TI((Consider* OR Thought* OR Aware* OR Cogniti* OR Knowledg* OR Attitud* OR Reflect* OR Perception* OR Perceiv* OR Intent* OR Opinion* OR Concern* OR Feel* OR Emotion* OR View* OR Preference* OR Value* OR Valuation* OR Acceptab* OR Belief* OR Believe* OR Decide* OR Decision* OR Experience*)) OR AB((Consider* OR Thought* OR Aware* OR Cogniti* OR Knowledg* OR Attitud* OR Reflect* OR Perception* OR Perceiv* OR Intent* OR Opinion* OR Concern* OR Feel* OR Emotion* OR View* OR Preference* OR Value* OR Valuation* OR Acceptab* OR Belief* OR Believe* OR Decide* OR Decision* OR Experience*)) OR (mainsubject.Exact("cognition") OR mainsubject.Exact("knowledge") OR mainsubject.Exact("attitudes") OR mainsubject.Exact("perceptions" OR "perception") OR mainsubject.Exact("opinions") OR mainsubject.Exact("emotions") OR mainsubject.Exact("preferences") ) OR (mainsubject.Exact("values") OR mainsubject.Exact("valuation") OR mainsubject.Exact("acceptability") OR mainsubject.Exact("beliefs") OR mainsubject.Exact("belief & doubt")OR mainsubject.Exact("decision making") )

2247726

S10 TI(qualitative* OR interview* OR finding* OR (health care organization) OR ethnol* OR ethnog* OR ethnonurs* OR emic OR etic OR (field note*) OR (field record*) OR fieldnote* OR (field stud*) OR (participant observ*) OR (participant observation*) OR hermeneutic* OR phenomenol* OR (lived experience*) OR (grounded theory) OR (constant compar*) OR (theoretical sampl*) OR (content analy*) OR (thematic analy*) OR narrative* OR (unstructured categor*) OR (structured categor*) OR (unstructured interview*) OR (semi-structured interview*) OR (maximum variation*) OR snowball OR audio OR tape* OR video* OR (purposive sampl*) OR (action research) OR (focus group*))

82291

S11 AB(qualitative* OR interview* OR finding* OR (health care organization) OR ethnol* OR ethnog* OR ethnonurs* OR emic OR etic OR (field note*) OR (field record*) OR fieldnote* OR (field stud*) OR (participant observ*) OR (participant observation*) OR hermeneutic* OR phenomenol* OR (lived experience*) OR (grounded theory) OR (constant compar*) OR (theoretical sampl*) OR (content analy*) OR (thematic analy*) OR narrative* OR (unstructured categor*) OR (structured categor*) OR (unstructured interview*) OR (semi-structured interview*) OR (maximum variation*) OR snowball OR audio OR tape* OR video* OR (purposive sampl*) OR (action research) OR (focus group*))

1349002

S12 mainsubject.Exact("qualitative research") OR mainsubject.Exact("interviews") OR mainsubject.Exact("ethnology") OR mainsubject.Exact("ethnography") OR mainsubject.Exact("hermeneutics") OR mainsubject.Exact("phenomenology" OR "phenomenological research")

3295

S13 mainsubject.Exact("field study") OR mainsubject.Exact("grounded theory") OR mainsubject.Exact("content analysis") OR mainsubject.Exact("narratives" OR "narrative structure") OR mainsubject.Exact("audio recordings") OR mainsubject.Exact("videotape recordings" OR "video recordings" OR "video" OR "digital video") OR mainsubject.Exact("focus groups" OR "action research")

2695

S14 TI(qualitative* OR interview* OR finding* OR (health care organization) OR ethnol* OR ethnog* OR ethnonurs* OR emic OR etic OR (field note*) OR (field record*) OR fieldnote* OR (field stud*) OR (participant observ*) OR (participant observation*) OR hermeneutic* OR phenomenol* OR (lived experience*) OR (grounded theory) OR (constant compar*) OR (theoretical sampl*) OR (content analy*) OR (thematic analy*) OR narrative* OR (unstructured categor*) OR (structured categor*) OR (unstructured interview*) OR (semi-structured interview*) OR (maximum variation*) OR snowball OR audio OR tape* OR video* OR (purposive sampl*) OR (action research) OR (focus group*)) OR AB(qualitative* OR interview* OR finding* OR (health care organization) OR ethnol* OR ethnog* OR ethnonurs* OR emic OR etic OR (field note*) OR (field record*) OR fieldnote* OR (field stud*) OR (participant observ*) OR (participant observation*) OR hermeneutic* OR phenomenol* OR (lived experience*) OR (grounded theory) OR (constant compar*) OR (theoretical sampl*) OR (content analy*) OR (thematic analy*) OR narrative* OR (unstructured categor*) OR (structured categor*) OR (unstructured interview*) OR (semi-structured interview*) OR (maximum variation*) OR snowball OR audio OR tape* OR video* OR (purposive sampl*) OR (action research) OR (focus group*)) OR (mainsubject.Exact("qualitative research") OR mainsubject.Exact("interviews") OR mainsubject.Exact("ethnology") OR mainsubject.Exact("ethnography") OR mainsubject.Exact("hermeneutics") OR mainsubject.Exact("phenomenology" OR "phenomenological research")) OR (mainsubject.Exact("field study") OR mainsubject.Exact("grounded theory") OR mainsubject.Exact("content analysis") OR mainsubject.Exact("narratives" OR "narrative structure") OR mainsubject.Exact("audio recordings") OR mainsubject.Exact("videotape recordings" OR "video recordings" OR "video" OR "digital video") OR mainsubject.Exact("focus groups" OR "action research"))

1367437

S15 TI((Fertility OR (biological NEAR/2 clock*) OR (Age* NEAR/2 fertil*) OR (fertil* NEAR/2 declin*) OR (reproduc* NEAR/2 age*)) NEAR/3 (Consider* OR Thought* OR Aware* OR Cogniti* OR Knowledg* OR Attitud* OR Reflect* OR Perception* OR Perceiv* OR Intent* OR Opinion* OR Concern* OR Feel* OR Emotion* OR View* OR Preference* OR Value* OR Valuation* OR Acceptab* OR Belief* OR Believe* OR Decide* OR Decision* OR Experience*))

196

S16 AB((Fertility OR (biological NEAR/2 clock*) OR (Age* NEAR/2 fertil*) OR (fertil* NEAR/2 declin*) OR (reproduc* NEAR/2 age*)) NEAR/3 (Consider* OR Thought* OR Aware* OR Cogniti* OR Knowledg* OR Attitud* OR Reflect* OR Perception* OR Perceiv* OR Intent* OR Opinion* OR Concern* OR Feel* OR Emotion* OR View* OR Preference* OR Value* OR Valuation* OR Acceptab* OR Belief* OR Believe* OR Decide* OR Decision* OR Experience*))

1113

S17 TI((Fertility OR (biological NEAR/2 clock*) OR (Age* NEAR/2 fertil*) OR (fertil* NEAR/2 declin*) OR (reproduc* NEAR/2 age*)) NEAR/3 (Consider* OR Thought* OR Aware* OR Cogniti* OR Knowledg* OR Attitud* OR Reflect* OR Perception* OR Perceiv* OR Intent* OR Opinion* OR Concern* OR Feel* OR Emotion* OR View* OR Preference* OR Value* OR Valuation* OR Acceptab* OR Belief* OR Believe* OR Decide* OR Decision* OR Experience*)) OR AB((Fertility OR (biological NEAR/2 clock*) OR (Age* NEAR/2 fertil*) OR (fertil* NEAR/2 declin*) OR (reproduc* NEAR/2 age*)) NEAR/3 (Consider* OR Thought* OR Aware* OR Cogniti* OR Knowledg* OR Attitud* OR Reflect* OR Perception* OR Perceiv* OR Intent* OR Opinion* OR Concern* OR Feel* OR Emotion* OR View* OR Preference* OR Value* OR Valuation* OR Acceptab* OR Belief* OR Believe* OR Decide* OR Decision* OR Experience*))

1193

S18 (TI((Fertility OR (biological NEAR/2 clock*) OR (Age* NEAR/2 fertil*) OR (fertil* NEAR/2 declin*) OR (reproduc* NEAR/2 age*)) NEAR/3 (Consider* OR Thought* OR Aware* OR Cogniti* OR Knowledg* OR Attitud* OR Reflect* OR Perception* OR Perceiv* OR Intent* OR Opinion* OR Concern* OR Feel* OR Emotion* OR View* OR Preference* OR Value* OR Valuation* OR Acceptab* OR Belief* OR Believe* OR Decide* OR Decision* OR Experience*)) OR AB((Fertility OR (biological NEAR/2 clock*) OR (Age* NEAR/2 fertil*) OR (fertil* NEAR/2 declin*) OR (reproduc* NEAR/2 age*)) NEAR/3 (Consider* OR Thought* OR Aware* OR Cogniti* OR Knowledg* OR Attitud* OR Reflect* OR Perception* OR Perceiv* OR Intent* OR Opinion* OR Concern* OR Feel* OR Emotion* OR View* OR Preference* OR Value* OR Valuation* OR Acceptab* OR Belief* OR Believe* OR Decide* OR Decision* OR Experience*))) AND (TI(qualitative* OR interview* OR finding* OR (health care organization) OR ethnol* OR ethnog* OR ethnonurs* OR emic OR etic OR (field note*) OR (field record*) OR fieldnote* OR (field stud*) OR (participant observ*) OR (participant observation*) OR hermeneutic* OR phenomenol* OR (lived experience*) OR (grounded theory) OR (constant compar*) OR (theoretical sampl*) OR (content analy*) OR (thematic analy*) OR narrative* OR (unstructured categor*) OR (structured categor*) OR (unstructured interview*) OR (semi-structured interview*) OR (maximum variation*) OR snowball OR audio OR tape* OR video* OR (purposive sampl*) OR (action research) OR (focus group*)) OR AB(qualitative* OR interview* OR finding* OR (health care organization) OR ethnol* OR ethnog* OR ethnonurs* OR emic OR etic OR (field note*) OR (field record*) OR fieldnote* OR (field stud*) OR (participant observ*) OR (participant observation*) OR hermeneutic* OR phenomenol* OR (lived experience*) OR (grounded theory) OR (constant compar*) OR (theoretical sampl*) OR (content analy*) OR (thematic analy*) OR narrative* OR (unstructured categor*) OR (structured categor*) OR (unstructured interview*) OR (semi-structured interview*) OR (maximum variation*) OR snowball OR audio OR tape* OR video* OR (purposive sampl*) OR (action research) OR (focus group*)) OR (mainsubject.Exact("qualitative research") OR mainsubject.Exact("interviews") OR mainsubject.Exact("ethnology") OR mainsubject.Exact("ethnography") OR mainsubject.Exact("hermeneutics") OR mainsubject.Exact("phenomenology" OR "phenomenological research")) OR (mainsubject.Exact("field study") OR mainsubject.Exact("grounded theory") OR mainsubject.Exact("content analysis") OR mainsubject.Exact("narratives" OR "narrative structure") OR mainsubject.Exact("audio recordings") OR mainsubject.Exact("videotape recordings" OR "video recordings" OR "video" OR "digital video") OR mainsubject.Exact("focus groups" OR "action research")))

629

S19 (TI((Timing OR time OR Plan* OR Delay* OR (late NEAR/3 onset) OR Late-onset OR Postpon* OR advance*) NEAR/3 (Motherhood OR Mothering OR Parenthood OR Parenting OR Childbearing OR Child-bearing OR (family NEAR/2 formation*) OR (maternal NEAR/2 age))) OR AB((Timing OR time OR Plan* OR Delay* OR (late NEAR/3 onset) OR Late-onset OR Postpon* OR advance*) NEAR/3 (Motherhood OR Mothering OR Parenthood OR Parenting OR Childbearing OR Child-bearing OR (family NEAR/2 formation*) OR (maternal NEAR/2 age)))) AND (TI((Consider* OR Thought* OR Aware* OR Cogniti* OR Knowledg* OR Attitud* OR Reflect* OR Perception* OR Perceiv* OR Intent* OR Opinion* OR Concern* OR Feel* OR Emotion* OR View* OR Preference* OR Value* OR Valuation* OR Acceptab* OR Belief* OR Believe* OR Decide* OR Decision* OR Experience*)) OR AB((Consider* OR Thought* OR Aware* OR Cogniti* OR Knowledg* OR Attitud* OR Reflect* OR Perception* OR Perceiv* OR Intent* OR Opinion* OR Concern* OR Feel* OR Emotion* OR View* OR Preference* OR Value* OR Valuation* OR Acceptab* OR Belief* OR Believe* OR Decide* OR Decision* OR Experience*)) OR (mainsubject.Exact("cognition") OR mainsubject.Exact("knowledge") OR mainsubject.Exact("attitudes") OR mainsubject.Exact("perceptions" OR "perception") OR mainsubject.Exact("opinions") OR mainsubject.Exact("emotions") OR mainsubject.Exact("preferences") ) OR (mainsubject.Exact("values") OR mainsubject.Exact("valuation") OR mainsubject.Exact("acceptability") OR mainsubject.Exact("beliefs") OR mainsubject.Exact("belief & doubt")OR mainsubject.Exact("decision making") )) AND (TI(qualitative* OR interview* OR finding* OR (health care organization) OR ethnol* OR ethnog* OR ethnonurs* OR emic OR etic OR (field note*) OR (field record*) OR fieldnote* OR (field stud*) OR (participant observ*) OR (participant observation*) OR hermeneutic* OR phenomenol* OR (lived experience*) OR (grounded theory) OR (constant compar*) OR (theoretical sampl*) OR (content analy*) OR (thematic analy*) OR narrative* OR (unstructured categor*) OR (structured categor*) OR (unstructured interview*) OR (semi-structured interview*) OR (maximum variation*) OR snowball OR audio OR tape* OR video* OR (purposive sampl*) OR (action research) OR (focus group*)) OR AB(qualitative* OR interview* OR finding* OR (health care organization) OR ethnol* OR ethnog* OR ethnonurs* OR emic OR etic OR (field note*) OR (field record*) OR fieldnote* OR (field stud*) OR (participant observ*) OR (participant observation*) OR hermeneutic* OR phenomenol* OR (lived experience*) OR (grounded theory) OR (constant compar*) OR (theoretical sampl*) OR (content analy*) OR (thematic analy*) OR narrative* OR (unstructured categor*) OR (structured categor*) OR (unstructured interview*) OR (semi-structured interview*) OR (maximum variation*) OR snowball OR audio OR tape* OR video* OR (purposive sampl*) OR (action research) OR (focus group*)) OR (mainsubject.Exact("qualitative research") OR mainsubject.Exact("interviews") OR mainsubject.Exact("ethnology") OR mainsubject.Exact("ethnography") OR mainsubject.Exact("hermeneutics") OR mainsubject.Exact("phenomenology" OR "phenomenological research")) OR (mainsubject.Exact("field study") OR mainsubject.Exact("grounded theory") OR mainsubject.Exact("content analysis") OR mainsubject.Exact("narratives" OR "narrative structure") OR mainsubject.Exact("audio recordings") OR mainsubject.Exact("videotape recordings" OR "video recordings" OR "video" OR "digital video") OR mainsubject.Exact("focus groups" OR "action research")))

686

S20 ((TI((Fertility OR (biological NEAR/2 clock*) OR (Age* NEAR/2 fertil*) OR (fertil* NEAR/2 declin*) OR (reproduc* NEAR/2 age*)) NEAR/3 (Consider* OR Thought* OR Aware* OR Cogniti* OR Knowledg* OR Attitud* OR Reflect* OR Perception* OR Perceiv* OR Intent* OR Opinion* OR Concern* OR Feel* OR Emotion* OR View* OR Preference* OR Value* OR Valuation* OR Acceptab* OR Belief* OR Believe* OR Decide* OR Decision* OR Experience*)) OR AB((Fertility OR (biological NEAR/2 clock*) OR (Age* NEAR/2 fertil*) OR (fertil* NEAR/2 declin*) OR (reproduc* NEAR/2 age*)) NEAR/3 (Consider* OR Thought* OR Aware* OR Cogniti* OR Knowledg* OR Attitud* OR Reflect* OR Perception* OR Perceiv* OR Intent* OR Opinion* OR Concern* OR Feel* OR Emotion* OR View* OR Preference* OR Value* OR Valuation* OR Acceptab* OR Belief* OR Believe* OR Decide* OR Decision* OR Experience*))) AND (TI(qualitative* OR interview* OR finding* OR (health care organization) OR ethnol* OR ethnog* OR ethnonurs* OR emic OR etic OR (field note*) OR (field record*) OR fieldnote* OR (field stud*) OR (participant observ*) OR (participant observation*) OR hermeneutic* OR phenomenol* OR (lived experience*) OR (grounded theory) OR (constant compar*) OR (theoretical sampl*) OR (content analy*) OR (thematic analy*) OR narrative* OR (unstructured categor*) OR (structured categor*) OR (unstructured interview*) OR (semi-structured interview*) OR (maximum variation*) OR snowball OR audio OR tape* OR video* OR (purposive sampl*) OR (action research) OR (focus group*)) OR AB(qualitative* OR interview* OR finding* OR (health care organization) OR ethnol* OR ethnog* OR ethnonurs* OR emic OR etic OR (field note*) OR (field record*) OR fieldnote* OR (field stud*) OR (participant observ*) OR (participant observation*) OR hermeneutic* OR phenomenol* OR (lived experience*) OR (grounded theory) OR (constant compar*) OR (theoretical sampl*) OR (content analy*) OR (thematic analy*) OR narrative* OR (unstructured categor*) OR (structured categor*) OR (unstructured interview*) OR (semi-structured interview*) OR (maximum variation*) OR snowball OR audio OR tape* OR video* OR (purposive sampl*) OR (action research) OR (focus group*)) OR (mainsubject.Exact("qualitative research") OR mainsubject.Exact("interviews") OR mainsubject.Exact("ethnology") OR mainsubject.Exact("ethnography") OR mainsubject.Exact("hermeneutics") OR mainsubject.Exact("phenomenology" OR "phenomenological research")) OR (mainsubject.Exact("field study") OR mainsubject.Exact("grounded theory") OR mainsubject.Exact("content analysis") OR mainsubject.Exact("narratives" OR "narrative structure") OR mainsubject.Exact("audio recordings") OR mainsubject.Exact("videotape recordings" OR "video recordings" OR "video" OR "digital video") OR mainsubject.Exact("focus groups" OR "action research")))) OR ((TI((Timing OR time OR Plan* OR Delay* OR (late NEAR/3 onset) OR Late-onset OR Postpon* OR advance*) NEAR/3 (Motherhood OR Mothering OR Parenthood OR Parenting OR Childbearing OR Child-bearing OR (family NEAR/2 formation*) OR (maternal NEAR/2 age))) OR AB((Timing OR time OR Plan* OR Delay* OR (late NEAR/3 onset) OR Late-onset OR Postpon* OR advance*) NEAR/3 (Motherhood OR Mothering OR Parenthood OR Parenting OR Childbearing OR Child-bearing OR (family NEAR/2 formation*) OR (maternal NEAR/2 age)))) AND (TI((Consider* OR Thought* OR Aware* OR Cogniti* OR Knowledg* OR Attitud* OR Reflect* OR Perception* OR Perceiv* OR Intent* OR Opinion* OR Concern* OR Feel* OR Emotion* OR View* OR Preference* OR Value* OR Valuation* OR Acceptab* OR Belief* OR Believe* OR Decide* OR Decision* OR Experience*)) OR AB((Consider* OR Thought* OR Aware* OR Cogniti* OR Knowledg* OR Attitud* OR Reflect* OR Perception* OR Perceiv* OR Intent* OR Opinion* OR Concern* OR Feel* OR Emotion* OR View* OR Preference* OR Value* OR Valuation* OR Acceptab* OR Belief* OR Believe* OR Decide* OR Decision* OR Experience*)) OR (mainsubject.Exact("cognition") OR mainsubject.Exact("knowledge") OR mainsubject.Exact("attitudes") OR mainsubject.Exact("perceptions" OR "perception") OR mainsubject.Exact("opinions") OR mainsubject.Exact("emotions") OR mainsubject.Exact("preferences") ) OR (mainsubject.Exact("values") OR mainsubject.Exact("valuation") OR mainsubject.Exact("acceptability") OR mainsubject.Exact("beliefs") OR mainsubject.Exact("belief & doubt")OR mainsubject.Exact("decision making") )) AND (TI(qualitative* OR interview* OR finding* OR (health care organization) OR ethnol* OR ethnog* OR ethnonurs* OR emic OR etic OR (field note*) OR (field record*) OR fieldnote* OR (field stud*) OR (participant observ*) OR (participant observation*) OR hermeneutic* OR phenomenol* OR (lived experience*) OR (grounded theory) OR (constant compar*) OR (theoretical sampl*) OR (content analy*) OR (thematic analy*) OR narrative* OR (unstructured categor*) OR (structured categor*) OR (unstructured interview*) OR (semi-structured interview*) OR (maximum variation*) OR snowball OR audio OR tape* OR video* OR (purposive sampl*) OR (action research) OR (focus group*)) OR AB(qualitative* OR interview* OR finding* OR (health care organization) OR ethnol* OR ethnog* OR ethnonurs* OR emic OR etic OR (field note*) OR (field record*) OR fieldnote* OR (field stud*) OR (participant observ*) OR (participant observation*) OR hermeneutic* OR phenomenol* OR (lived experience*) OR (grounded theory) OR (constant compar*) OR (theoretical sampl*) OR (content analy*) OR (thematic analy*) OR narrative* OR (unstructured categor*) OR (structured categor*) OR (unstructured interview*) OR (semi-structured interview*) OR (maximum variation*) OR snowball OR audio OR tape* OR video* OR (purposive sampl*) OR (action research) OR (focus group*)) OR (mainsubject.Exact("qualitative research") OR mainsubject.Exact("interviews") OR mainsubject.Exact("ethnology") OR mainsubject.Exact("ethnography") OR mainsubject.Exact("hermeneutics") OR mainsubject.Exact("phenomenology" OR "phenomenological research")) OR (mainsubject.Exact("field study") OR mainsubject.Exact("grounded theory") OR mainsubject.Exact("content analysis") OR mainsubject.Exact("narratives" OR "narrative structure") OR mainsubject.Exact("audio recordings") OR mainsubject.Exact("videotape recordings" OR "video recordings" OR "video" OR "digital video") OR mainsubject.Exact("focus groups" OR "action research"))))

1282

S21 (((TI((Fertility OR (biological NEAR/2 clock*) OR (Age* NEAR/2 fertil*) OR (fertil* NEAR/2 declin*) OR (reproduc* NEAR/2 age*)) NEAR/3 (Consider* OR Thought* OR Aware* OR Cogniti* OR Knowledg* OR Attitud* OR Reflect* OR Perception* OR Perceiv* OR Intent* OR Opinion* OR Concern* OR Feel* OR Emotion* OR View* OR Preference* OR Value* OR Valuation* OR Acceptab* OR Belief* OR Believe* OR Decide* OR Decision* OR Experience*)) OR AB((Fertility OR (biological NEAR/2 clock*) OR (Age* NEAR/2 fertil*) OR (fertil* NEAR/2 declin*) OR (reproduc* NEAR/2 age*)) NEAR/3 (Consider* OR Thought* OR Aware* OR Cogniti* OR Knowledg* OR Attitud* OR Reflect* OR Perception* OR Perceiv* OR Intent* OR Opinion* OR Concern* OR Feel* OR Emotion* OR View* OR Preference* OR Value* OR Valuation* OR Acceptab* OR Belief* OR Believe* OR Decide* OR Decision* OR Experience*))) AND (TI(qualitative* OR interview* OR finding* OR (health care organization) OR ethnol* OR ethnog* OR ethnonurs* OR emic OR etic OR (field note*) OR (field record*) OR fieldnote* OR (field stud*) OR (participant observ*) OR (participant observation*) OR hermeneutic* OR phenomenol* OR (lived experience*) OR (grounded theory) OR (constant compar*) OR (theoretical sampl*) OR (content analy*) OR (thematic analy*) OR narrative* OR (unstructured categor*) OR (structured categor*) OR (unstructured interview*) OR (semi-structured interview*) OR (maximum variation*) OR snowball OR audio OR tape* OR video* OR (purposive sampl*) OR (action research) OR (focus group*)) OR AB(qualitative* OR interview* OR finding* OR (health care organization) OR ethnol* OR ethnog* OR ethnonurs* OR emic OR etic OR (field note*) OR (field record*) OR fieldnote* OR (field stud*) OR (participant observ*) OR (participant observation*) OR hermeneutic* OR phenomenol* OR (lived experience*) OR (grounded theory) OR (constant compar*) OR (theoretical sampl*) OR (content analy*) OR (thematic analy*) OR narrative* OR (unstructured categor*) OR (structured categor*) OR (unstructured interview*) OR (semi-structured interview*) OR (maximum variation*) OR snowball OR audio OR tape* OR video* OR (purposive sampl*) OR (action research) OR (focus group*)) OR (mainsubject.Exact("qualitative research") OR mainsubject.Exact("interviews") OR mainsubject.Exact("ethnology") OR mainsubject.Exact("ethnography") OR mainsubject.Exact("hermeneutics") OR mainsubject.Exact("phenomenology" OR "phenomenological research")) OR (mainsubject.Exact("field study") OR mainsubject.Exact("grounded theory") OR mainsubject.Exact("content analysis") OR mainsubject.Exact("narratives" OR "narrative structure") OR mainsubject.Exact("audio recordings") OR mainsubject.Exact("videotape recordings" OR "video recordings" OR "video" OR "digital video") OR mainsubject.Exact("focus groups" OR "action research")))) OR ((TI((Timing OR time OR Plan* OR Delay* OR (late NEAR/3 onset) OR Late-onset OR Postpon* OR advance*) NEAR/3 (Motherhood OR Mothering OR Parenthood OR Parenting OR Childbearing OR Child-bearing OR (family NEAR/2 formation*) OR (maternal NEAR/2 age))) OR AB((Timing OR time OR Plan* OR Delay* OR (late NEAR/3 onset) OR Late-onset OR Postpon* OR advance*) NEAR/3 (Motherhood OR Mothering OR Parenthood OR Parenting OR Childbearing OR Child-bearing OR (family NEAR/2 formation*) OR (maternal NEAR/2 age)))) AND (TI((Consider* OR Thought* OR Aware* OR Cogniti* OR Knowledg* OR Attitud* OR Reflect* OR Perception* OR Perceiv* OR Intent* OR Opinion* OR Concern* OR Feel* OR Emotion* OR View* OR Preference* OR Value* OR Valuation* OR Acceptab* OR Belief* OR Believe* OR Decide* OR Decision* OR Experience*)) OR AB((Consider* OR Thought* OR Aware* OR Cogniti* OR Knowledg* OR Attitud* OR Reflect* OR Perception* OR Perceiv* OR Intent* OR Opinion* OR Concern* OR Feel* OR Emotion* OR View* OR Preference* OR Value* OR Valuation* OR Acceptab* OR Belief* OR Believe* OR Decide* OR Decision* OR Experience*)) OR (mainsubject.Exact("cognition") OR mainsubject.Exact("knowledge") OR mainsubject.Exact("attitudes") OR mainsubject.Exact("perceptions" OR "perception") OR mainsubject.Exact("opinions") OR mainsubject.Exact("emotions") OR mainsubject.Exact("preferences") ) OR (mainsubject.Exact("values") OR mainsubject.Exact("valuation") OR mainsubject.Exact("acceptability") OR mainsubject.Exact("beliefs") OR mainsubject.Exact("belief & doubt")OR mainsubject.Exact("decision making") )) AND (TI(qualitative* OR interview* OR finding* OR (health care organization) OR ethnol* OR ethnog* OR ethnonurs* OR emic OR etic OR (field note*) OR (field record*) OR fieldnote* OR (field stud*) OR (participant observ*) OR (participant observation*) OR hermeneutic* OR phenomenol* OR (lived experience*) OR (grounded theory) OR (constant compar*) OR (theoretical sampl*) OR (content analy*) OR (thematic analy*) OR narrative* OR (unstructured categor*) OR (structured categor*) OR (unstructured interview*) OR (semi-structured interview*) OR (maximum variation*) OR snowball OR audio OR tape* OR video* OR (purposive sampl*) OR (action research) OR (focus group*)) OR AB(qualitative* OR interview* OR finding* OR (health care organization) OR ethnol* OR ethnog* OR ethnonurs* OR emic OR etic OR (field note*) OR (field record*) OR fieldnote* OR (field stud*) OR (participant observ*) OR (participant observation*) OR hermeneutic* OR phenomenol* OR (lived experience*) OR (grounded theory) OR (constant compar*) OR (theoretical sampl*) OR (content analy*) OR (thematic analy*) OR narrative* OR (unstructured categor*) OR (structured categor*) OR (unstructured interview*) OR (semi-structured interview*) OR (maximum variation*) OR snowball OR audio OR tape* OR video* OR (purposive sampl*) OR (action research) OR (focus group*)) OR (mainsubject.Exact("qualitative research") OR mainsubject.Exact("interviews") OR mainsubject.Exact("ethnology") OR mainsubject.Exact("ethnography") OR mainsubject.Exact("hermeneutics") OR mainsubject.Exact("phenomenology" OR "phenomenological research")) OR (mainsubject.Exact("field study") OR mainsubject.Exact("grounded theory") OR mainsubject.Exact("content analysis") OR mainsubject.Exact("narratives" OR "narrative structure") OR mainsubject.Exact("audio recordings") OR mainsubject.Exact("videotape recordings" OR "video recordings" OR "video" OR "digital video") OR mainsubject.Exact("focus groups" OR "action research"))))) AND la.exact("ENG")

1237

**UPDATED SEARCH 13.01.22**

**EMBASE (Ovid) 13.01.22**

Embase Classic+Embase <1947 to 2022 January 12>

1 Consider*.mp. 3199858

2 Thought*.mp. 408264

3 Aware*.mp. or exp awareness/ 402112

4 exp cognition/ or Cogniti*.mp. 3034392

5 exp knowledge/ or Knowledg*.mp. 1146072

6 exp attitude/ or Attitud*.mp. 974577

7 Reflect*.mp. 837867

8 Perception*.mp. or exp perception/ 731222

9 Perceiv*.mp. 322256

10 Intent*.mp. 196021

11 Opinion*.mp. 186641

12 exp "expression of concern"/ or Concern*.mp. 971621

13 Feel*.mp. 166079

14 emotion/ or Emotion*.mp. 379038

15 View*.mp. 706577

16 Preference*.mp. 237490

17 exp personal value/ or Value*.mp. 3237707

18 decision making/ or Valuation*.mp. 265838

19 Acceptab*.mp. 285021

20 Belief*.mp. 123405

21 Believe*.mp. 328710

22 Decide*.mp. 105783

23 Decision*.mp. 833291

24 Experience*.mp. 1791922

25 1 or 2 or 3 or 4 or 5 or 6 or 7 or 8 or 9 or 10 or 11 or 12 or 13 or

14 or 15 or 16 or 17 or 18 or 19 or 20 or 21 or 22 or 23 or 24 13232358

26 qualitative*.mp. or exp qualitative research/ 442534

27 exp interview/ or exp semi structured interview/ or

interview*.mp. 587003

28 finding*.mp. 3500178

29 health care organization.mp. or exp health care organization/ 1970476

30 exp ethnology/ or ethnol*.mp. 82320

31 exp ethnographic research/ or exp ethnography/ or ethnog*.mp. 14883

32 exp ethnonursing research/ or ethnonurs*.mp. 128

33 emic.mp. 816

34 etic.mp. 447

35 field note*.mp. 3150

36 field record*.mp. 781

37 fieldnote*.mp. 213

38 exp field study/ or field stud*.mp. 24150

39 exp participant observation/ or participant observ*.mp. 10370

40 participant observation*.mp. or participant observation/ 10127

41 exp hermeneutics/ or hermeneutic*.mp. 4768

42 exp phenomenology/ or phenomenol*.mp. 37710

43 lived experience*.mp. 9989

44 grounded theory.mp. or exp grounded theory/ 16940

45 exp constant comparative method/ or constant compar*.mp. 7143

46 exp theoretical sample/ or theoretical sampl*.mp. 1066

47 exp content analysis/ or content analy*.mp. 45607

48 exp thematic analysis/ or thematic analy*.mp. 38532

49 exp narrative/ or narrative*.mp. 64647

50 unstructured categor*.mp. 3

51 structured categor*.mp. 28

52 unstructured interview*.mp. 1179

53 exp semi structured interview/ or semi-structured interview*.mp. 68934

54 maximum variation*.mp. 1522

55 exp snowball sample/ or snowball.mp. 4704

56 exp audio recording/ or audio.mp. 30584

57 exp tape recorder/ or tape*.mp. 86260

58 video*.mp. 237189

59 exp purposive sample/ or purposive sampl*.mp. 13933

60 action research.mp. or exp action research/ 6185

61 focus group*.mp. 68629

62 26 or 27 or 28 or 29 or 30 or 31 or 32 or 33 or 34 or 35 or 36 or

37 or 38 or 39 or 40 or 41 or 42 or 43 or 44 or 45 or 46 or 47 or

48 or 49 or 50 or 51 or 52 or 53 or 54 or 55 or 56 or 57 or 58 or

59 or 60 or 61 6345012

63 ((Timing or time or Plan* or Delay* or (late adj3 onset) or

Late-onset or Postpon* or advance*) adj3 (Motherhood or

Mothering or Parenthood or Parenting or Childbearing or

Child-bearing or (family adj2 formation*) or

(maternal adj2 age))).mp. [mp=title, abstract, heading word,

drug trade name, original title, device manufacturer,

drug manufacturer, device trade name, keyword heading word,

floating subheading word, candidate term word] 8201

64 ((Fertility or (biological adj2 clock*) or (Age* adj2 fertil*) or

(fertil* adj2 declin*) or (reproduc* adj2 age*)) adj3

(Consider* or Thought* or Aware* or Cogniti* or Knowledg*

or Attitud* or Reflect* or Perception* or Perceiv* or Intent*

or Opinion* or Concern* or Expression of concern or Feel*

or Emotion* or View* or Preference* or Value* or

Personal value or Decision making or Acceptab* or Belief*

or Believe* or Decide* or Decision* or Experience*)).mp.

[mp=title, abstract, heading word, drug trade name, original title,

device manufacturer, drug manufacturer, device trade name,

keyword heading word, floating subheading word,

candidate term word] 6302

65 25 and 62 and 63 1616

66 62 and 64 2393

67 65 or 66 3895

68 limit 67 to (danish or english or norwegian or swedish) 3800

69 limit 68 to (article or article in press or books or chapter or

editorial or "review") 2528

70 limit 69 to yr="2021 -Current" 279

**MEDLINE (Ovid) 13.01.22**

Ovid MEDLINE(R) ALL <1946 to January 12, 2022>

1 Consider*.mp. 2286526

2 Thought*.mp. 290023

3 Aware*.mp. 271908

4 exp Cognition/ or Cogniti*.mp. 571662

5 exp Knowledge/ or Knowledg*.mp. 883558

6 Attitud*.mp. 458583

7 Reflect*.mp. 657389

8 exp Perception/ or Perception*.mp. 672251

9 Perceiv*.mp. [mp=title, abstract, original title, name of

substance word, subject heading word, floating sub-heading

word, keyword heading word, organism supplementary concept

word, protocol supplementary concept word, rare disease

supplementary concept word, unique identifier, synonyms] 261561

10 Intent*.mp. 139463

11 Opinion*.mp. 133902

12 Concern*.mp. 691837

13 Feel*.mp. 106960

14 exp Emotions/ or Emotion*.mp. 431498

15 View*.mp. 512686

16 Preference*.mp. 188128

17 Value*.mp. 2393403

18 Valuation*.mp. 7944

19 Acceptab*.mp. [mp=title, abstract, original title, name of

substance word, subject heading word, floating sub-heading

word, keyword heading word, organism supplementary concept

word, protocol supplementary concept word, rare disease

supplementary concept word, unique identifier, synonyms] 203691

20 Belief*.mp. 93774

21 Believe*.mp. 235153

22 Decide*.mp. or exp Decision Making/ 275238

23 Decision*.mp. or Decision Making/ 516917

24 Experience*.mp. 1211496

25 1 or 2 or 3 or 4 or 5 or 6 or 7 or 8 or 9 or 10 or 11 or 12 or 13 or

14 or 15 or 16 or 17 or 18 or 19 or 20 or 21 or 22 or 23 or 24 9257644

26 exp Qualitative Research/ or qualitative*.mp. 341815

27 interview*.mp. or exp Interview/ 437729

28 finding*.mp. 2633503

29 health care organization.mp. 1378

30 exp Qualitative Research/ or qualitative.mp. 293460

31 exp Ethnology/ or ethnol*.mp. 173501

32 ethnog*.mp. [mp=title, abstract, original title,

name of substance word, subject heading word,

floating sub-heading word, keyword heading word,

organism supplementary concept word,

protocol supplementary concept word,

rare disease supplementary concept word,

unique identifier, synonyms] 12695

33 ethnonurs*.mp. [mp=title, abstract, original title,

name of substance word, subject heading word,

floating sub-heading word, keyword heading word,

organism supplementary concept word,

protocol supplementary concept word,

rare disease supplementary concept word,

unique identifier, synonyms] 124

34 emic.mp. 663

35 etic.mp. 257

36 field note*.mp. 2612

37 field record*.mp. 658

38 fieldnote*.mp. 168

39 field stud*.mp. 16885

40 participant observ*.mp. 5064

41 participant observation*.mp. 4875

42 hermeneutic*.mp. or exp Hermeneutics/ 4241

43 phenomenol*.mp. 30129

44 lived experience*.mp. 8456

45 grounded theory.mp. or exp Grounded Theory/ 13497

46 constant compar*.mp. 5726

47 theoretical sampl*.mp. 805

48 content analy*.mp. 37730

49 thematic analy*.mp. [mp=title, abstract, original title,

name of substance word, subject heading word,

floating sub-heading word, keyword heading word,

organism supplementary concept word,

protocol supplementary concept word,

rare disease supplementary concept word,

unique identifier, synonyms] 28899

50 narrative*.mp. 63891

51 unstructured categor*.mp. 3

52 structured categor*.mp. 21

53 unstructured interview*.mp. 854

54 semi-structured interview*.mp. 39292

55 maximum variation*.mp. 1209

56 snowball.mp. 3751

57 audio.mp. 55136

58 tape*.mp. 59184

59 video*.mp. 197191

60 purposive sampl*.mp. [mp=title, abstract, original title,

name of substance word, subject heading word,

floating sub-heading word, keyword heading word,

organism supplementary concept word,

protocol supplementary concept word,

rare disease supplementary concept word,

unique identifier, synonyms] 9773

61 action research.mp. 4925

62 focus group*.mp. or exp Focus Groups/ 60775

63 26 or 27 or 28 or 29 or 30 or 31 or 32 or 33 or 34 or 35 or 36

or 37 or 38 or 39 or 40 or 41 or 42 or 43 or 44 or 45 or 46 or 47

or 48 or 49 or 50 or 51 or 52 or 53 or 54 or 55 or 56 or 57 or 58

or 59 or 60 or 61 or 62 3621494

64 ((Timing or time or Plan* or Delay* or (late adj3 onset)

or Late-onset or Postpon* or advance*)

adj3 (Motherhood or Mothering or Parenthood or Parenting

or Childbearing or Child-bearing or (family adj2 formation*)

or (maternal adj2 age))).mp. [mp=title, abstract, original title,

name of substance word, subject heading word,

floating sub-heading word, keyword heading word,

organism supplementary concept word,

protocol supplementary concept word,

rare disease supplementary concept word,

unique identifier, synonyms] 6316

65 ((Fertility or (biological adj2 clock*) or (Age* adj2 fertil*) or

(fertil* adj2 declin*) or (reproduc* adj2 age*))

adj3 (Consider* or Thought* or Aware* or Cogniti* or Knowledg*

or Attitud* or Reflect* or Perception* or Perceiv* or Intent*

or Opinion* or Concern* or Expression of concern or Feel*

or Emotion* or View* or Preference* or Value* or Personal value

or Decision making or Acceptab* or Belief* or Believe* or Decide*

or Decision* or Experience*)).mp. [mp=title, abstract, original title,

name of substance word, subject heading word,

floating sub-heading word, keyword heading word,

organism supplementary concept word,

protocol supplementary concept word,

rare disease supplementary concept word,

unique identifier, synonyms] 5100

66 25 and 63 and 64 1009

67 63 and 65 1602

68 66 or 67 2546

69 limit 68 to (danish or english or norwegian or swedish) 2476

70 limit 69 to (adaptive clinical trial or case reports or classical article

or clinical study or clinical trial, all or clinical trial,

phase i or clinical trial, phase ii or clinical trial,

phase iii or clinical trial, phase iv or clinical trial or comment

or comparative study or controlled clinical trial

or "corrected and republished article" or duplicate publication

or editorial or evaluation study or government publication

or guideline or interactive tutorial or interview

or introductory journal article or journal article or lecture

or meta analysis or multicenter study or observational study

or personal narrative or preprint or randomized controlled trial

or "review" or "systematic review") 2475

71 limit 70 to yr="2021 -Current" 274

**PSYCINFO (Ovid) 13.01.22**

APA 13.01.22 PsycInfo <1806 to January Week 1 2022>

1 Consider*.mp. 587261

2 Thought*.mp. 158833

3 exp Awareness/ or Aware*.mp. 224542

4 exp Cognitions/ or Cogniti*.mp. 728054

5 Knowledg*.mp. 358836

6 Attitud*.mp. 515309

7 Reflect*.mp. 264722

8 Perception*.mp. 568549

9 Perceiv*.mp. 287952

10 exp Intention/ or Intent*.mp. 108148

11 exp Attitudes/ or Opinion*.mp. 449717

12 Concern*.mp. 323404

13 Expression of concern.mp. 133

14 exp Emotions/ or Feel*.mp. 512562

15 Emotion*.mp. 452591

16 View*.mp. 326792

17 Preference*.mp. 122668

18 Value*.mp. or exp Values/ 392323

19 Personal value.mp. or exp Personal Values/ 4977

20 Decision making.mp. or exp Decision Making/ 182310

21 Acceptab*.mp. [mp=title, abstract, heading word,

table of contents, key concepts, original title, tests & measures,

mesh word] 45181

22 Belief*.mp. 151592

23 Believe*.mp. 89725

24 exp Decision Making/ or Decide*.mp. 152090

25 Decision*.mp. 248108

26 Experience*.mp. 739782

27 1 or 2 or 3 or 4 or 5 or 6 or 7 or 8 or 9 or 10 or 11 or 12 or 13

or 14 or 15 or 16 or 17 or 18 or 19 or 20 or 21 or 22 or 23 or 24

or 25 or 26 3525134

28 exp Qualitative Methods/ or qualitative*.mp. 214628

29 exp Focus Group Interview/ or interview*.mp.

or exp Semi-Structured Interview/ 447442

30 finding*.mp. 895500

31 health care organization.mp. 464

32 qualitative.mp. 198767

33 ethnol*.mp. 3572

34 ethnog*.mp. [mp=title, abstract, heading word,

table of contents, key concepts, original title, tests & measures,

mesh word] 32100

35 ethnonurs*.mp. [mp=title, abstract, heading word,

table of contents, key concepts, original title, tests & measures,

mesh word] 66

36 emic.mp. 1274

37 etic.mp. 717

38 field note*.mp. 4473

39 field record*.mp. 172

40 fieldnote*.mp. 408

41 field stud*.mp. 8958

42 participant observ*.mp. [mp=title, abstract, heading word,

table of contents, key concepts, original title, tests & measures,

mesh word] 9556

43 exp Observation Methods/ or exp Participant Observation/

or participant observation*.mp. 14655

44 exp Hermeneutics/ or hermeneutic*.mp. 8097

45 phenomenol*.mp. or exp Phenomenology/ 49642

46 lived experience*.mp. 17856

47 grounded theory.mp. or exp Grounded Theory/ 17882

48 constant compar*.mp. 5314

49 theoretical sampl*.mp. [mp=title, abstract, heading word,

table of contents, key concepts, original title, tests & measures,

mesh word] 645

50 content analy*.mp. 33645

51 thematic analy*.mp. [mp=title, abstract, heading word,

table of contents, key concepts, original title, tests & measures,

mesh word] 19864

52 narrative*.mp. [mp=title, abstract, heading word, t

able of contents, key concepts, original title, tests & measures,

mesh word] 77505

53 unstructured categor*.mp. 5

54 structured categor*.mp. 25

55 unstructured interview*.mp. 1144

56 exp Semi-Structured Interview/ or semi-structured interview*.mp. 39268

57 maximum variation*.mp. 376

58 snowball.mp. 2749

59 audio.mp. or exp Audiotapes/ 16369

60 tape*.mp. 15836

61 video*.mp. 74760

62 purposive sampl*.mp. [mp=title, abstract, heading word,

table of contents, key concepts, original title, tests & measures,

mesh word] 6001

63 action research.mp. or exp Action Research/ 10484

64 exp Focus Group/ or focus group*.mp. 42585

65 28 or 29 or 30 or 31 or 32 or 33 or 34 or 35 or 36 or 37 or 38 or 39

or 40 or 41 or 42 or 43 or 44 or 45 or 46 or 47 or 48 or 49 or 50

or 51 or 52 or 53 or 54 or 55 or 56 or 57 or 58 or 59 or 60 or 61

or 62 or 63 or 64 1463878

66 ((Timing or time or Plan* or Delay* or (late adj3 onset)

or Late-onset or Postpon* or advance*) adj3 (Motherhood

or Mothering or Parenthood or Parenting or Childbearing

or Child-bearing or (family adj2 formation*)

or (maternal adj2 age))).mp. [mp=title, abstract, heading word,

table of contents, key concepts, original title, tests & measures,

mesh word] 2093

67 ((Fertility or (biological adj2 clock*) or (Age* adj2 fertil*)

or (fertil* adj2 declin*) or (reproduc* adj2 age*))

adj3 (Consider* or Thought* or Aware* or Cogniti* or Knowledg*

or Attitud* or Reflect* or Perception* or Perceiv* or Intent*

or Opinion* or Concern* or Expression of concern or Feel*

or Emotion* or View* or Preference* or Value* or Personal value

or Decision making or Acceptab* or Belief* or Believe* or Decide*

or Decision* or Experience*)).mp. [mp=title, abstract, heading word,

table of contents, key concepts, original title, tests & measures,

mesh word] 1318

68 27 and 65 and 66 717

69 65 and 67 583

70 68 or 69 1271

71 limit 70 to (danish or english or norwegian or swedish) 1200

72 limit 71 to ("0100 journal" or "0110 peer-reviewed journal"

or "0120 non-peer-reviewed journal"

or "0130 peer-reviewed status unknown" or "0200 book"

or "0240 authored book" or "0280 edited book"

or "0300 encyclopedia" or "0400 dissertation abstract"

or "0500 electronic collection") 1200

73 limit 72 to yr="2021 -Current" 50

**CINAHL (Ebsco) 01.02.22**

S71 (S1 AND S27 AND S65) OR (S65 AND S66) Limiters - Published Date:

20210101-20221231; Publication Type: Abstract, Book, Book Chapter,

Book Review, Case Study, Clinical Trial, Commentary, Corrected Article,

Doctoral Dissertation, Editorial, Interview, Journal Article, Masters Thesis,

Meta Analysis, Meta Synthesis, Randomized Controlled Trial, Research,

Review, Systematic Review, Teaching Materials; Language: Danish,

English, Norwegian, Swedish  530

S70 (S1 AND S27 AND S65) OR (S65 AND S66)Limiters - Publication Type:

Abstract, Book, Book Chapter, Book Review, Case Study, Clinical Trial,

Commentary, Corrected Article, Doctoral Dissertation, Editorial, Interview,

Journal Article, Masters Thesis, Meta Analysis, Meta Synthesis,

Randomized Controlled Trial, Research, Review, Systematic Review,

Teaching Materials; Language: Danish, English, Norwegian, Swedish  530
 
S69 (S1 AND S27 AND S65) OR (S65 AND S66)  5,715

S68 S65 AND S66  966

S67 S1 AND S27 AND S65  5,299

S66 TI ( ((Fertility OR (MH "Fertility+") OR (biological N2 clock*) OR

(MH "Biological Clocks") OR (Age* N2 fertil*) OR (fertil* N2 declin*) OR

(reproduc* N2 age*)) N3 (Consider* OR Thought* OR Aware* OR Cognition

OR Cogni* OR Knowledge OR Knowledg* OR Attitud* OR Reflect* OR

Perception* OR Perceiv* OR Intent* OR Opinion* OR Concern* OR Feel*

OR Emotion* OR View* OR Preference* OR Value* OR Valuation*

OR Acceptab* OR Belief* OR Believe* OR Decide* OR Decision* OR

Decision Making OR Experience*)) ) OR AB ( ((Fertility OR (biological N2 clock*)

OR (Age* N2 fertil*) OR (fertil* N2 declin*) OR (reproduc* N2 age*))

N3 (Consider* OR Thought* OR Aware* OR Cognition OR Cogni*

OR Knowledge OR Knowledg* OR Attitud* OR Reflect* OR Perception*

OR Perceiv* OR Intent* OR Opinion* OR Concern* OR Feel* OR Emotion*

OR View* OR Preference* OR Value* OR Valuation* OR Acceptab* OR Belief*

OR Believe* OR Decide* OR Decision* OR Decision Making OR Experience*)) )

OR SU ( ((Fertility OR (biological N2 clock*) OR (Age* N2 fertil*)

OR (fertil* N2 declin*) OR (reproduc* N2 age*)) N3 (Consider* OR Thought*

OR Aware* OR Cognition OR Cogni* OR Knowledge OR Knowledg*

OR Attitud* OR Reflect* OR Perception* OR Perceiv* OR Intent* OR Opinion*

OR Concern* OR Feel* OR Emotion* OR View* OR Preference* OR Value*

OR Valuation* OR Acceptab* OR Belief* OR Believe* OR Decide* OR Decision*

OR Decision Making OR Experience*)) )  2,519

S65 S28 OR S29 OR S30 OR S31 OR S32 OR S33 OR S34 OR S35 OR S36 OR S37

OR S38 OR S39 OR S40 OR S41 OR S42 OR S43 OR S44 OR S45 OR S46 OR S47

OR S48 OR S49 OR S50 OR S51 OR S52 OR S53 OR S54 OR S55 OR S56 OR S57

OR S58 OR S59 OR S60 OR S61 OR S62 OR S63 OR S64  1,146,764

S64 (MH "Focus Groups") OR "focus N1 group*"  47,553

S63 (MH "Action Research") OR "action N1 research"  6,934

S62 (MH "Purposive Sample") OR "purposive N1 sampl*"  34,668

S61 (MH "Videorecording+") OR "video*"  72,614

S60 "tape*"  14,587

S59 (MH "Audiorecording") OR "audio"  54,882

S58 (MH "Snowball Sample") OR "snowball"  9,113

S57 "maximum variation*"  538

S56 (MH "Semi-Structured Interview") OR "semi-structured N1 interview*"  71,050

S55 (MH "Unstructured Interview") OR "unstructured N1 interview*"  1,684

S54 (MH "Structured Categories") OR "structured N1 categor*"  28

S53 (MH "Unstructured Categories") OR "unstructured N1 categor*"  10

S52(MH "Narratives") OR (MH "Storytelling") OR (MH "Life Histories")

OR "narrative*"  55,120

S51 (MH "Thematic Analysis") OR "thematic N1 analy*"  73,427

S50 (MH "Content Analysis") OR "content N1 analy*"  39,595

S49 (MH "Theoretical Sample") OR "theoretical N1 sampl*"  1,833

S48 (MH "Constant Comparative Method") OR "constant N1 compar*"  7,436

S47 (MH "Grounded Theory") OR "grounded N1 theory"  17,080

S46 (MH "Life Experiences") OR "lived N1 experience*"  30,702

S45 (MH "Phenomenological Research") OR (MH "Phenomenology")

OR "phenomenol*"  26,786

S44 (MH "Phenomenology") OR "hermeneutic*"  7,723

S43 (MH "Participant Observation") OR "participant N1 observation*"  5,617

S42 (MH "observational methods") OR "participant N1 observ*"  14,395

S41 (MH "Field Studies") OR (MH "Fieldwork") OR "field N1 stud*"  6,448

S40 (MH "Field Notes") OR "fieldnote*"  8,322

S39 "field record*"  71

S38 (MH "Field Notes") OR (MH "Field Studies") OR "field N1 note*"  11,416

S37 "etic"  146

S36 "emic"  402

S35 (MH "Ethnonursing Research") OR "ethnonurs*"  312

S34 (MH "Ethnographic Research") OR "ethnog*"  14,104

S33 (MH "Ethnological Research") OR "ethnol*" OR (MH "Ethnology")  46,457

S32 (MH "Qualitative Studies+") OR "qualitative"  227,492

S31 "health care organization"  811

S30 "finding*  615,167

S29 (MH "Semi-Structured Interview") OR (MH "Unstructured Interview")

OR (MH "Structured Interview") OR (MH "Interviews+") OR "interview*"  356,276

S28 (MH "Qualitative Studies+") OR "qualitative*"  232,539

S27 S2 OR S3 OR S4 OR S5 OR S6 OR S7 OR S8 OR S9 OR S10 OR S11 OR S12

OR S13 OR S14 OR S15 OR S16 OR S17 OR S18 OR S19 OR S20 OR S21 OR S22

OR S23 OR S24 OR S25 OR S26  2,639,538

S26 Consider*  471,997

S25 "Thought*" 59,144

S24 (MH "Cognition+") OR "Aware*"  184,118

S23 "Cogniti*"  203,818

S22 (MH "Knowledge+") OR "Knowledg*"  279,290

S21 (MH "Attitude+") OR "Attitud*"  529,504

S20 (MH "Reflection/EI") OR "Reflect*"  155,374

S19 (MH "Perception+") OR "Perception*"  203,502

S18 "Perceiv*"  137,156

S17 (MH "Intention") OR "Intent*"  59,355

S16 "Opinion*"  49,210

S15 "Concern*"  212,969

S14 "Feel*"  64,768
S13 (MH "Emotions+")  155,992

S12 "Emotion*"  123,930

S11 "View*"  144,356

S10 "Preference*"  51,880

S9 "Value*"  539,731

S8 "Valuation*"  2,266

S7 "Acceptab*"  57,400

S6 "Belief*"  60,016

S5 "Believe*"  49,166

S4 "Decide*"  15,797

S3 (MH "Decision Making+") OR "Decision*"  258,329

S2 "Experience*"  501,146

S1 TI ( (Timing OR time OR (MH "Time+") OR Plan* OR Delay*

OR (MH "Delayed Onset") OR (late N3 onset) OR Late-onset OR Postpon*

OR advance*) N3 (Motherhood OR (MH "Motherhood") OR Mothering

OR Parenthood OR (MH "Parenthood+") OR Parenting OR (MH "Parenting")

OR Childbearing OR Child-bearing OR (family N2 formation*) OR (maternal N2 age)

OR (MH "Maternal Age+") ) ) OR AB ( (Timing OR time OR (MH "Time+") OR Plan*

OR Delay* OR (MH "Delayed Onset") OR (late N3 onset) OR Late-onset OR Postpon*

OR advance*) N3 (Motherhood OR (MH "Motherhood") OR Mothering

OR Parenthood OR (MH "Parenthood+") OR Parenting OR (MH "Parenting")

OR Childbearing OR Child-bearing OR (family N2 formation*) OR (maternal N1 age)

OR (MH "Maternal Age+"))

14,621

**SCOPUS 13.01.22**

( ( ( TITLE-ABS-KEY ( ( ( timing  OR  time  OR  plan*  OR  delay*  OR  ( late  W/3  onset )  OR  late-onset  OR  postpon*  OR  advance* )  W/3  ( motherhood  OR  mothering  OR  parenthood  OR  parenting  OR  childbearing  OR  child-bearing  OR  ( family  W/2  formation* )  OR  ( maternal  W/2  age ) ) ) ) ) )  AND  ( ( TITLE-ABS-KEY ( ( consider*  OR  thought*  OR  aware*  OR  cogniti*  OR  knowledg*  OR  attitud*  OR  reflect*  OR  perception*  OR  perceiv*  OR  intent*  OR  opinion*  OR  concern*  OR  feel*  OR  emotion*  OR  view*  OR  preference*  OR  value*  OR  valuation*  OR  acceptab*  OR  belief*  OR  believe*  OR  decide*  OR  decision*  OR  experience* ) ) ) )  AND  ( ( TITLE-ABS-KEY ( qualitative*  OR  interview*  OR  finding*  OR  ( health  AND  care  AND  organization )  OR  ethnol*  OR  ethnog*  OR  ethnonurs*  OR  emic  OR  etic  OR  ( field  AND NOT  e* )  OR  ( field  AND  record* )  OR  fieldnote*  OR  ( field  AND  stud* )  OR  ( participant  AND  observ* )  OR  ( participant  AND  observation* )  OR  hermeneutic*  OR  phenomenol*  OR  ( lived  AND  experience* )  OR  ( grounded  AND  theory )  OR  ( constant  AND  compar* )  OR  ( theoretical  AND  sampl* )  OR  ( content  AND  analy* )  OR  ( thematic  AND  analy* )  OR  narrative*  OR  ( unstructured  AND  categor* )  OR  ( structured  AND  categor* )  OR  ( unstructured  AND  interview* )  OR  ( semi-structured  AND  interview* )  OR  ( maximum  AND  variation* )  OR  snowball  OR  audio  OR  tape*  OR  video*  OR  ( purposive  AND  sampl* )  OR  ( action  AND  research )  OR  ( focus  AND  group* ) ) ) ) )  OR  ( ( ( TITLE-ABS-KEY ( ( fertility  OR  ( biological  W/2  clock* )  OR  ( age*  W/2  fertil* )  OR  ( fertil*  W/2  declin* )  OR  ( reproduc*  W/2  age* ) )  W/3  ( consider*  OR  thought*  OR  aware*  OR  cogniti*  OR  knowledg*  OR  attitud*  OR  reflect*  OR  perception*  OR  perceiv*  OR  intent*  OR  opinion*  OR  concern*  OR  feel*  OR  emotion*  OR  view*  OR  preference*  OR  value*  OR  valuation*  OR  acceptab*  OR  belief*  OR  believe*  OR  decide*  OR  decision*  OR  experience* ) ) ) )  AND  ( ( TITLE-ABS-KEY ( qualitative*  OR  interview*  OR  finding*  OR  ( health  AND  care  AND  organization )  OR  ethnol*  OR  ethnog*  OR  ethnonurs*  OR  emic  OR  etic  OR  ( field  AND NOT  e* )  OR  ( field  AND  record* )  OR  fieldnote*  OR  ( field  AND  stud* )  OR  ( participant  AND  observ* )  OR  ( participant  AND  observation* )  OR  hermeneutic*  OR  phenomenol*  OR  ( lived  AND  experience* )  OR  ( grounded  AND  theory )  OR  ( constant  AND  compar* )  OR  ( theoretical  AND  sampl* )  OR  ( content  AND  analy* )  OR  ( thematic  AND  analy* )  OR  narrative*  OR  ( unstructured  AND  categor* )  OR  ( structured  AND  categor* )  OR  ( unstructured  AND  interview* )  OR  ( semi-structured  AND  interview* )  OR  ( maximum  AND  variation* )  OR  snowball  OR  audio  OR  tape*  OR  video*  OR  ( purposive  AND  sampl* )  OR  ( action  AND  research )  OR  ( focus  AND  group* ) ) ) ) )  AND  ( LIMIT-TO ( PUBYEAR ,  2022 )  OR  LIMIT-TO ( PUBYEAR ,  2021 ) )

520

**UPDATED SEARCH 11.11.22**

**EMBASE (Ovid) 11.11.22**

Embase Classic+Embase <1947 to 2022 November 10>

1 Consider*.mp. 3384793

2 Thought*.mp. 424054

3 Aware*.mp. or exp awareness/ 433310

4 exp cognition/ or Cogniti*.mp. 3231922

5 exp knowledge/ or Knowledg*.mp. 1223901

6 exp attitude/ or Attitud*.mp. 1025833

7 Reflect*.mp. 883439

8 Perception*.mp. or exp perception/ 775365

9 Perceiv*.mp. 347935

10 Intent*.mp. 210764

11 Opinion*.mp. 195669

12 exp "expression of concern"/ or Concern*.mp. 1033128

13 Feel*.mp. 177702

14 emotion/ or Emotion*.mp. 406445

15 View*.mp. 737226

16 Preference*.mp. 253095

17 exp personal value/ or Value*.mp. 3417933

18 decision making/ or Valuation*.mp. 281778

19 Acceptab*.mp. 304172

20 Belief*.mp. 131131

21 Believe*.mp. 342441

22 Decide*.mp. 111911

23 Decision*.mp. 895523

24 Experience*.mp. 1909306

25 1 or 2 or 3 or 4 or 5 or 6 or 7 or 8 or 9 or 10 or 11 or 12 or 13 or

14 or 15 or 16 or 17 or 18 or 19 or 20 or 21 or 22 or 23 or 24 13967223

26 qualitative*.mp. or exp qualitative research/ 481497

27 exp interview/ or exp semi structured interview/

or interview*.mp. 629049

28 finding*.mp. 3716060

29 health care organization.mp. or exp health care organization/ 2069545

30 exp ethnology/ or ethnol*.mp. 82477

31 exp ethnographic research/ or exp ethnography/ or ethnog*.mp. 15843

32 exp ethnonursing research/ or ethnonurs*.mp. 131

33 emic.mp. 870

34 etic.mp. 463

35 field note*.mp. 3408

36 field record*.mp. 820

37 fieldnote*.mp. 234

38 exp field study/ or field stud*.mp. 25544

39 exp participant observation/ or participant observ*.mp. 10797

40 participant observation*.mp. or participant observation/ 10550

41 exp hermeneutics/ or hermeneutic*.mp. 5088

42 exp phenomenology/ or phenomenol*.mp. 40182

43 lived experience*.mp. 11965

44 grounded theory.mp. or exp grounded theory/ 18167

45 exp constant comparative method/ or constant compar*.mp. 7679

46 exp theoretical sample/ or theoretical sampl*.mp. 1156

47 exp content analysis/ or content analy*.mp. 50917

48 exp thematic analysis/ or thematic analy*.mp. 46857

49 exp narrative/ or narrative*.mp. 75491

50 unstructured categor*.mp. 3

51 structured categor*.mp. 30

52 unstructured interview*.mp. 1247

53 exp semi structured interview/ or semi-structured interview*.mp. 78379

54 maximum variation*.mp. 1713

55 exp snowball sample/ or snowball.mp. 5580

56 exp audio recording/ or audio.mp. 34182

57 exp tape recorder/ or tape*.mp. 91009

58 video*.mp. 257549

59 exp purposive sample/ or purposive sampl*.mp. 15950

60 action research.mp. or exp action research/ 6683

61 focus group*.mp. 74698

62 26 or 27 or 28 or 29 or 30 or 31 or 32 or 33 or 34 or 35 or 36

or 37 or 38 or 39 or 40 or 41 or 42 or 43 or 44 or 45 or 46 or 47

or 48 or 49 or 50 or 51 or 52 or 53 or 54 or 55 or 56 or 57 or 58

or 59 or 60 or 61 6716863

63 ((Timing or time or Plan* or Delay* or (late adj3 onset) or

Late-onset or Postpon* or advance*) adj3 (Motherhood or

Mothering or Parenthood or Parenting or Childbearing or

Child-bearing or (family adj2 formation*)

or (maternal adj2 age))).mp. [mp=title, abstract, heading word,

drug trade name, original title, device manufacturer,

drug manufacturer, device trade name, keyword heading word,

floating subheading word, candidate term word] 8923

64 ((Fertility or (biological adj2 clock*) or (Age* adj2 fertil*)

or (fertil* adj2 declin*) or (reproduc* adj2 age*))

adj3 (Consider* or Thought* or Aware* or Cogniti*

or Knowledg* or Attitud* or Reflect* or Perception* or

Perceiv* or Intent* or Opinion* or Concern*

or Expression of concern or Feel* or Emotion* or View*

or Preference* or Value* or Personal value or

Decision making or Acceptab* or Belief* or Believe* or

Decide* or Decision* or Experience*)).mp.

[mp=title, abstract, heading word, drug trade name,

original title, device manufacturer, drug manufacturer,

device trade name, keyword heading word,

floating subheading word, candidate term word] 7012

65 25 and 62 and 63 1855

66 62 and 64 2748

67 65 or 66 4456

68 limit 67 to (danish or english or norwegian or swedish) 4360

69 limit 68 to (article or article in press or books or chapter

or editorial or "review") 2728

70 limit 69 to yr="2022" 230

**PSYINFO (Ovid) 11.11.22**

APA PsycInfo <1806 to October Week 5 2022>

1 Consider*.mp. 612998

2 Thought*.mp. 163896

3 exp Awareness/ or Aware*.mp. 233931

4 exp Cognitions/ or Cogniti*.mp. 757273

5 Knowledg*.mp. 374774

6 Attitud*.mp. 532531

7 Reflect*.mp. 276231

8 Perception*.mp. 585912

9 Perceiv*.mp. 302773

10 exp Intention/ or Intent*.mp. 113895

11 exp Attitudes/ or Opinion*.mp. 467506

12 Concern*.mp. 335470

13 Expression of concern.mp. 155

14 exp Emotions/ or Feel*.mp. 538585

15 Emotion*.mp. 472742

16 View*.mp. 336844

17 Preference*.mp. 127367

18 Value*.mp. or exp Values/ 407885

19 Personal value.mp. or exp Personal Values/ 5136

20 Decision making.mp. or exp Decision Making/ 191092

21 Acceptab*.mp. [mp=title, abstract, heading word,

table of contents, key concepts, original title, tests &

measures, mesh word] 48084

22 Belief*.mp. 157844

23 Believe*.mp. 92578

24 exp Decision Making/ or Decide*.mp. 159732

25 Decision*.mp. 258886

26 Experience*.mp. 777982

27 1 or 2 or 3 or 4 or 5 or 6 or 7 or 8 or 9 or 10 or 11 or 12 or

13 or 14 or 15 or 16 or 17 or 18 or 19 or 20 or 21 or 22 or 23

or 24 or 25 or 26 3654354

28 exp Qualitative Methods/ or qualitative*.mp. 229491

29 exp Focus Group Interview/ or interview*.mp.

or exp Semi-Structured Interview/ 467382

30 finding*.mp. 940129

31 health care organization.mp. 477

32 qualitative.mp. 212776

33 ethnol*.mp. 3611

34 ethnog*.mp. [mp=title, abstract, heading word,

table of contents, key concepts, original title, tests & measures,

mesh word] 33487

35 ethnonurs*.mp. [mp=title, abstract, heading word,

table of contents, key concepts, original title, tests & measures,

mesh word] 71

36 emic.mp. 1330

37 etic.mp. 751

38 field note*.mp. 4682

39 field record*.mp. 176

40 fieldnote*.mp. 432

41 field stud*.mp. 9296

42 participant observ*.mp. [mp=title, abstract, heading word,

table of contents, key concepts, original title, tests & measures,

mesh word] 9829

43 exp Observation Methods/ or exp Participant Observation/

or participant observation*.mp. 15145

44 exp Hermeneutics/ or hermeneutic*.mp. 8432

45 phenomenol*.mp. or exp Phenomenology/ 52484

46 lived experience*.mp. 19927

47 grounded theory.mp. or exp Grounded Theory/ 18748

48 constant compar*.mp. 5546

49 theoretical sampl*.mp. [mp=title, abstract, heading word,

table of contents, key concepts, original title, tests & measures,

mesh word] 677

50 content analy*.mp. 35763

51 thematic analy*.mp. [mp=title, abstract, heading word,

table of contents, key concepts, original title, tests & measures,

mesh word] 23148

52 narrative*.mp. [mp=title, abstract, heading word,

table of contents, key concepts, original title, tests & measures,

mesh word] 82459

53 unstructured categor*.mp. 5

54 structured categor*.mp. 25

55 unstructured interview*.mp. 1185

56 exp Semi-Structured Interview/ or semi-structured interview*.mp. 43247

57 maximum variation*.mp. 412

58 snowball.mp. 3071

59 audio.mp. or exp Audiotapes/ 17377

60 tape*.mp. 16026

61 video*.mp. 78618

62 purposive sampl*.mp. [mp=title, abstract, heading word,

table of contents, key concepts, original title, tests & measures,

mesh word] 6561

63 action research.mp. or exp Action Research/ 11159

64 exp Focus Group/ or focus group*.mp. 45231

65 28 or 29 or 30 or 31 or 32 or 33 or 34 or 35 or 36 or 37 or 38

or 39 or 40 or 41 or 42 or 43 or 44 or 45 or 46 or 47 or 48 or 49

or 50 or 51 or 52 or 53 or 54 or 55 or 56 or 57 or 58 or 59 or 60

or 61 or 62 or 63 or 64 1532742

66 ((Timing or time or Plan* or Delay* or (late adj3 onset) or

Late-onset or Postpon* or advance*) adj3 (Motherhood or

Mothering or Parenthood or Parenting or Childbearing or

Child-bearing or (family adj2 formation*) or

(maternal adj2 age))).mp. [mp=title, abstract, heading word,

table of contents, key concepts, original title, tests & measures,

mesh word] 2197

67 ((Fertility or (biological adj2 clock*) or (Age* adj2 fertil*) or

(fertil* adj2 declin*) or (reproduc* adj2 age*)) adj3 (Consider*

or Thought* or Aware* or Cogniti* or Knowledg* or Attitud*

or Reflect* or Perception* or Perceiv* or Intent* or Opinion*

or Concern* or Expression of concern or Feel* or Emotion*

or View* or Preference* or Value* or Personal value or

Decision making or Acceptab* or Belief* or Believe* or

Decide* or Decision* or Experience*)).mp.

[mp=title, abstract, heading word, table of contents, key concepts,

original title, tests & measures, mesh word] 1387

68 27 and 65 and 66 764

69 65 and 67 611

70 68 or 69 1345

71 limit 70 to (danish or english or norwegian or swedish) 1270

72 limit 71 to ("0100 journal" or "0110 peer-reviewed journal"

or "0120 non-peer-reviewed journal" or

"0130 peer-reviewed status unknown" or "0200 book" or

"0240 authored book" or "0280 edited book" or

"0300 encyclopedia" or "0400 dissertation abstract" or

"0500 electronic collection") 1270

73 limit 72 to yr="2022" 33

**MEDLINE (Ovid) 11.11.22**

Ovid MEDLINE(R) ALL <1946 to November 10, 2022>

1 Consider*.mp. 2429936

2 Thought*.mp. 300879

3 Aware*.mp. 292540

4 exp Cognition/ or Cogniti*.mp. 609696

5 exp Knowledge/ or Knowledg*.mp. 944330

6 Attitud*.mp. 473299

7 Reflect*.mp. 690782

8 exp Perception/ or Perception*.mp. 701963

9 Perceiv*.mp. [mp=title, book title, abstract, original title,

name of substance word, subject heading word,

floating sub-heading word, keyword heading word,

organism supplementary concept word,

protocol supplementary concept word,

rare disease supplementary concept word,

unique identifier, synonyms] 283203

10 Intent*.mp. 150247

11 Opinion*.mp. 141356

12 Concern*.mp. 739106

13 Feel*.mp. 114857

14 exp Emotions/ or Emotion*.mp. 554736

15 View*.mp. 536588

16 Preference*.mp. 199567

17 Value*.mp. 2521461

18 Valuation*.mp. 8451

19 Acceptab*.mp. [mp=title, book title, abstract, original title,

name of substance word, subject heading word,

floating sub-heading word, keyword heading word,

organism supplementary concept word,

protocol supplementary concept word,

rare disease supplementary concept word,

unique identifier, synonyms] 218261

20 Belief*.mp. 99992

21 Believe*.mp. 245790

22 Decide*.mp. or exp Decision Making/ 286853

23 Decision*.mp. or Decision Making/ 554783

24 Experience*.mp. 1290427

25 1 or 2 or 3 or 4 or 5 or 6 or 7 or 8 or 9 or 10 or 11 or 12

or 13 or 14 or 15 or 16 or 17 or 18 or 19 or 20 or 21 or 22

or 23 or 24 9825736

26 exp Qualitative Research/ or qualitative*.mp. 371619

27 interview*.mp. or exp Interview/ 465768

28 finding*.mp. 2799727

29 health care organization.mp. 1441

30 exp Qualitative Research/ or qualitative.mp. 320880

31 exp Ethnology/ or ethnol*.mp. 174588

32 ethnog*.mp. [mp=title, book title, abstract, original title,

name of substance word, subject heading word,

floating sub-heading word, keyword heading word,

organism supplementary concept word,

protocol supplementary concept word,

rare disease supplementary concept word,

unique identifier, synonyms] 13529

33 ethnonurs*.mp. [mp=title, book title, abstract, original title,

name of substance word, subject heading word,

floating sub-heading word, keyword heading word,

organism supplementary concept word,

protocol supplementary concept word,

rare disease supplementary concept word,

unique identifier, synonyms] 125

34 emic.mp. 698

35 etic.mp. 263

36 field note*.mp. 2825

37 field record*.mp. 687

38 fieldnote*.mp. 191

39 field stud*.mp. 17679

40 participant observ*.mp. 5326

41 participant observation*.mp. 5136

42 hermeneutic*.mp. or exp Hermeneutics/ 4515

43 phenomenol*.mp. 32271

44 lived experience*.mp. 10143

45 grounded theory.mp. or exp Grounded Theory/ 14473

46 constant compar*.mp. 6102

47 theoretical sampl*.mp. 876

48 content analy*.mp. 42115

49 thematic analy*.mp. [mp=title, book title, abstract, original title,

name of substance word, subject heading word,

floating sub-heading word, keyword heading word,

organism supplementary concept word,

protocol supplementary concept word,

rare disease supplementary concept word, unique identifier,

synonyms] 35036

50 narrative*.mp. 73846

51 unstructured categor*.mp. 3

52 structured categor*.mp. 24

53 unstructured interview*.mp. 892

54 semi-structured interview*.mp. 44987

55 maximum variation*.mp. 1362

56 snowball.mp. 4484

57 audio.mp. 60682

58 tape*.mp. 61778

59 video*.mp. 211447

60 purposive sampl*.mp. [mp=title, book title, abstract,

original title, name of substance word, subject heading word,

floating sub-heading word, keyword heading word,

organism supplementary concept word,

protocol supplementary concept word,

rare disease supplementary concept word,

unique identifier, synonyms] 11198

61 action research.mp. 5337

62 focus group*.mp. or exp Focus Groups/ 65702

63 26 or 27 or 28 or 29 or 30 or 31 or 32 or 33 or 34 or 35 or 36

or 37 or 38 or 39 or 40 or 41 or 42 or 43 or 44 or 45 or 46 or 47

or 48 or 49 or 50 or 51 or 52 or 53 or 54 or 55 or 56 or 57 or 58

or 59 or 60 or 61 or 62 3846975

64 ((Timing or time or Plan* or Delay* or (late adj3 onset)

or Late-onset or Postpon* or advance*) adj3 (Motherhood

or Mothering or Parenthood or Parenting or Childbearing

or Child-bearing or (family adj2 formation*)

or (maternal adj2 age))).mp. [mp=title, book title, abstract,

original title, name of substance word, subject heading word,

floating sub-heading word, keyword heading word,

organism supplementary concept word,

protocol supplementary concept word,

rare disease supplementary concept word,

unique identifier, synonyms] 6654

65 ((Fertility or (biological adj2 clock*) or (Age* adj2 fertil*)

or (fertil* adj2 declin*) or (reproduc* adj2 age*))

adj3 (Consider* or Thought* or Aware* or Cogniti*

or Knowledg* or Attitud* or Reflect* or Perception*

or Perceiv* or Intent* or Opinion* or Concern*

or Expression of concern or Feel* or Emotion* or View*

or Preference* or Value* or Personal value or Decision making

or Acceptab* or Belief* or Believe* or Decide* or Decision*

or Experience*)).mp. [mp=title, book title, abstract, original title,

name of substance word, subject heading word,

floating sub-heading word, keyword heading word,

organism supplementary concept word,

protocol supplementary concept word,

rare disease supplementary concept word,

unique identifier, synonyms] 5449

66 25 and 63 and 64 1081

67 63 and 65 1718

68 66 or 67 2729

69 limit 68 to (danish or english or norwegian or swedish) 2658

70 limit 69 to (adaptive clinical trial or case reports or classical article

or clinical study or clinical trial, all or clinical trial, phase i

or clinical trial, phase ii or clinical trial, phase iii or clinical trial,

phase iv or clinical trial or comment or comparative study

or controlled clinical trial or "corrected and republished article"

or duplicate publication or editorial or evaluation study

or government publication or guideline or interactive tutorial

or interview or introductory journal article or journal article

or lecture or meta analysis or multicenter study

or observational study or personal narrative or preprint

or randomized controlled trial or "review" or "systematic review") 2657

71 limit 70 to yr="2022" 217

**PROQUEST) 11.11.22**

(((TI((Fertility OR (biological NEAR/2 clock*) OR (Age* NEAR/2 fertil*) OR (fertil* NEAR/2 declin*) OR (reproduc* NEAR/2 age*)) NEAR/3 (Consider* OR Thought* OR Aware* OR Cogniti* OR Knowledg* OR Attitud* OR Reflect* OR Perception* OR Perceiv* OR Intent* OR Opinion* OR Concern* OR Feel* OR Emotion* OR View* OR Preference* OR Value* OR Valuation* OR Acceptab* OR Belief* OR Believe* OR Decide* OR Decision* OR Experience*)) OR AB((Fertility OR (biological NEAR/2 clock*) OR (Age* NEAR/2 fertil*) OR (fertil* NEAR/2 declin*) OR (reproduc* NEAR/2 age*)) NEAR/3 (Consider* OR Thought* OR Aware* OR Cogniti* OR Knowledg* OR Attitud* OR Reflect* OR Perception* OR Perceiv* OR Intent* OR Opinion* OR Concern* OR Feel* OR Emotion* OR View* OR Preference* OR Value* OR Valuation* OR Acceptab* OR Belief* OR Believe* OR Decide* OR Decision* OR Experience*))) AND (AB(qualitative* OR interview* OR finding* OR (health care organization) OR ethnol* OR ethnog* OR ethnonurs* OR emic OR etic OR (field note*) OR (field record*) OR fieldnote* OR (field stud*) OR (participant observ*) OR (participant observation*) OR hermeneutic* OR phenomenol* OR (lived experience*) OR (grounded theory) OR (constant compar*) OR (theoretical sampl*) OR (content analy*) OR (thematic analy*) OR narrative* OR (unstructured categor*) OR (structured categor*) OR (unstructured interview*) OR (semi-structured interview*) OR (maximum variation*) OR snowball OR audio OR tape* OR video* OR (purposive sampl*) OR (action research) OR (focus group*)) OR TI(qualitative* OR interview* OR finding* OR (health care organization) OR ethnol* OR ethnog* OR ethnonurs* OR emic OR etic OR (field note*) OR (field record*) OR fieldnote* OR (field stud*) OR (participant observ*) OR (participant observation*) OR hermeneutic* OR phenomenol* OR (lived experience*) OR (grounded theory) OR (constant compar*) OR (theoretical sampl*) OR (content analy*) OR (thematic analy*) OR narrative* OR (unstructured categor*) OR (structured categor*) OR (unstructured interview*) OR (semi-structured interview*) OR (maximum variation*) OR snowball OR audio OR tape* OR video* OR (purposive sampl*) OR (action research) OR (focus group*)) OR (mainsubject.Exact("qualitative research") OR mainsubject.Exact("interviews") OR mainsubject.Exact("ethnology") OR mainsubject.Exact("ethnography") OR mainsubject.Exact("hermeneutics") OR mainsubject.Exact("phenomenology" OR "phenomenological research") OR mainsubject.Exact("field study") OR mainsubject.Exact("grounded theory") OR mainsubject.Exact("content analysis") OR mainsubject.Exact("narratives" OR "narrative structure") OR mainsubject.Exact("audio recordings") OR mainsubject.Exact("videotape recordings" OR "video recordings" OR "video" OR "digital video") OR mainsubject.Exact("focus groups" OR "action research")))) OR ((TI((Timing OR time OR Plan* OR Delay* OR (late NEAR/3 onset) OR Late-onset OR Postpon* OR advance*) NEAR/3 (Motherhood OR Mothering OR Parenthood OR Parenting OR Childbearing OR Child-bearing OR (family NEAR/2 formation*) OR (maternal NEAR/2 age))) OR AB((Timing OR time OR Plan* OR Delay* OR (late NEAR/3 onset) OR Late-onset OR Postpon* OR advance*) NEAR/3 (Motherhood OR Mothering OR Parenthood OR Parenting OR Childbearing OR Child-bearing OR (family NEAR/2 formation*) OR (maternal NEAR/2 age)))) AND (TI((Consider* OR Thought* OR Aware* OR Cogniti* OR Knowledg* OR Attitud* OR Reflect* OR Perception* OR Perceiv* OR Intent* OR Opinion* OR Concern* OR Feel* OR Emotion* OR View* OR Preference* OR Value* OR Valuation* OR Acceptab* OR Belief* OR Believe* OR Decide* OR Decision* OR Experience*)) OR AB((Consider* OR Thought* OR Aware* OR Cogniti* OR Knowledg* OR Attitud* OR Reflect* OR Perception* OR Perceiv* OR Intent* OR Opinion* OR Concern* OR Feel* OR Emotion* OR View* OR Preference* OR Value* OR Valuation* OR Acceptab* OR Belief* OR Believe* OR Decide* OR Decision* OR Experience*)) OR (mainsubject.Exact("cognition") OR mainsubject.Exact("knowledge") OR mainsubject.Exact("attitudes") OR mainsubject.Exact("perceptions" OR "perception") OR mainsubject.Exact("opinions") OR mainsubject.Exact("emotions") OR mainsubject.Exact("preferences") OR mainsubject.Exact("values") OR mainsubject.Exact("valuation") OR mainsubject.Exact("acceptability") OR mainsubject.Exact("belief & doubt" OR "beliefs") OR mainsubject.Exact("decision making"))) AND (AB(qualitative* OR interview* OR finding* OR (health care organization) OR ethnol* OR ethnog* OR ethnonurs* OR emic OR etic OR (field note*) OR (field record*) OR fieldnote* OR (field stud*) OR (participant observ*) OR (participant observation*) OR hermeneutic* OR phenomenol* OR (lived experience*) OR (grounded theory) OR (constant compar*) OR (theoretical sampl*) OR (content analy*) OR (thematic analy*) OR narrative* OR (unstructured categor*) OR (structured categor*) OR (unstructured interview*) OR (semi-structured interview*) OR (maximum variation*) OR snowball OR audio OR tape* OR video* OR (purposive sampl*) OR (action research) OR (focus group*)) OR TI(qualitative* OR interview* OR finding* OR (health care organization) OR ethnol* OR ethnog* OR ethnonurs* OR emic OR etic OR (field note*) OR (field record*) OR fieldnote* OR (field stud*) OR (participant observ*) OR (participant observation*) OR hermeneutic* OR phenomenol* OR (lived experience*) OR (grounded theory) OR (constant compar*) OR (theoretical sampl*) OR (content analy*) OR (thematic analy*) OR narrative* OR (unstructured categor*) OR (structured categor*) OR (unstructured interview*) OR (semi-structured interview*) OR (maximum variation*) OR snowball OR audio OR tape* OR video* OR (purposive sampl*) OR (action research) OR (focus group*)) OR (mainsubject.Exact("qualitative research") OR mainsubject.Exact("interviews") OR mainsubject.Exact("ethnology") OR mainsubject.Exact("ethnography") OR mainsubject.Exact("hermeneutics") OR mainsubject.Exact("phenomenology" OR "phenomenological research") OR mainsubject.Exact("field study") OR mainsubject.Exact("grounded theory") OR mainsubject.Exact("content analysis") OR mainsubject.Exact("narratives" OR "narrative structure") OR mainsubject.Exact("audio recordings") OR mainsubject.Exact("videotape recordings" OR "video recordings" OR "video" OR "digital video") OR mainsubject.Exact("focus groups" OR "action research"))))) AND la.exact("ENG" OR "SWE") [Additional limits - Date: After 31 December 2020] 64

**CINAHL (EBSCO) 11.11.22**

| Friday, November 11, 2022 8:52:00 AM |
| --- |

| **#** | **Query** | **Limiters/Expanders** | **Results** |
| --- | --- | --- | --- |
| S69 | (S1 AND S27 AND S65) OR (S65 AND S66) | Limiters - Published Date: 20220101-20221231  Expanders - Apply equivalent subjects  Search modes - Boolean/Phrase | 410 |
| S68 | (S1 AND S27 AND S65) OR (S65 AND S66) | Limiters - Published Date: 20220101-20221231  Expanders - Apply equivalent subjects  Search modes - Boolean/Phrase | 410 |
| S67 | (S1 AND S27 AND S65) OR (S65 AND S66) | Limiters - Published Date: 20210101-20220131  Expanders - Apply equivalent subjects  Search modes - Boolean/Phrase | 557 |
| S66 | TI ( ((Fertility OR (MH "Fertility+") OR (biological N2 clock*) OR (MH "Biological Clocks") OR (Age* N2 fertil*) OR (fertil* N2 declin*) OR (reproduc* N2 age*)) N3 (Consider* OR Thought* OR Aware* OR Cognition OR Cogni* OR Knowledge OR Knowledg* OR Attitud* OR Reflect* OR Perception* OR Perceiv* OR Intent* OR Opinion* OR Concern* OR Feel* OR Emotion* OR View* OR Preference* OR Value* OR Valuation* OR Acceptab* OR Belief* OR Believe* OR Decide* OR Decision* OR Decision Making OR Experience*)) ) OR AB ( ((Fertility OR (biological N2 clock*) OR (Age* N2 fertil*) OR (fertil* N2 declin*) OR (reproduc* N2 age*)) N3 (Consider* OR Thought* OR Aware* OR Cognition OR Cogni* OR Knowledge OR Knowledg* OR Attitud* OR Reflect* OR Perception* OR Perceiv* OR Intent* OR Opinion* OR Concern* OR Feel* OR Emotion* OR View* OR Preference* OR Value* OR Valuation* OR Acceptab* OR Belief* OR Believe* OR Decide* OR Decision* OR Decision Making OR Experience*)) ) OR SU ( ((Fertility OR (biological N2 clock*) OR (Age* N2 fertil*) OR (fertil* N2 declin*) OR (reproduc* N2 age*)) N3 (Consider* OR Thought* OR Aware* OR Cognition OR Cogni* OR Knowledge OR Knowledg* OR Attitud* OR Reflect* OR Perception* OR Perceiv* OR Intent* OR Opinion* OR Concern* OR Feel* OR Emotion* OR View* OR Preference* OR Value* OR Valuation* OR Acceptab* OR Belief* OR Believe* OR Decide* OR Decision* OR Decision Making OR Experience*)) ) | Expanders - Apply equivalent subjects  Search modes - Boolean/Phrase | Display |
| S65 | S28 OR S29 OR S30 OR S31 OR S32 OR S33 OR S34 OR S35 OR S36 OR S37 OR S38 OR S39 OR S40 OR S41 OR S42 OR S43 OR S44 OR S45 OR S46 OR S47 OR S48 OR S49 OR S50 OR S51 OR S52 OR S53 OR S54 OR S55 OR S56 OR S57 OR S58 OR S59 OR S60 OR S61 OR S62 OR S63 OR S64 | Expanders - Apply equivalent subjects  Search modes - Boolean/Phrase | Display |
| S64 | (MH "Focus Groups") OR "focus N1 group*" | Expanders - Apply equivalent subjects  Search modes - Boolean/Phrase | Display |
| S63 | (MH "Action Research") OR "action N1 research" | Expanders - Apply equivalent subjects  Search modes - Boolean/Phrase | Display |
| S62 | (MH "Purposive Sample") OR "purposive N1 sampl*" | Expanders - Apply equivalent subjects  Search modes - Boolean/Phrase | Display |
| S61 | (MH "Videorecording+") OR "video*" | Expanders - Apply equivalent subjects  Search modes - Boolean/Phrase | Display |
| S60 | "tape*" | Expanders - Apply equivalent subjects  Search modes - Boolean/Phrase | Display |
| S59 | (MH "Audiorecording") OR "audio" | Expanders - Apply equivalent subjects  Search modes - Boolean/Phrase | Display |
| S58 | (MH "Snowball Sample") OR "snowball" | Expanders - Apply equivalent subjects  Search modes - Boolean/Phrase | Display |
| S57 | "maximum variation*" | Expanders - Apply equivalent subjects  Search modes - Boolean/Phrase | Display |
| S56 | (MH "Semi-Structured Interview") OR "semi-structured N1 interview*" | Expanders - Apply equivalent subjects  Search modes - Boolean/Phrase | Display |
| S55 | (MH "Unstructured Interview") OR "unstructured N1 interview*" | Expanders - Apply equivalent subjects  Search modes - Boolean/Phrase | Display |
| S54 | (MH "Structured Categories") OR "structured N1 categor*" | Expanders - Apply equivalent subjects  Search modes - Boolean/Phrase | Display |
| S53 | (MH "Unstructured Categories") OR "unstructured N1 categor*" | Expanders - Apply equivalent subjects  Search modes - Boolean/Phrase | Display |
| S52 | (MH "Narratives") OR (MH "Storytelling") OR (MH "Life Histories") OR "narrative*" | Expanders - Apply equivalent subjects  Search modes - Boolean/Phrase | Display |
| S51 | (MH "Thematic Analysis") OR "thematic N1 analy*" | Expanders - Apply equivalent subjects  Search modes - Boolean/Phrase | Display |
| S50 | (MH "Content Analysis") OR "content N1 analy*" | Expanders - Apply equivalent subjects  Search modes - Boolean/Phrase | Display |
| S49 | (MH "Theoretical Sample") OR "theoretical N1 sampl*" | Expanders - Apply equivalent subjects  Search modes - Boolean/Phrase | Display |
| S48 | (MH "Constant Comparative Method") OR "constant N1 compar*" | Expanders - Apply equivalent subjects  Search modes - Boolean/Phrase | Display |
| S47 | (MH "Grounded Theory") OR "grounded N1 theory" | Expanders - Apply equivalent subjects  Search modes - Boolean/Phrase | Display |
| S46 | (MH "Life Experiences") OR "lived N1 experience*" | Expanders - Apply equivalent subjects  Search modes - Boolean/Phrase | Display |
| S45 | (MH "Phenomenological Research") OR (MH "Phenomenology") OR "phenomenol*" | Expanders - Apply equivalent subjects  Search modes - Boolean/Phrase | Display |
| S44 | (MH "Phenomenology") OR "hermeneutic*" | Expanders - Apply equivalent subjects  Search modes - Boolean/Phrase | Display |
| S43 | (MH "Participant Observation") OR "participant N1 observation*" | Expanders - Apply equivalent subjects  Search modes - Boolean/Phrase | Display |
| S42 | (MH "observational methods") OR "participant N1 observ*" | Expanders - Apply equivalent subjects  Search modes - Boolean/Phrase | Display |
| S41 | (MH "Field Studies") OR (MH "Fieldwork") OR "field N1 stud*" | Expanders - Apply equivalent subjects  Search modes - Boolean/Phrase | Display |
| S40 | (MH "Field Notes") OR "fieldnote*" | Expanders - Apply equivalent subjects  Search modes - Boolean/Phrase | Display |
| S39 | "field record*" | Expanders - Apply equivalent subjects  Search modes - Boolean/Phrase | Display |
| S38 | (MH "Field Notes") OR (MH "Field Studies") OR "field N1 note*" | Expanders - Apply equivalent subjects  Search modes - Boolean/Phrase | Display |
| S37 | "etic" | Expanders - Apply equivalent subjects  Search modes - Boolean/Phrase | Display |
| S36 | "emic" | Expanders - Apply equivalent subjects  Search modes - Boolean/Phrase | Display |
| S35 | (MH "Ethnonursing Research") OR "ethnonurs*" | Expanders - Apply equivalent subjects  Search modes - Boolean/Phrase | Display |
| S34 | (MH "Ethnographic Research") OR "ethnog*" | Expanders - Apply equivalent subjects  Search modes - Boolean/Phrase | Display |
| S33 | (MH "Ethnological Research") OR "ethnol*" OR (MH "Ethnology") | Expanders - Apply equivalent subjects  Search modes - Boolean/Phrase | Display |
| S32 | (MH "Qualitative Studies+") OR "qualitative" | Expanders - Apply equivalent subjects  Search modes - Boolean/Phrase | Display |
| S31 | "health care organization" | Expanders - Apply equivalent subjects  Search modes - Boolean/Phrase | Display |
| S30 | "finding* | Expanders - Apply equivalent subjects  Search modes - Boolean/Phrase | Display |
| S29 | (MH "Semi-Structured Interview") OR (MH "Unstructured Interview") OR (MH "Structured Interview") OR (MH "Interviews+") OR "interview*" | Expanders - Apply equivalent subjects  Search modes - Boolean/Phrase | Display |
| S28 | (MH "Qualitative Studies+") OR "qualitative*" | Expanders - Apply equivalent subjects  Search modes - Boolean/Phrase | Display |
| S27 | S2 OR S3 OR S4 OR S5 OR S6 OR S7 OR S8 OR S9 OR S10 OR S11 OR S12 OR S13 OR S14 OR S15 OR S16 OR S17 OR S18 OR S19 OR S20 OR S21 OR S22 OR S23 OR S24 OR S25 OR S26 | Expanders - Apply equivalent subjects  Search modes - Boolean/Phrase | Display |
| S26 | Consider* | Expanders - Apply equivalent subjects  Search modes - Boolean/Phrase | Display |
| S25 | "Thought*" | Expanders - Apply equivalent subjects  Search modes - Boolean/Phrase | Display |
| S24 | (MH "Cognition+") OR "Aware*" | Expanders - Apply equivalent subjects  Search modes - Boolean/Phrase | Display |
| S23 | "Cogniti*" | Expanders - Apply equivalent subjects  Search modes - Boolean/Phrase | Display |
| S22 | (MH "Knowledge+") OR "Knowledg*" | Expanders - Apply equivalent subjects  Search modes - Boolean/Phrase | Display |
| S21 | (MH "Attitude+") OR "Attitud*" | Expanders - Apply equivalent subjects  Search modes - Boolean/Phrase | Display |
| S20 | (MH "Reflection/EI") OR "Reflect*" | Expanders - Apply equivalent subjects  Search modes - Boolean/Phrase | Display |
| S19 | (MH "Perception+") OR "Perception*" | Expanders - Apply equivalent subjects  Search modes - Boolean/Phrase | Display |
| S18 | "Perceiv*" | Expanders - Apply equivalent subjects  Search modes - Boolean/Phrase | Display |
| S17 | (MH "Intention") OR "Intent*" | Expanders - Apply equivalent subjects  Search modes - Boolean/Phrase | Display |
| S16 | "Opinion*" | Expanders - Apply equivalent subjects  Search modes - Boolean/Phrase | Display |
| S15 | "Concern*" | Expanders - Apply equivalent subjects  Search modes - Boolean/Phrase | Display |
| S14 | "Feel*" | Expanders - Apply equivalent subjects  Search modes - Boolean/Phrase | Display |
| S13 | (MH "Emotions+") | Expanders - Apply equivalent subjects  Search modes - Boolean/Phrase | Display |
| S12 | "Emotion*" | Expanders - Apply equivalent subjects  Search modes - Boolean/Phrase | Display |
| S11 | "View*" | Expanders - Apply equivalent subjects  Search modes - Boolean/Phrase | Display |
| S10 | "Preference*" | Expanders - Apply equivalent subjects  Search modes - Boolean/Phrase | Display |
| S9 | "Value*" | Expanders - Apply equivalent subjects  Search modes - Boolean/Phrase | Display |
| S8 | "Valuation*" | Expanders - Apply equivalent subjects  Search modes - Boolean/Phrase | Display |
| S7 | "Acceptab*" | Expanders - Apply equivalent subjects  Search modes - Boolean/Phrase | Display |
| S6 | "Belief*" | Expanders - Apply equivalent subjects  Search modes - Boolean/Phrase | Display |
| S5 | "Believe*" | Expanders - Apply equivalent subjects  Search modes - Boolean/Phrase | Display |
| S4 | "Decide*" | Expanders - Apply equivalent subjects  Search modes - Boolean/Phrase | Display |
| S3 | (MH "Decision Making+") OR "Decision*" | Expanders - Apply equivalent subjects  Search modes - Boolean/Phrase | Display |
| S2 | "Experience*" | Expanders - Apply equivalent subjects  Search modes - Boolean/Phrase | Display |
| S1 | TI ( (Timing OR time OR (MH "Time+") OR Plan* OR Delay* OR (MH "Delayed Onset") OR (late N3 onset) OR Late-onset OR Postpon* OR advance*) N3 (Motherhood OR (MH "Motherhood") OR Mothering OR Parenthood OR (MH "Parenthood+") OR Parenting OR (MH "Parenting") OR Childbearing OR Child-bearing OR (family N2 formation*) OR (maternal N2 age) OR (MH "Maternal Age+") ) ) OR AB ( (Timing OR time OR (MH "Time+") OR Plan* OR Delay* OR (MH "Delayed Onset") OR (late N3 onset) OR Late-onset OR Postpon* OR advance*) N3 (Motherhood OR (MH "Motherhood") OR Mothering OR Parenthood OR (MH "Parenthood+") OR Parenting OR (MH "Parenting") OR Childbearing OR Child-bearing OR (family N2 formation*) OR (maternal N1 age) OR (MH "Maternal Age+")) | Expanders - Apply equivalent subjects  Search modes - Boolean/Phrase | Display |

**SCOPUS 11.11.22**

## ( ( ( TITLE-ABS-KEY ( ( ( timing  OR  time  OR  plan*  OR  delay*  OR  ( late  W/3  onset )  OR  late-onset  OR  postpon*  OR  advance* )  W/3  ( motherhood  OR  mothering  OR  parenthood  OR  parenting  OR  childbearing  OR  child-bearing  OR  ( family  W/2  formation* )  OR  ( maternal  W/2  age ) ) ) ) ) )  AND  ( ( TITLE-ABS-KEY ( ( consider*  OR  thought*  OR  aware*  OR  cogniti*  OR  knowledg*  OR  attitud*  OR  reflect*  OR  perception*  OR  perceiv*  OR  intent*  OR  opinion*  OR  concern*  OR  feel*  OR  emotion*  OR  view*  OR  preference*  OR  value*  OR  valuation*  OR  acceptab*  OR  belief*  OR  believe*  OR  decide*  OR  decision*  OR  experience* ) ) ) )  AND  ( ( TITLE-ABS-KEY ( qualitative*  OR  interview*  OR  finding*  OR  ( health  AND  care  AND  organization )  OR  ethnol*  OR  ethnog*  OR  ethnonurs*  OR  emic  OR  etic  OR  ( field  AND NOT  e* )  OR  ( field  AND  record* )  OR  fieldnote*  OR  ( field  AND  stud* )  OR  ( participant  AND  observ* )  OR  ( participant  AND  observation* )  OR  hermeneutic*  OR  phenomenol*  OR  ( lived  AND  experience* )  OR  ( grounded  AND  theory )  OR  ( constant  AND  compar* )  OR  ( theoretical  AND  sampl* )  OR  ( content  AND  analy* )  OR  ( thematic  AND  analy* )  OR  narrative*  OR  ( unstructured  AND  categor* )  OR  ( structured  AND  categor* )  OR  ( unstructured  AND  interview* )  OR  ( semi-structured  AND  interview* )  OR  ( maximum  AND  variation* )  OR  snowball  OR  audio  OR  tape*  OR  video*  OR  ( purposive  AND  sampl* )  OR  ( action  AND  research )  OR  ( focus  AND  group* ) ) ) ) )  OR  ( ( ( TITLE-ABS-KEY ( ( fertility  OR  ( biological  W/2  clock* )  OR  ( age*  W/2  fertil* )  OR  ( fertil*  W/2  declin* )  OR  ( reproduc*  W/2  age* ) )  W/3  ( consider*  OR  thought*  OR  aware*  OR  cogniti*  OR  knowledg*  OR  attitud*  OR  reflect*  OR  perception*  OR  perceiv*  OR  intent*  OR  opinion*  OR  concern*  OR  feel*  OR  emotion*  OR  view*  OR  preference*  OR  value*  OR  valuation*  OR  acceptab*  OR  belief*  OR  believe*  OR  decide*  OR  decision*  OR  experience* ) ) ) )  AND  ( ( TITLE-ABS-KEY ( qualitative*  OR  interview*  OR  finding*  OR  ( health  AND  care  AND  organization )  OR  ethnol*  OR  ethnog*  OR  ethnonurs*  OR  emic  OR  etic  OR  ( field  AND NOT  e* )  OR  ( field  AND  record* )  OR  fieldnote*  OR  ( field  AND  stud* )  OR  ( participant  AND  observ* )  OR  ( participant  AND  observation* )  OR  hermeneutic*  OR  phenomenol*  OR  ( lived  AND  experience* )  OR  ( grounded  AND  theory )  OR  ( constant  AND  compar* )  OR  ( theoretical  AND  sampl* )  OR  ( content  AND  analy* )  OR  ( thematic  AND  analy* )  OR  narrative*  OR  ( unstructured  AND  categor* )  OR  ( structured  AND  categor* )  OR  ( unstructured  AND  interview* )  OR  ( semi-structured  AND  interview* )  OR  ( maximum  AND  variation* )  OR  snowball  OR  audio  OR  tape*  OR  video*  OR  ( purposive  AND  sampl* )  OR  ( action  AND  research )  OR  ( focus  AND  group* ) ) ) ) )  AND  ( LIMIT-TO ( PUBYEAR ,  2022 ) )

431
