# Supplementary material for: Women’s reflections on timing of motherhood: a meta-synthesis of qualitative evidence
Source: Reprod Health. 2023 Feb 8;20:30. doi: 10.1186/s12978-022-01548-x (PMC9909900; doi:10.1186/s12978-022-01548-x)
Supplement: Supplementary file 3 — Additional file 3: Appendix C: PRISMA Flowchart. [file 12978_2022_1548_MOESM3_ESM.docx]

**Additional file 3: Appendix C PRISMA FLOWCHART**

**Updated Literature search 13.01.22**

**Literature search 15.01.21-28.01.21**

Records removed *before screening*:

Duplicate records removed (n = 5816)

Records identified from:

Databases (n= 17323)

- Cinahl (n= 5143)

- Embase (n= 2252)

- Medline (n= 2264)

- Proquest (n= 1237)

- PsycInfo (n= 2011)

- Scopus (n= 4426)

**Identification**

Records excluded

(n = 11471)

Records screened

(n = 11517)

Reports not retrieved

(n = 0)

Reports sought for retrieval

(n = 45)

**Screening**

Reports excluded: (n = 37)

- Wrong population (n = 8)

- Wrong focus (n = 22)

- Wrong study design (n=5)

- Duplicates (n = 2)

Reports assessed for eligibility

(n = 45)

Studies included in review

(n = 8)

**Included**

Records removed *before screening*:

Duplicate records removed

(n = 665)

Records identified from:

Databases (n = 1653)

- Cinahl (n= 530)

- Embase (n= 279)

- Medline (n= 274)

- PsycInfo (n= 50)

- Scopus (n= 520)

Records excluded

(n = 985)

Records screened

(n = 988)

Reports not retrieved

(n = 0)

Reports sought for retrieval

(n = 3)

Reports excluded: (n = 3)

- Wrong population (n = 1)

- Wrong focus (n = 1)

- Wrong language (n = 1)

Reports assessed for eligibility

(n = 3)

Studies included in review

(n = 0)

**Updated literature search 11.11.22**

Records removed *before screening*:

Duplicate records removed (n = 508)

Records identified from*:

Databases (n= 1385)

- Cinahl (n= 410)

- Embase (n= 230)

- Medline (n= 217)

- Proquest (n= 64)

- PsycInfo (n= 33)

- Scopus (n= 431)

**Identification**

Records excluded

(n = 876)

Records screened

(n = 877)

**Screening**

Reports not retrieved

(n = 0)

Reports sought for retrieval

(n = 1)

Reports excluded: (n = 1)

- Wrong population (n = 1)

Reports assessed for eligibility

(n = 1)

Studies included in review

(n = 0)

**Included**
