# Supplementary material for: Women’s reflections on timing of motherhood: a meta-synthesis of qualitative evidence
Source: Reprod Health. 2023 Feb 8;20:30. doi: 10.1186/s12978-022-01548-x (PMC9909900; doi:10.1186/s12978-022-01548-x)
Supplement: Supplementary file 4 — Additional file 4: Appendix D: Excluded studies and Interrater reliability. [file 12978_2022_1548_MOESM4_ESM.docx]

**Additional file 4: Appendix D LIST OF EXCLUDED STUDIES AND INTER-RATER RELIABILITY**

**EXCLUDED STUDIES (n = 41)**

| **Title** | **Authors** | **Published Year** | **Journal** | **Volume** | **Issue** | **Pages** | **DOI** | **Exclusion reason** |
| --- | --- | --- | --- | --- | --- | --- | --- | --- |
| **Fertility Decision-Making in the UK: Insights from a Qualitative Study among British Men and Women** | Brough, M.; Sheppard, P. | 2022 | Social Sciences | 11 | 409 | 1-22 | https://doi.org/ 10.3390/socsci11090409 | Wrong population |
| **The role of motherhood schemas and life transitions in reproductive intention formation** | Fletcher-Hildebrand, S; Lawson, K; Downe, P;  Bayly, M. | 2021 | Journal of Reproductive and Infant Psychology |  |  |  | [10.1080/02646838.2021.1892044](http://dx.doi.org/10.1080/02646838.2021.1892044) | Wrong focus |
| **The pros and cons of fertility awareness and information: a generational, Swedish perspective** | Bodin, M; Plantin, L; Elmerstig, E; Schmidt, L; Ziebe, S | 2021 | Human Fertility |  |  |  | [10.1080/14647273.2021.1968045](https://doi.org/10.1080/14647273.2021.1968045) | Wrong population |
| **Delaying the arrival of the first child? A qualitative study on the perceptions around childbearing postponement** | Hincapie, V. G.; Lopez, M. T. L. | 2021 | Pap. | 106 | 2 | 221-253 | 10.5565/rev/papers.2735 | Not available (language) |
| **The journey to solo motherhood – An explorative study** | Werner, A.; Funderskov, K. F.; Nielsen, M. K.; Mørkholm, H.; Danbjørg, D. B.; Rothmann, M. J. | 2021 | Sexual and Reproductive Healthcare | 27 |  |  | 10.1016/j.srhc.2020.100586 | Wrong focus |
| **Childbearing in Italy and Spain: Postponement Narratives** | Lebano, A.; Jamieson, L. | 2020 | Population and Development Review | 46 | 1 | 121-144 | 10.1111/padr.12313 | Wrong focus |
| **Delaying, debating and declining motherhood** | Martin, L. J. | 2020 | Culture, Health & Sexuality |  |  | 1-16 |  | Wrong study design |
| **Women's perceptions of fertility assessment and counselling 6 years after attending a Fertility Assessment and Counselling clinic in Denmark** | Koert, E.; Sylvest, R.; Vittrup, I.; Hvidman, H. W.; Birch Petersen, K.; Boivin, J.; Nyboe Andersen, A.; Schmidt, L. | 2020 | Human Reproduction Open |  |  |  |  | Wrong focus |
| **Fertility decisions in transition: young adults’ perceptions on fertility three decades apart in Spain** | Bueno, X. | 2020 | History of the Family | 25 | 3 | 386-405 | 10.1080/1081602X.2019.1686049 | Wrong population |
| **Running out of time: exploring women's motivations for social egg freezing** | Baldwin, Kylie; Culley, Lorraine; Hudson, Nicky; Mitchell, Helene | 2019 | Journal of Psychosomatic Obstetrics & Gynecology | 40 | 2 | 166-173 | 10.1080/0167482X.2018.1460352 | Wrong focus |
| **What is 'good timing' in parenthood? Young mothers' accounts of parenthood and its timing** | Wissö, T. | 2019 | Families, Relationships and Societies | 8 | 3 | 479-494 | 10.1332/204674318X15313161373029 | Wrong focus |
| **Time, Anticipation, and the Life Course: Egg Freezing as Temporarily Disentangling Romance and Reproduction** | Brown, Eliza; Patrick, Mary | 2018 | American Sociological Review | 83 | 5 | 959-982 | 10.1177/0003122418796807 | Wrong focus |
| **What to Expect when Graduate Student Couples Delay Expecting: A Qualitative Study** | Wertentheil, Atara; Ponterotto, Joseph G. | 2018 | (Fordham University) |  | 10828709 | 202 |  | Wrong focus |
| **Conceptualising women's motivations for social egg freezing and experience of reproductive delay** | Baldwin, K. | 2018 | Sociology of Health & Illness | 40 | 5 | 859-873 |  | Wrong focus |
| **Pushing for the perfect time: Social and biological fertility** | Martin, Lauren Jade | 2017 | Women's Studies International Forum | 62 |  | 91-98 |  | Wrong population |
| **The desire for a child among a sample of heterosexual Australian couples** | Riggs, D. W.; Bartholomaeus, C. | 2016 | Journal of Reproductive and Infant Psychology | 34 | 5 | 442-450 |  | Wrong population |
| **Perceptions of oocyte banking from women intending to circumvent age-related fertility decline** | de Groot, M.; Dancet, E.; Repping, S.; Goddijn, M.; Stoop, D.; van der Veen, F.; Gerrits, T. | 2016 | Acta Obstetricia et Gynecologica Scandinavica | 95 | 12 | 1396-1401 |  | Wrong focus |
| **“For some people it isn’t a choice, it’s just how it happens”: Accounts of “delayed” motherhood among middle-class women in the UK** | Budds, K.; Locke, A.; Burr, V. | 2016 | Feminism and Psychology | 26 | 2 | 170-187 | 10.1177/0959353516639615 | Wrong focus |
| **Examining the psychosocial determinants of women's decisions to delay childbearing** | Kearney, A. L.; White, K. M. | 2016 | Human Reproduction | 31 | 8 | 1776-1787 |  | Wrong study design |
| **Individual fertility assessment and counselling in women of reproductive age** | Petersen, K. B. | 2016 | Danish Medical Journal | 63 | 10 |  |  | Wrong study design |
| **Ice, ice, baby?: a sociological exploration of social egg freezing** | Baldwin, Kylie | 2016 | (De Montfort University, United Kingdom) |  | 10590535 |  |  | Wrong focus |
| **Reproductive decision-making in a macro-micro perspective** | Philipov, D.; Liefbroer, A. C.; Klobas, J. E. | 2015 | Springer |  |  | 1-178 | 10.1007/978-94-017-9401-5 | Wrong study design |
| **Fertility decision-making: a qualitative study in Scotland** | Chen, Zhong Eric | 2015 | (The University of Edinburgh) |  | 10134852 |  |  | Wrong population |
| **'Banking time': Egg freezing and the negotiation of future fertility** | Waldby, Catherine | 2015 | Culture, Health & Sexuality | 17 | 4 | 470-482 |  | Wrong focus |
| **Delayed motherhood in aspiring professional women: A biopsychosocial perspective** | Nguyen, Nina; Houston-Armstrong, Tina | 2015 | (Alliant International University) |  | 3726452 | 127 |  | Duplicate |
| **Reasoning about timing of wanting a child: A qualitative study of Nordic couples from fertility clinics** | Sol Olafsdottir, H.; Wikland, M.; Möller, A. | 2012 | Journal of Reproductive and Infant Psychology | 29 | 5 | 493-505 | 10.1080/02646838.2011.635298 | Duplicate |
| **A project for future life—Swedish women's thoughts on childbearing lacking experience of giving birth and parenthood** | Söderberg, Malin; Christensson, Kyllike; Lundgren, Ingela | 2012 | International Journal of Qualitative Studies on Health & Well-Being | 7 | 1 | N.PAG-N.PAG | 10.3402/qhw.v7i0.17318 | Wrong population |
| **Reasoning about timing of wanting a child: A qualitative study of Nordic couples from fertility clinics** | Olafsdottir, Helga Sol; Wikland, Matts; Moller, Anders | 2011 | Journal of Reproductive and Infant Psychology | 29 | 5 | 493-505 |  | Wrong population |
| **Deadline for parenthood: Fertility postponement and age norms in Poland** | Mynarska, M. | 2010 | European Journal of Population | 26 | 3 | 351-373 | 10.1007/s10680-009-9194-x | Wrong focus |
| **Delayed motherhood, childbearing motivation and psychological distress levels in females age 30 and above** | Kazaryants, Kimberly | 2010 | Dissertation Abstracts International: Section B: The Sciences and Engineering | 70 | 7-B | 4488 |  | Wrong study design |
| **Young Australian women's aspirations for work, marriage and family: 'I guess I am just another person who wants it all'** | Arthur, N.; Lee, C. | 2008 | Journal of Health Psychology | 13 | 5 | 589-596 | 10.1177/1359105308090931 | Wrong focus |
| **Contemporary women turning 30** | Klay, M. A. | 2008 | ( Pacifica Graduate Institute) |  | Ph.D. | 310 p-310 p |  | Wrong focus |
| **Job insecurity and the timing of parenthood: A comparison between Eastern and Western Germany** | Bernardi, L.; Klärner, A.; Lippe, H. | 2008 | European Journal of Population | 24 | 3 | 287-313 | 10.1007/s10680-007-9127-5 | Wrong focus |
| **To be or not to be a mother?: Women negotiating cultural representations of mothering** | Maher, Jane Maree; Saugeres, Lise | 2007 | Journal of  Sociology | 43 | 1 | 5-21 |  | Wrong population |
| **Factors influencing women's decisions about timing of motherhood** | Benzies, K.; Tough, S.; Tofflemire, K.; Frick, C.; Faber, A.; Newburn-Cook, C. | 2006 | JOGNN - Journal of Obstetric, Gynecologic, and Neonatal Nursing | 35 | 5 | 625-633 |  | Wrong population |
| **The influence of psychological forces on childbearing delay in women nearing the end of fecundity** | Slosar, Heather Kelly; Diamond, David | 2003 | (Alliant International University) |  | 3107835 | 232 |  | Wrong focus |
| **Maternal identity and early mothering behavior in previously infertile and never infertile women** | Dunnington, R. M.; Glazer, G. | 1991 | JOGNN: Journal of Obstetric, Gynecologic & Neonatal Nursing | 20 | 4 | 309-318 | 10.1111/j.1552-6909.1991.tb01694.x | Wrong focus |
| **A psychosocial investigation of factors women consider in their childbearing preferences** | Warnke, Melanie Ann; Cook, Ellen Piel | 1990 | (University of Cincinnati) |  | 9108626 | 387 |  | Wrong focus |
| **The adult developmental characteristics of women who postponed parenthood** | Leser, Anne; Boggs, David L. | 1989 | (The Ohio State University) |  | 9011214 | 225 |  | Wrong focus |
| **Perceived Risks and the Decision to Delay Childbearing** | Kamin, Susan | 1987 | (MGH Institute of Health Professions) |  | 1331541 | 96 |  | Wrong focus |
| **Delayed first-time parenthood: A small sample phenomenological study** | Lynch-Sauer, Judith M. | 1981 | Dissertation Abstracts International Section A: Humanities and Social Sciences | 42 | 5-A | 2034 |  | Wrong focus |

## INTER-RATER RELIABILITY

Title/abstract screening

Original search

| **Reviewer A** | **Reviewer B** | **A Yes,**  **B Yes** | **A Yes, B No** | **A No, B Yes** | **A No, B No** | **Proportionate Agreement** | **Yes Probability** | **No Probability** | **Random Agreement Probability** | **Cohen's Kappa** |
| --- | --- | --- | --- | --- | --- | --- | --- | --- | --- | --- |
| CGT | HMA | 46 | 97 | 296 | 11078 | 0.96588 | 0.00037 | 0.95826 | 0.95863 | 0.17525 |

Updated search

| **Reviewer A** | **Reviewer B** | **A Yes, B Yes** | **A Yes, B No** | **A No, B Yes** | **A No, B No** | **Proportionate Agreement** | **Yes Probability** | **No Probability** | **Random Agreement Probability** | **Cohen's Kappa** |
| --- | --- | --- | --- | --- | --- | --- | --- | --- | --- | --- |
| CGT | HMA | 0 | 19 | 2 | 967 | 0.97874 | 0.00004 | 0.97878 | 0.97882 | -0.00368 |

Updated search 11.22.22

| **Reviewer A** | **Reviewer B** | **A Yes,**  **B Yes** | **A Yes, B No** | **A No, B Yes** | **A No, B No** | **Proportionate Agreement** | **Yes Probability** | **No Probability** | **Random Agreement Probability** | **Cohen's Kappa** |
| --- | --- | --- | --- | --- | --- | --- | --- | --- | --- | --- |
| CGT | HMA | 1 | 19 | 6 | 851 | 0,97149 | 0,00018 | 0,9694 | 0,96958 | 0,06299 |

Full text screening

Original search

| **Reviewer A** | **Reviewer B** | **A**  **Include**  **B**  **Include** | **A**  **Include**  **B Exclude** | **A Exclude, B**  **Include** | **A Exclude, B**  **Exclude** | **Proportionate Agreement** | **Yes Probability** | **No Probability** | **Random Agreement Probability** | **Cohen's Kappa** |
| --- | --- | --- | --- | --- | --- | --- | --- | --- | --- | --- |
| CGT | HMA | 8 | 1 | 3 | 33 | 0.91111 | 0.04889 | 0.60444 | 0.65333 | 0.74359 |

Updated search 13.01.22

| **Reviewer A** | **Reviewer B** | **A**  **Include**  **B**  **Include** | **A**  **Include**  **B**  **Exclude** | **A**  **Exclude**  **B**  **Include** | **A Exclude**  **B Exclude** | **Proportionate Agreement** | **Yes Probability** | **No Probability** | **Random Agreement Probability** | **Cohen's Kappa** |
| --- | --- | --- | --- | --- | --- | --- | --- | --- | --- | --- |
| CGT | HMA | 0 | 0 | 0 | 3 | 1.0 | 0.0 | 1.0 | 1.0 | NaN |

Updated search 11.22.22

| **Reviewer A** | **Reviewer B** | **A Yes,**  **B Yes** | **A Yes, B No** | **A No, B Yes** | **A No, B No** | **Proportionate Agreement** | **Yes Probability** | **No Probability** | **Random Agreement Probability** | **Cohen's Kappa** |
| --- | --- | --- | --- | --- | --- | --- | --- | --- | --- | --- |
| CGT | HMA | 0 | 0 | 0 | 1 | 1 | 0 | 1 | 1 | NaN |
